# Highly Potent Phosphinic HIV‑1 Protease Inhibitors: Synthesis, In Vitro Evaluation, and Docking Studies

**DOI:** 10.1021/acsomega.5c09361

**Published:** 2025-12-10

**Authors:** Komal Hayat, Danwen Qiu, Yuanyuan Wang, Palmer Sivoko Imbenzi, Faez Iqbal Khan, Magdalini Matziari

**Affiliations:** † Department of Chemistry and Materials Science, School of Science, 122238 Xi’an Jiaotong-Liverpool University, 111 Ren’ai Road, SIP, Suzhou, Jiangsu Province 215123, P. R. China; ‡ Department of Biosciences and Bioinformatics, School of Science, 668947 Xi’an Jiaotong-Liverpool University, 111 Ren’ai Road, SIP, Suzhou, Jiangsu Province 215123, P. R. China

## Abstract

In the search for effective HIV-1 protease inhibitors, the design of symmetrical phosphinic pseudopeptides derived from a dual addition of hypophosphorous acid to acrylates (**PACs**) has emerged as a promising strategy due to their nonhydrolyzable nature and high affinity for this protease. In this study, we report the synthesis, *in vitro* evaluation, and docking studies of a series of new symmetrical phosphinic inhibitors, synthesized via simple and cost-effective procedures. This approach overcomes the complexities of previous methods and utilizes commercially or readily available reagents. The synthesized **PACs**, featuring various P2 and P2’ substituents, have been evaluated for their inhibitory activity against the HIV-1 protease. Among them, the PAC-Phe-Val derivative (**9c**) demonstrated potent inhibition, with an IC_50_ value of 33 nM, comparable to the FDA-approved Darunavir. Resolution of the isomers of **9c** revealed the most potent candidate, with an IC_50_ value of 1 nM. Molecular docking studies revealed strong hydrogen bonding interactions between PAC-Phe-Val and key active site residues, suggesting a stable and effective binding profile. The compound’s structure–activity relationship (SAR) was explored, identifying crucial features for its inhibitory potency. This work highlights the potential of symmetrical phosphinic pseudopeptides as HIV-1 protease inhibitors and provides a foundation for further development of these compounds as novel antiretroviral therapies. Future research will focus on optimizing the pharmacokinetic properties and evaluating resistance profiles, aiming to advance the next generation of HIV treatments.

## Graphical abstract

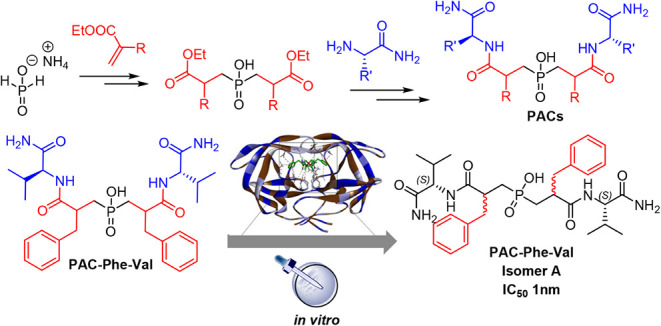

## Introduction

The Human Immunodeficiency Virus type 1 protease plays a vital role in the HIV virus replication, and it is widely recognized as a prominent target for the treatment of the human immunodeficiency virus (HIV) infection, in conjunction with other important targets, such as the reverse transcriptase and integrase enzymes.^1,2^ Due to the critical role of HIV-1 protease in viral maturation, it has become a prominent target in anti-HIV therapy, and the design and synthesis of protease inhibitors have undergone several rounds of optimization.

The HIV genome comprises the *gag*, *pol*, and *env* genes, which are essential for viral replication.^3^ These genes are expressed as polyproteins, which undergo enzymatic cleavage to produce the functional proteins of the mature virus. Genetic and biochemical studies have demonstrated that a virally encoded protease releases the protease, reverse transcriptase, integrase, and other proteins from the *gag-pol* fusion proteins.^4^ Importantly, the HIV-1 protease is essential for viral maturation; since site-directed mutagenesis of the protease’s active site aspartate residues (Asp25 and Asp25’) leads to the formation of noninfectious virions.^1^ Analysis of cleavage sites in the *gag*, *gag/pol*, and *pol* polyprotein, as well as in synthetic substrates, indicates that HIV-1 protease primarily recognizes hydrophobic side chains at the P1 and P1’ positions.^5,6^ Consequently, various inhibitor types—particularly those replacing the carboxamide moiety of the substrate with nonscissile tetrahedral “transition state” analogues^7–11^ —have been tested in enzyme assays and virally infected cells, revealing structural requirements for effective inhibition, with numerous reviews summarizing and rationalizing these findings.^12–14^ Currently, ten HIV-1 protease inhibitors have been approved by the FDA: Saquinavir, Indinavir, Nelfinavir, Amprenavir, Fosamprenavir, Lopinavir, Atazanavir, Tipranavir, Ritonavir, and Darunavir (last 2 shown in Figure 1).

**Figure 1:**
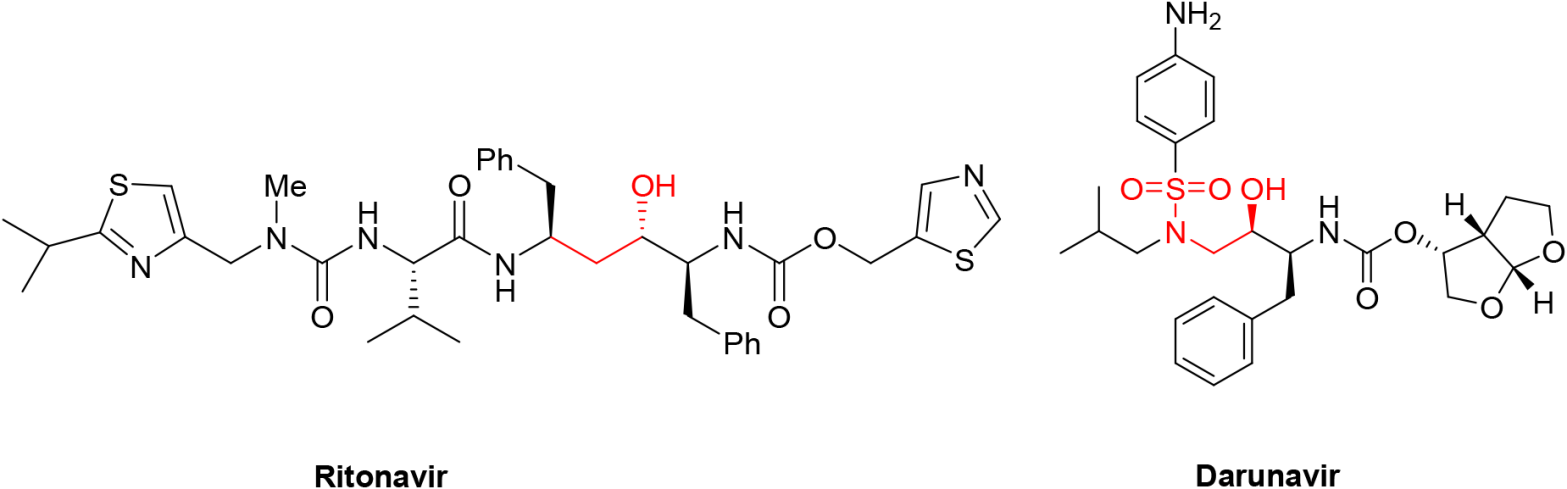
Chemical structures of FDA-approved HIV-1 protease inhibitors Ritonavir and Darunavir.

Unfortunately, most of these inhibitors are associated with side effects during long-term treatment. The most common side effects are HIV-1 protease inhibitor-induced metabolic syndromes, such as insulin resistance,^15^ proteasome inhibition,^16^ dyslipidemia,^17^ and lipodystrophy/lipoatrophy, as well as cardiovascular and cerebrovascular diseases.^18–21^ Thus, the need for novel antiretroviral therapies to combat HIV/AIDS persists, driven by challenges such as drug resistance, long-term toxicity, and treatment adherence.

In the search for potent inhibitors of HIV-1 protease, different structural modifications have been investigated, integrating various cores and substitutions to enhance inhibition.^22–25^ These compounds have demonstrated high potency against the enzyme, achieving nanomolar to subnanomolar IC_50_ values.^26^ In the pursuit of developing efficient inhibitors, phosphinic pseudopeptides have been examined as efficient and nonhydrolyzable isosters. The initial studies showed modest K*i* values, until the pioneering work of Dreyer et al.,^8^ and Grobelny et al. (Figure 2).^27^

**Figure 2:**
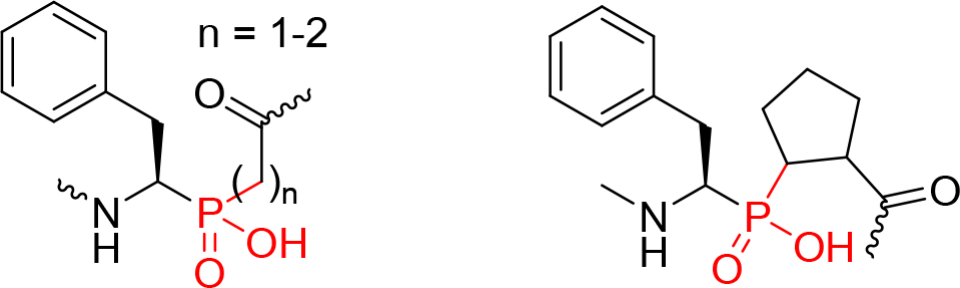
HIV-1 inhibitors incorporating a phosphinic isostere.

The design of the target structures is grounded in the fundamental enzymological principle that the strongest enzyme–inhibitor interactions occur with the transition state of the reaction, thereby reducing the activation energy.^28,29^ Phosphinic peptides are ideal transition-state mimics for proteases, as they resist hydrolysis, are metabolically stable, and maintain high affinity for their molecular targets, often surpassing other unstable classes, such as phosphonamidates. Moreover, given that HIV-1 protease is an aspartic, C2-symmetric, homodimeric enzyme, several studies based on the design of inhibitors with C2-symmetry components have been reported,^30–33^ leading to clinically successful drugs like Ritonavir (Figure 1).^34^ Despite the fact that this structure is not C2-symmetric, it was designed based on **A-77003**, a C2-symmetric lead structure (Figure 3).^35^ Using a similar approach, phosphinic acid–based inhibitors, developed by Abdel-Meguid et al. (**SB204144)** were found to be exceptionally well-suited for this target, as the central phosphinic moiety can form strong, symmetric interactions with the two catalytic aspartate residues (Figure 3).^36–38^ Another example of the inhibition of HIV-1 protease by symmetrical phosphinic acid–based inhibitors was published by Peyman et al.^30^ However, the limited availability of efficient methods for synthesizing phosphinic-based compounds, along with their inherent absorption issues, has restricted research in this area, leading to limited studies.

**Figure 3:**
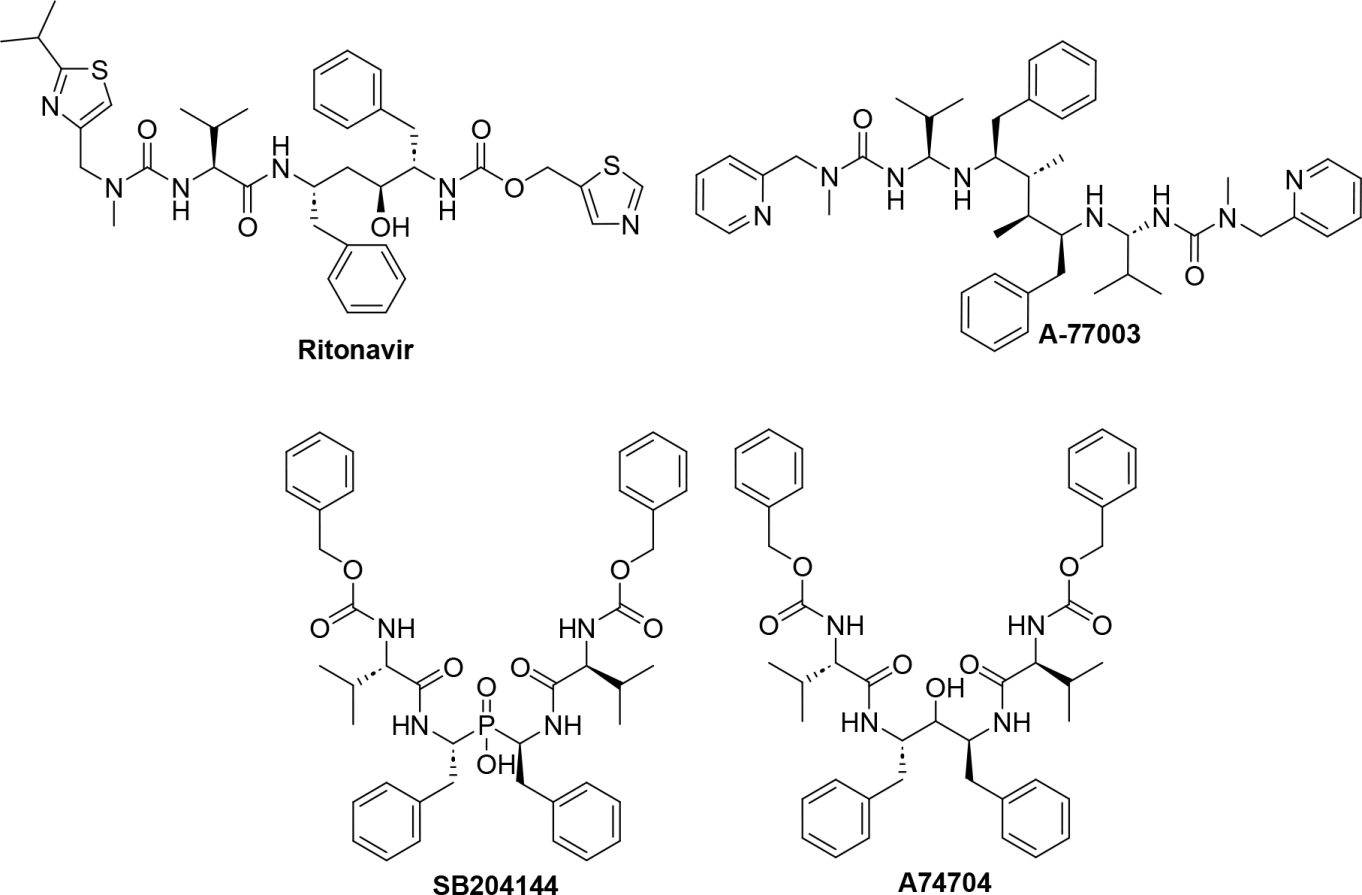
Various C2 symmetric and pseudosymmetric HIV inhibitors.

Despite this compelling rationale, research into phosphinic acid-based HIV-1 protease inhibitors has been limited. This is primarily due to challenges in their synthesis and inherent absorption issues. Herein, we report the design and synthesis of novel phosphinic pseudopeptides (abbreviated as PACs, Figure 4) inspired by the structural symmetry of HIV-1 proteaserather than natural substrate mimicry aimed at overcoming these limitations. Our work explores the design, synthesis, *in vitro* evaluation, and docking studies of new symmetrical scaffolds based on optimized backbone structures to develop potent inhibitors. The evaluation of these inhibitors provides new insights into the binding motifs of this promising inhibitor class.

**Figure 4:**
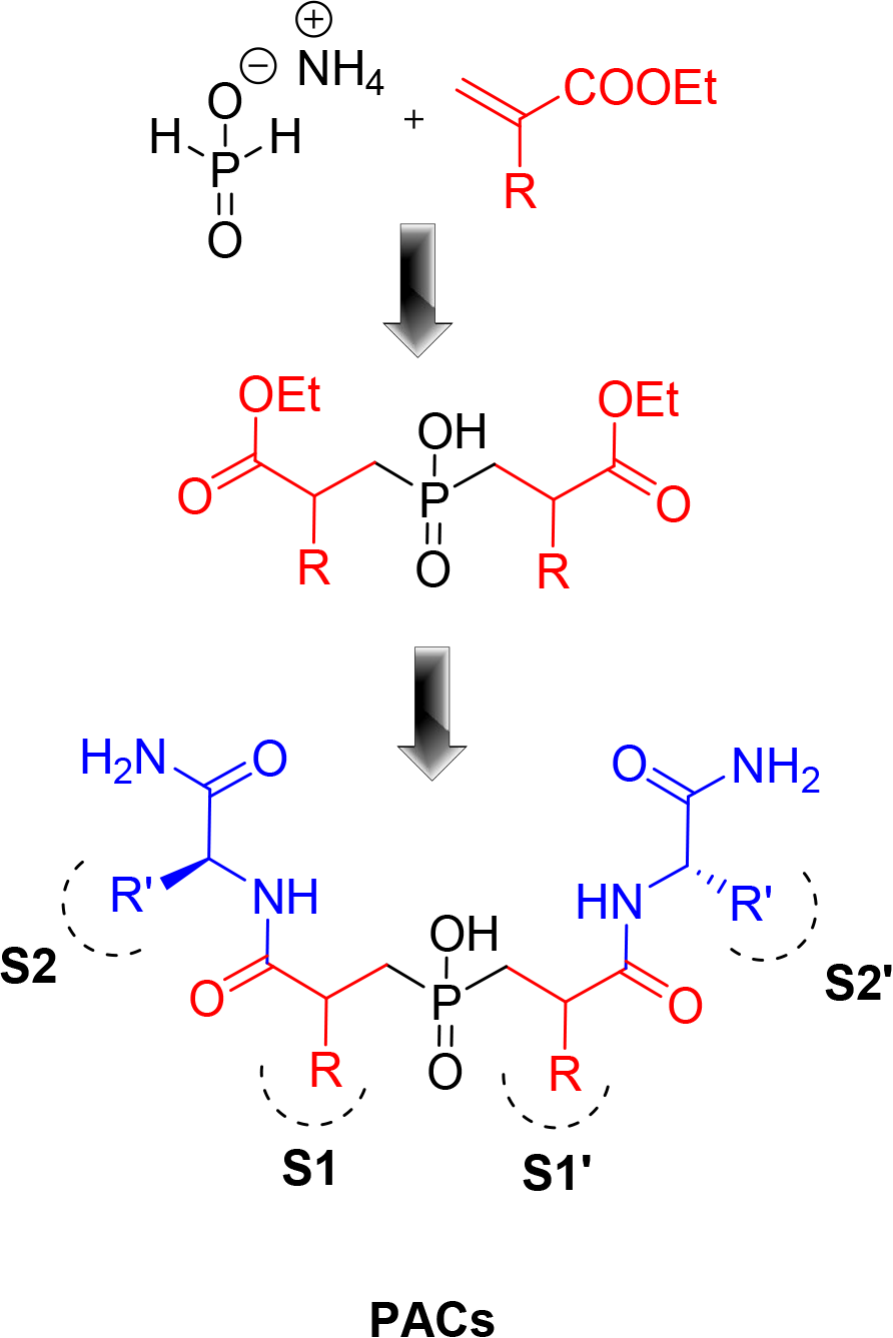
Generic representation synthetic scheme of potential HIV-1 protease inhibitors based on phosphinic acid-containing compounds (PACs).

Unlike prior methodologies reliant on multistep syntheses, our approach employs a high-yielding 6-step procedure, using commercially available or easily accessible starting materials, providing a time- and cost-effective access to **PACs**. Subsequent *in vitro* evaluation of these compounds against HIV-1 protease highlights their potential as potent inhibitors, reinforcing the value of symmetry-driven design and streamlined synthesis in accelerating antiretroviral drug discovery.

## Results and Discussion

### Chemistry

Our strategy for developing novel HIV-1 protease inhibitors was grounded in a rational design approach centered on the enzyme’s inherent C2 symmetry. We designed a series of phosphinic pseudopeptides (**PACs**) that move beyond traditional substrate mimicry to exploit instead the symmetric topology of the protease’s active site (Figure 4). The core of our design features a central phosphinic dipeptide scaffold that serves as a high-affinity transition-state analogue, strategically positioned between P1 and P1′ residues within a C2-symmetrical framework.^33,39,40^

To execute this design, we incorporated identical benzyl substituents at the P1/P1′ positions, informed by their proven ability to optimally occupy the symmetrical S1/S1′ subsites.^41^ We then focused our structure–activity relationship (SAR) investigations on the P2/P2′ positions, functionalizing them with a diverse set of hydrophobic amino acids to systematically probe their impact on affinity.^38,40,42^ This approach was guided by crystallographic evidence indicating that P1 and P2 side chains dominate deep subsite penetration and binding interactions,^38^ allowing us to focus the optimization rounds on these key determinants of affinity.

The synthesis of the target **PACs** was achieved via a streamlined protocol involving two main steps: (i) the quantitative preparation of pseudodipeptidic synthons **4a**–**e** and (ii) peptide elongation using standard peptide coupling. Building on our recently optimized procedure,^43^ we followed a simplified route to overcome the operational complexity and sensitivity of traditional phosphinic PI syntheses. Building on our optimized procedure,^43^ we first synthesized the symmetrical phosphinates **4a**–**e**.This was accomplished through sequential activation of ammonium hypophosphite (**1**) with hexamethyldisilazane (HMDS), followed by a Michael addition to acrylates **2** (Scheme 1). The subsequent P–C bond formation involved the *in situ* generation of trivalent bis­(trimethylsilyl) phosphorus species from the resulting phosphinic acids **3a**–**e** using trimethylsilyl chloride (TMSCl) as the activating agent, affording the target intermediates **4a**–**e** in high yield.^43^

**Scheme 1:**
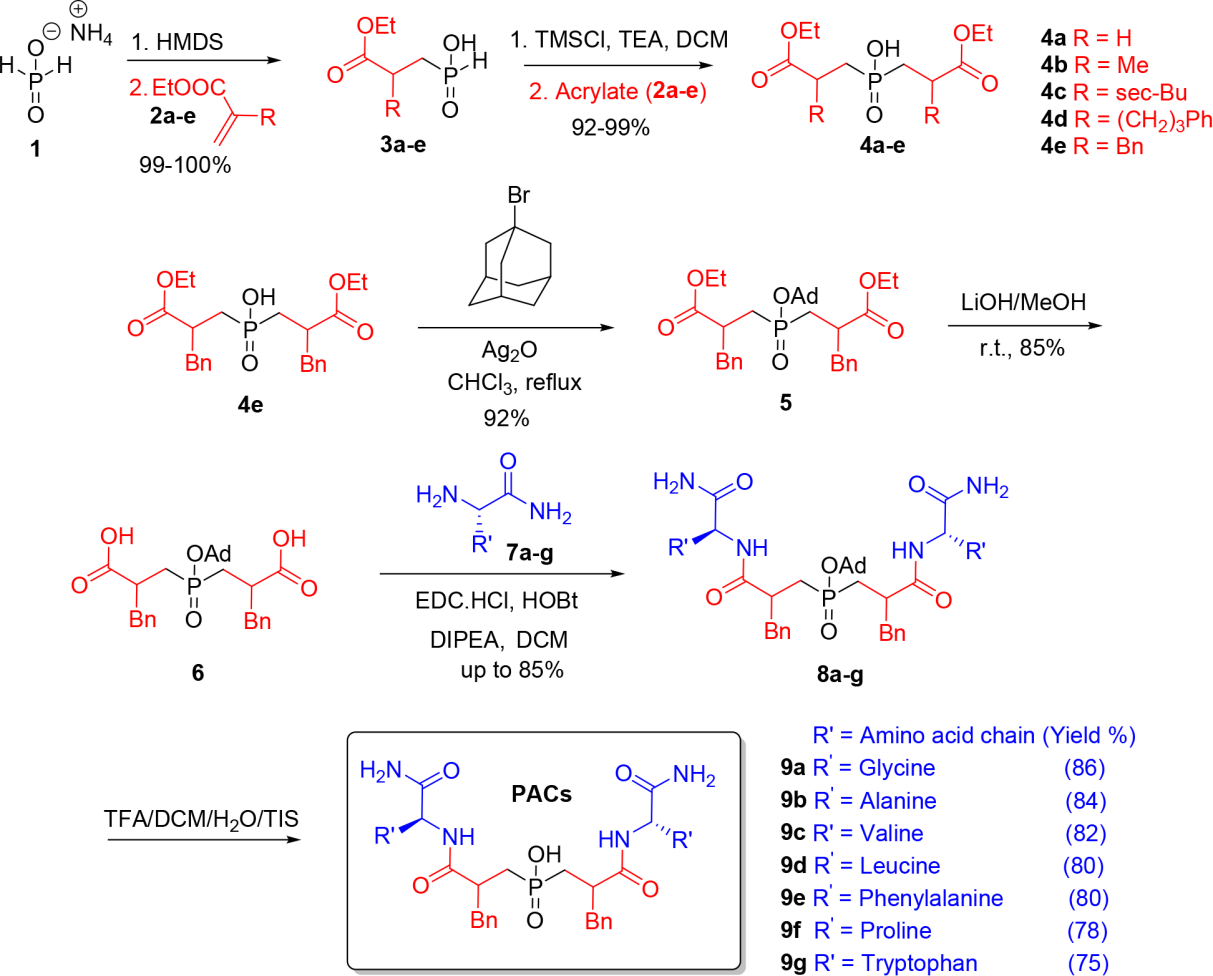
Synthesis of HIV-1 PIs (**PACs**)

Guided by prior studies highlighting the critical role of benzyl groups at the P1 and P1′ positions in phosphinic inhibitors, synthon **4e** was selected as the central building block for elongation. Initial attempts to couple the unprotected hydroxyphosphinyl group of **4e** with amino acids under standard conditions yielded inseparable byproducts, regardless of the coupling reagents tested. Therefore, considering numerous reports concerning the necessity for the protection of the hydroxyphosphinyl function on blocks of type **4**,^44^ we introduced the adamantyl group as a protecting moiety, among the various options, leveraging its efficient introduction and mild deprotection conditions, rendering it a promising choice.^45,46^ Therefore, the protection of the free hydroxyphosphinyl group was executed using 1-Bromoadamantane to yield **5**, followed by hydrolysis to afford terminal carboxylic phosphinate **6,** which was subsequently coupled with *L*-amino acid amides **7a**–**g** using the soluble carbodiimide EDC to afford the phosphinates **8a**–**g** in excellent yields up to 85%. This strategy bypassed the need for postcoupling amidation, thereby simplifying the synthetic workflow while mimicking the peptide backbone’s enzyme-binding interactions. Lastly, to restore the free hydroxyphosphinyl in the compounds, the deprotection was achieved using TFA/DCM/TIS/H_2_O, which furnished the C2-symmetric phosphinic acid dipeptides **9a**–**g** in excellent yields (Scheme 1). The resulting phosphinic pseudopeptides **(PACs)** bearing a variety of P2 and P2’ substituents were then subjected to *in vitro* evaluation and docking-based SAR studies.

### Results of In Vitro Evaluation against HIV-1 Inhibitory Activity

The commercially available potent HIV-1 protease inhibitor Darunavir (Figure 1) was used as the positive control. As shown in Figure 5A, Darunavir exhibited the highest level of enzyme inhibition among all tested compounds, achieving nearly complete suppression across the three selected concentrations. Notably, PAC-Phe-Val (**9c**) demonstrated a significant reduction in enzymatic activity compared to the other tested **PACs**, indicating its potential. At both 1 μM and 100 nM, **9c** displayed a pronounced inhibitory effect comparable to that of Darunavir, suggesting it may act through a similar mechanism of action. In contrast, PAC-Phe-Leu (**9d**) showed a moderate level of enzyme inhibition, which was less effective than PAC-Phe-Val (**9c**) and Darunavir. On the other hand, PAC-Phe-Ala (**9b**) and PAC-Phe-Phe (**9e**) exhibited only weak inhibition, indicating limited efficacy against the HIV-1 protease. Importantly, PAC-Phe-Gly (**9a**) and PAC-Phe-Pro (**9f**) did not exhibit any significant inhibitory effect at any concentration tested, highlighting the need to further explore the structural features contributing to inhibitory activity. Given the encouraging results observed for **9c**, its inhibitory potency was further characterized by determining the IC_50_ value, which was found to be 33.10 nM (95% CI: 22.18–51.01 nM). This result indicates a high potency against the wild-type HIV-1 protease, suggesting that **9c** could serve as a promising lead compound for further development.

**Figure 5:**
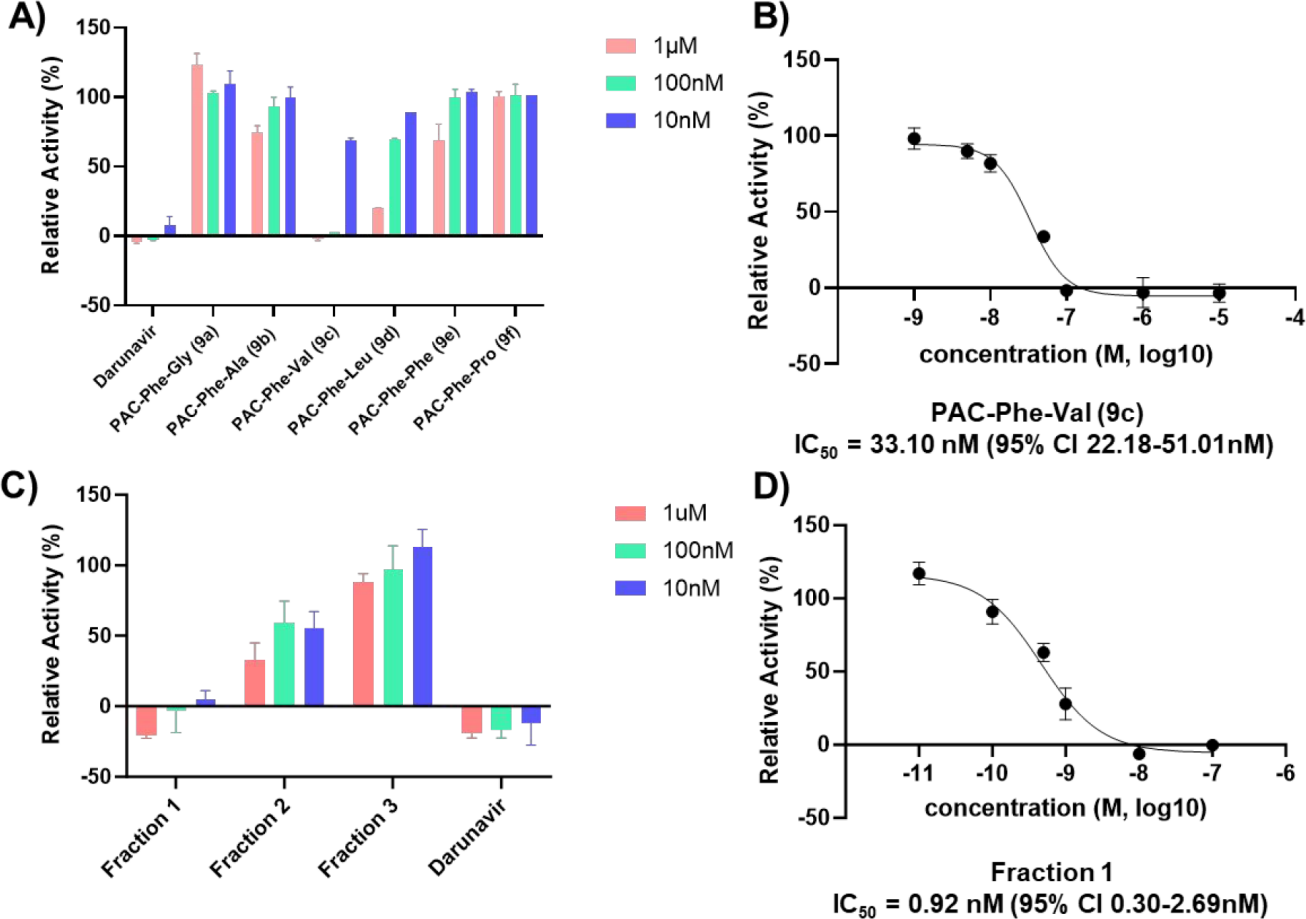
*In vitro* inhibitory activity of PACs. A) Relative activities of Darunavir and **PACs** at 1 μM, 100 nM and 10 nM. The bars represent means ± SD, *n* = 2. B) PAC-Phe-Val (**9c**) inhibited the activity of HIV-1 protease. The bars represent means ± SD, *n* = 3. C) Relative activities of the three isolated isomers of PAC-Phe-Val (**9c**) and Darunavir at 1 μM, 100 nM and 10 nM. D) Isomer 1 of PAC-Phe-Val (**9c**) inhibited the activity of HIV-1 protease. The bars represent means ± SD, *n* = 3.

Furthermore, we conducted the resolution of **9c** to its diastereoisomers, by means of preparative RP-HPLC. Three isomers were isolated, corresponding to the three diastereoisomers of **9c**, with the *SSRS* and *SRSS* being identical, due to the symmetry of the molecule, which are shown in Figure 6. The first eluted isomer, based on elution order, exhibited a remarkably higher inhibitory activity than the other two isomers, shown in Figure 5c, with an IC_50_ value as low as 0.92 nM (95% CI: 0.30–2.69 nM). This significant potency underscores the therapeutic potential of this isomer and warrants further investigation into its binding interactions with HIV-1 protease. To elucidate its mode of action, we employed docking and computational studies to analyze these interactions in detail.

**Figure 6:**
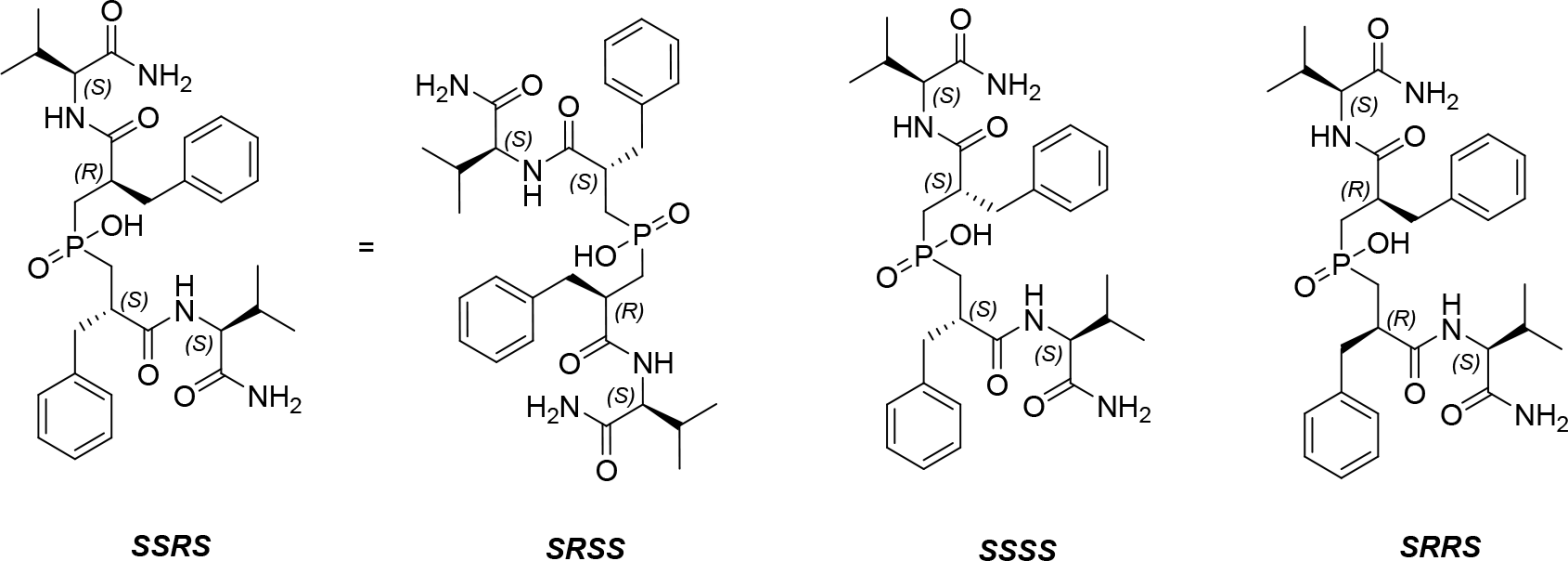
Isomers of PAC-Phe-Val (9c).

### Docking Studies of the Most Potent PAC Derivative: PAC-Phe-Val (9c)

#### Results of Molecular Interaction Analysis

Molecular docking is an essential tool for understanding the interactions between small molecules and macromolecules.^47,48^ It helps identify potential drug candidates by predicting the binding affinity of small molecules to a target protein.^49,50^ The binding modes of *SSSS*, *SRSS,* and *SRRS* of **9c** in HIV-1 protease were studied using molecular docking with the Glide XP (Schrodinger Suite, Version 2024.1) and Autodock Vina software.^51^ Active or binding sites on protein surfaces play a central role in protein function.^52^ Identifying these binding sites on the protein molecule is often the first step in studying protein function and in structure-based drug design.^53^

The catalytic dyad formed by Asp25 in chain A and its symmetry-related partner Asp125 in chain B was used to define the docking grid. The opposing carboxylate groups of these residues engage phosphinic pseudopeptide inhibitors through hydrogen bonds under conditions where one of the aspartic acid residues is protonated, establishing the primary anchoring site in the active pocket. As this dyad is conserved in all reported drug-resistant variants, a grid defined in this manner remains valid for mutant protease structures without further adjustment.^54^ It has been reported that upon inhibitor binding, the protease adopts a closed conformation with a flap-tip distance of approximately 0.59 nm.^55^ Therefore, all three isomers were docked into the binding pocket of the wild-type HIV-1 protease structure (PDB ID: 2IEN), whose high resolution of 1.30 Å provides accurately placed side chains for reliable hydrogen-bond scoring.^56^ Importantly, the structure already adopts the closed-flap geometry (Figure 7A), obviating the large induced-fit corrections required for semiopen conformations and ensuring that the docking simulations explore the catalytically relevant binding mode. Docking results revealed that all three isomers bind within the hydrophobic cavity of the protease (Figure 7B). Superimposition of the best docked poses indicated that all three isomers occupy a similar spatial region and exhibit nearly identical orientations, except *SSSS*, for which the Glide XP top-scoring pose is asymmetric, with both benzyl groups clustered on one side of the active site rather than occupying the C2-related P1 and P1’ pockets (Figure 7C).

**Figure 7:**
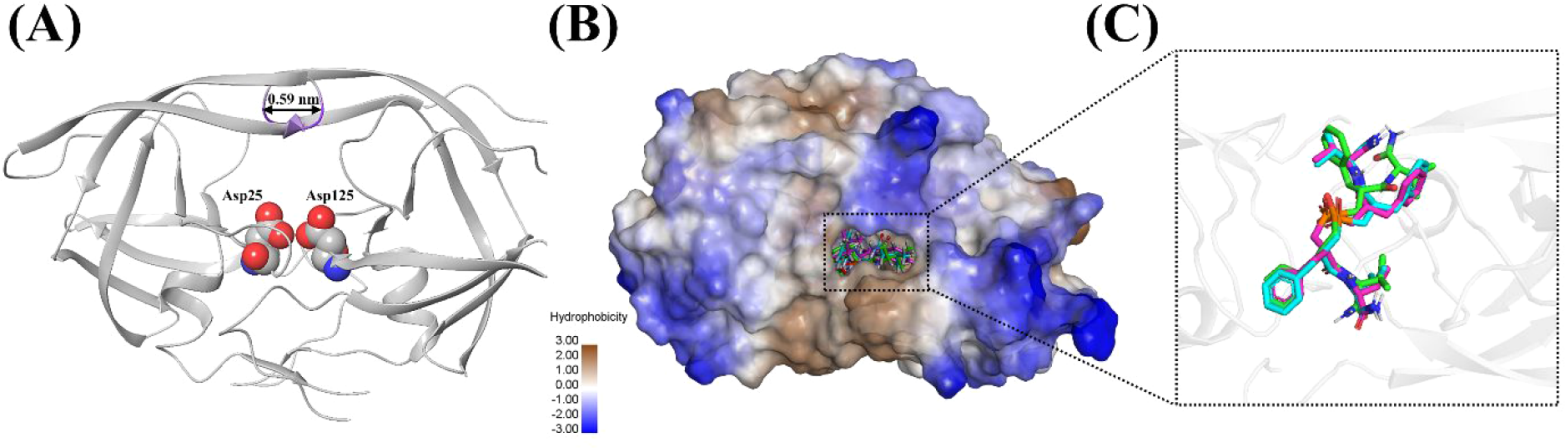
Docking modes of *SSSS*, *SRSS*, and *SRRS* with the HIV-1 Protease. (A) The catalytic dyad, Asp25 from chain A and Asp125 from chain B, is highlighted, and the enzyme is depicted in its closed conformation with a flap-tip separation of ∼0.59 nm. (B) Docked poses of the compounds *SSSS*, *SRSS*, and *SRRS* within the protease’s hydrophobic cavity, with color gradients indicating hydrophilicity. (C) Superimposition of the docked ligands showing all compounds align in a similar spatial region and exhibit comparable orientations within the binding site.

MM-GBSA binding free energy calculations offer the advantage of being more theoretically rigorous than empirical scoring functions.^57,58^ The binding affinities and docking scores for the best C2-symmetric pose for each isomer are listed in Table 1, while the MM-GBSA calculated binding free energies for the same poses are presented in Table 2. *SRRS* exhibited the lowest binding affinity toward HIV-1 protease, with a binding affinity of −10.6 kcal/mol and docking score of −5.559 kcal/mol. AutoDock Vina revealed that the *SRSS* and *SSSS* had comparable binding affinities of −11.9 kcal/mol, whereas Glide XP ranks *SSSS* highest, with a score of −6.895 kcal/mol. MM-GBSA binding energies, calculated using Glide XP poses, indicate that *SSSS* has the most negative binding affinity (−63.44 kcal/mol), followed closely by *SRSS* (−56.88 kcal/mol), with *SRRS* showing the least negative value (−48.41 kcal/mol).

**Table 1:** Binding Affinities and Docking Scores for Top Poses of the 3 Isomers Using AutoDock Vina and Glide XP

Compounds	AutoDock Vina Binding Affinity (kcal/mol)	Glide XP Docking Score (kcal/mol)
*SSSS*	–11.9	–6.895
*SRSS*	–11.9	–5.520
*SRRS*	–10.6	–5.559

**Table 2:** MM-GBSA Binding Free Energies for the Top Glide XP Docked Poses of the 3 Isomers

Compounds	SRSS	SSSS	SRRS
***Total Binding Energy** (kcal/mol)*	–56.88	–63.44	–48.41
***Electrostatic Energy (Coulomb)**(kcal/mol)*	–33.35	–21.03	–17.79
***Covalent Energy** (kcal/mol)*	7.49	3.94	5.85
***Hydrogen Bonding Energy** (kcal/mol)*	–3.14	–3.59	–1.46
***Lipophilic Energy** (kcal/mol)*	–22.93	–23.33	–25.15
***Packing Energy** (kcal/mol)*	0	–0.07	0
***Solvation Energy (GB)** (kcal/mol)*	55.12	55.34	54.86
***van der Waals Energy** (kcal/mol)*	–60.07	–74.69	–64.72

Several residues, including Gly52, Phe53, Ile54, Thr80, Pro81, Val82, and Ile84 play important roles within the active site. Conserved residues such as Gly27, Asp29, Asp30, and Gly48 are critical for maintaining the structural integrity of the protease and serve as key contact points for inhibitors.^59^ The protein–compound interactions of the best poses generated by Glide XP are shown in Figure 8. 2D view interactions revealed that *SRSS* forms several hydrogen bonds with residues Asp25chainA, Asp29chainA, Gly48ChainA, Ile50chainA, and Gly127chainB (Figure 8A). *SSSS* primarily interacts through hydrogen bonds with Ile50chainA, Asp29chainA-Asp129chainB, and Gly48chainA-Gly148chainB (Figure 8C). In contrast, *SRRS* mainly interacts with HIV-1 protease through hydrogen bonds with Gly27chainA (Figure 8E).

**Figure 8:**
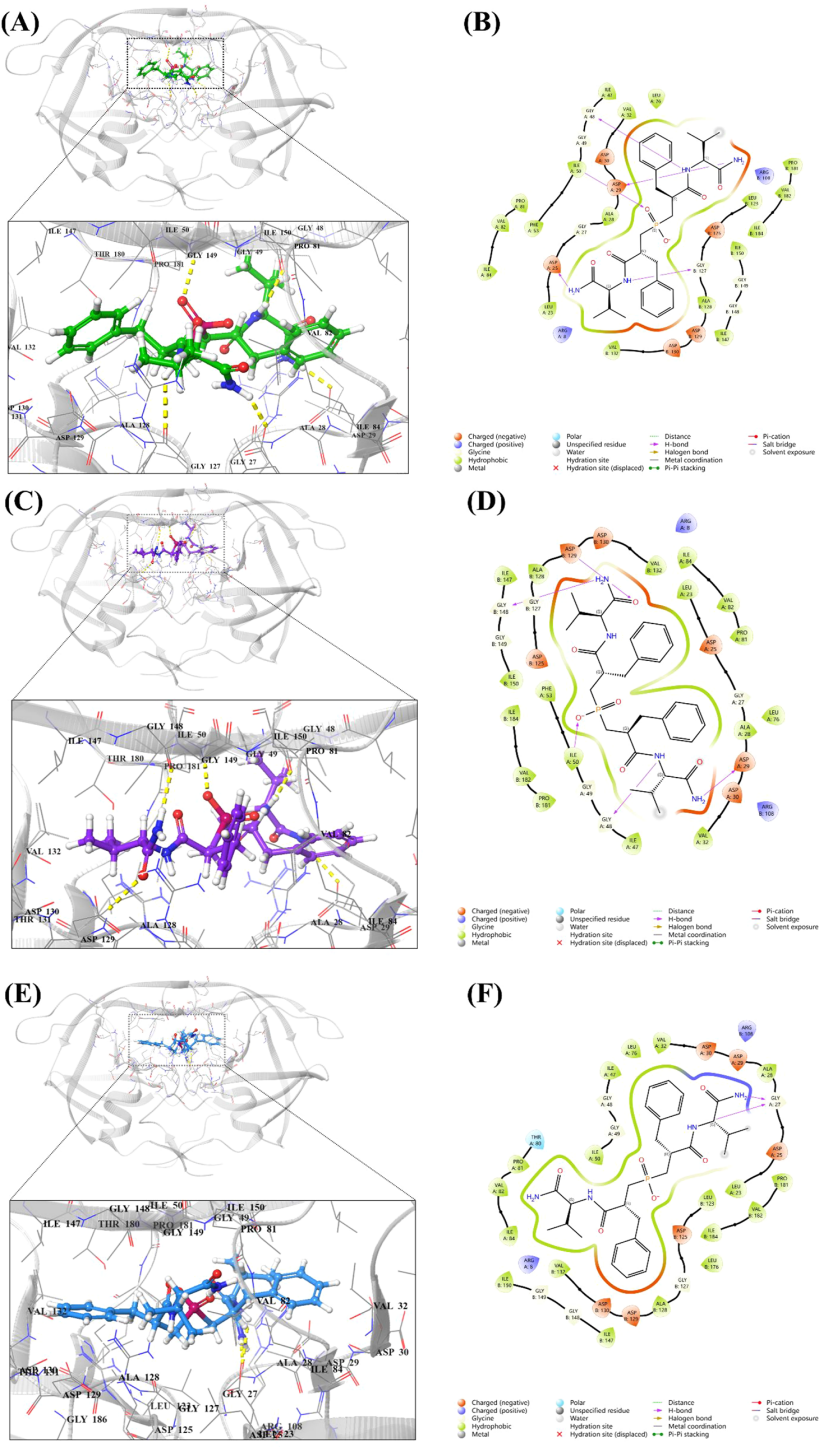
Glide XP-docked poses showing the molecular interactions of (A,-B) *SRSS*, (C, −D) *SSSS*, and (E, -F) *SRRS* with the active pocket of HIV-1 protease (PDB ID: 2IEN). The structure indicated different residual interactions.

The protein-compound interactions for the best AutoDock Vina poses are shown in Figure 9. *SRRS* forms hydrogen bonds mainly with Gly48chainA-Gly148chainB and Arg8chainA-Arg108chainB, with additional interactions involving Asp25chainA-Asp125chainB, Gly27chainA-Gly127chainB, Ala28chainA-Ala128chainB, Ile47chainA-Ile147chainB, Ile50chainA, and Val32chainA, supported by hydrophobic and attractive charge contacts. *SRSS* preserves the hydrogen-bond network with Gly48chainA-Gly148chainB, Arg8chainA-Arg108chainB, and Ile50chainA, while engaging Asp125chainB, Gly27chainA-Gly127chainB, Ala28chainA-Ala128chainB, Ile47chainA-Ile147chainB, Ile50chainA-Ile150chainB, and Val32chainA through comparable hydrophobic and electrostatic contacts. Likewise, *SSSS* relies primarily on hydrogen bonds to Ile50chainA, Arg8chainA-Arg108chainB, and Gly48chainA-Gly148chainB, complemented by interactions with Ala28chainA-Ala128chainB, Ile47chainA-Ile147chainB, Val32chainA, and Asp125chainB. Glide XP and AutoDock Vina results indicate that the two algorithms converge on several conserved contacts, most notably hydrogen bonding to the catalytic Asp25chainA-Asp125chainB dyad and to the flap residues Gly48chainA-Gly148chainB and Ile50chainA. Glide XP predicts that *SRSS* engages deeply within the active site cavity, forming an extensive hydrogen-bond network that includes Asp25chainA, Asp29chainA, Gly127chainB, and the Gly48chainA/Ile50chainA flap hinge, whereas AutoDock Vina favors a pose in which *SRSS* is shifted toward the dimer interface, adding contacts with Arg8chainA-Arg108chainB and Ile150chainB. Conversely, for *SRRS* Glide XP identifies Gly27chainA as the principal hydrogen-bond anchor, while Vina maximizes interactions with the flap tips (Gly48chainA-Gly148chainB) and the *N*-terminal Arg8chainA-Arg108chainB pair. *SSSS* shows the smallest discrepancy that both methods agree on flap engagement, yet Glide XP places greater emphasis on Asp29chainA-Asp129chainB interactions, whereas Vina highlights Val32chainA and Asp125chainB at the pocket floor. Methodological factors likely underlie these discrepancies. Glide XP employs an expanded sampling protocol with proprietary van der Waals radii scaling followed by an explicit-hydration-aware scoring term, which tends to reward deep penetration into polar subpockets such as the Asp29/Gly27 region. AutoDock Vina, by contrast, treats the protein as rigid, uses a simpler empirical scoring function, and can overweight shape complementarity near the protein surface. This bias may explain its preference for flap-tip and Arg8/Arg108 contacts. Protonation states (all Asp/Glu residues were modeled as deprotonated at pH 7.0) and the omission of crystallographic waters further influence electrostatic terms differently in the two programs. In addition, neither protocol captures full protein flexibility or the entropic cost of restraining the highly mobile flaps, and both rely on short-range scoring that can misestimate long-range Coulombic stabilization. Finally, the docking was performed on a single conformation of HIV-1 protease. Future ensemble docking or microsecond-scale molecular dynamics could reveal alternative binding modes or induced-fit rearrangements that reconcile the distinct binding poses predicted separately by Glide XP and AutoDock Vina. Additionally, crystallography and mutagenesis (Gly48, Arg8, Asp29) are required to resolve the preferred binding modes of three isomers.

**Figure 9:**
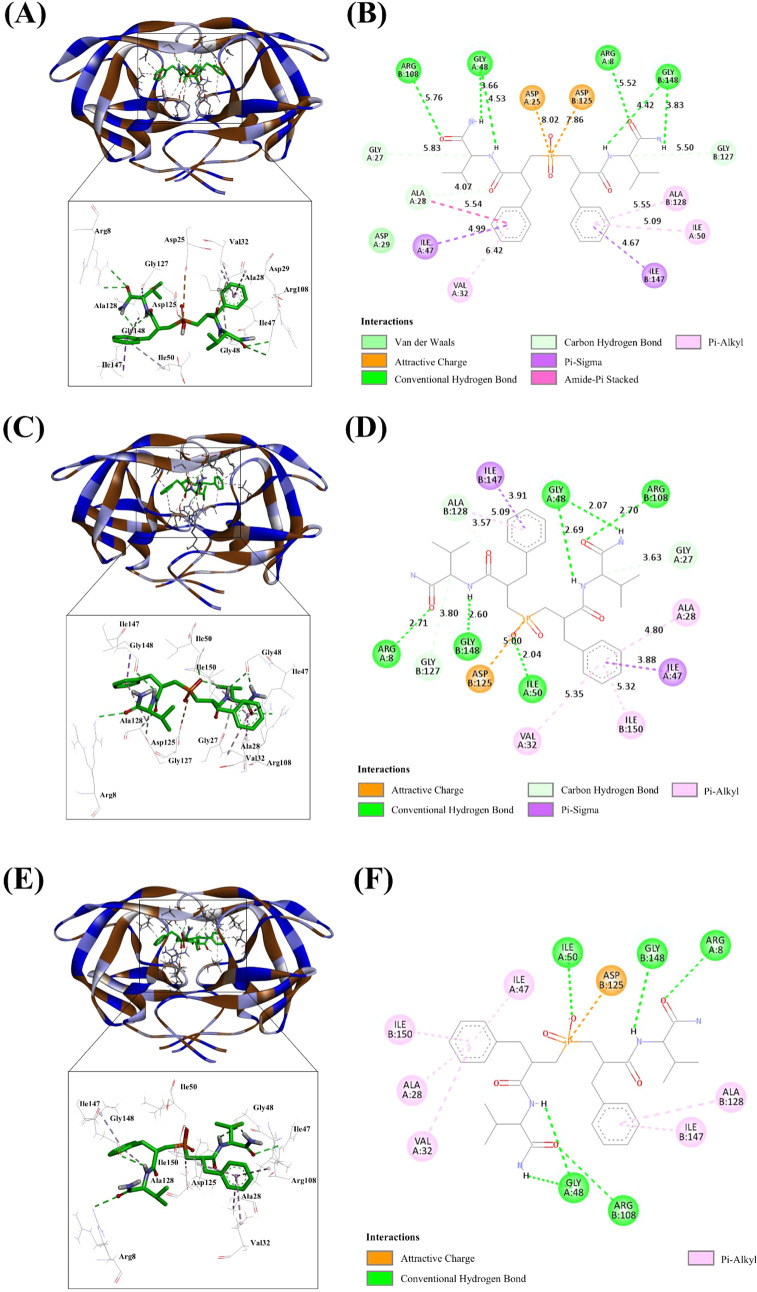
Autodock Vina-docked poses showing the molecular interactions of (A, -B) *SRRS*, (C, −D) *SRSS*, and (E, -F) *SSSS* with the active pocket of HIV-1 protease (PDB ID: 2IEN). The structure indicated different residual interactions.

## Conclusions

The design and synthesis of symmetrical phosphinic pseudopeptides (**PACs**) have proven to be a promising strategy for developing potent inhibitors of HIV-1 protease. By incorporating C2-symmetry and leveraging transition-state mimetics, these inhibitors demonstrated significant potency, particularly one of the isomers of PAC-Phe-Val (**9c**), which exhibited an impressive IC_50_ of 0.92 nM against the wild-type HIV-1 protease. This inhibitor showed binding affinity comparable to that of the clinically used Darunavir (IC_50_ = 2.6 nM), suggesting a similar mechanism of action. The incorporation of various hydrophobic amino acids at the P2 and P2’ positions provided valuable insights into the structure–activity relationship (SAR), with PAC-Phe-Val standing out as a lead compound for further optimization. The study also highlights the success of a simplified, cost-effective synthetic route, overcoming the challenges posed by complex multistep procedures in previous methods. The results of molecular docking studies confirmed that PAC-Phe-Val forms stable and strong interactions with critical active site residues, contributing to its high inhibitory potency. Given the encouraging *in vitro* results, future work will focus on determining precise binding mode of PAC-Phe-Val with HIV-1 protease through X-ray crystallography, assessing quantitative IC_50_ values for all PAC-Phe-Val analogues and resistance profiling against mutant strains to fully establish the resistance profiles, improving the pharmacokinetics and stability of these inhibitors, and evaluating their suitability as effective antiretroviral therapies for the treatment of HIV/AIDS.

## Experimental Section

### In Vitro Evaluation against HIV-1 Inhibitory Activity

#### Methods

The inhibitory effects of the described compounds against HIV-1 wild-type protease were assessed utilizing the fluorometric HIV-1 protease assay kit (SensoLyte 520 HIV Protease Assay Kit AS-71147, AnaSpec Inc., USA), following the manufacturer’s recommended protocol. The FDA-approved HIV-1 protease inhibitor Darunavir was used as a positive control. Additionally, 0.01% (v/v) DMSO was employed as the negative control. Darunavir was purchased from MedChemExpress (HY-17040, New Jersey, USA). The tested compounds **9a**–**g** were dissolved in 100% DMSO and further diluted with the assay buffer provided in the kit to varying concentrations. The fluorescence was measured in kinetic mode at 490 nm (excitation) and 520 nm (emission) for 90 min at 37 °C using the Varioskan LUX multimode microplate reader (Thermo Fisher Scientific Inc. USA). The raw data obtained were subsequently normalized relative to the controls, with 0.01% DMSO set as 0% inhibitory activity. The IC_50_ values were calculated by fitting the data to a four-parameter log­(inhibitor) vs response equation using GraphPad Prism version 9.5.0 (GraphPad Software, San Diego, California, USA).

### Docking Studies of the Most Potent PAC Derivative: PAC-Phe-Val (9c)

#### Methods

Molecular interactions of *SRSS*, *SSSS*, and *SRRS* with HIV-1 protease (PDB ID: 2IEN) were evaluated using the XP scoring function in Glide,^60,61^ and Autodock Vina.^51^ Before the initiation of docking studies, all crystallographic waters, including the conserved flap water (HOH 1068), were removed, and ligand from the crystal structure of HIV-1 protease was utilized to identify the binding pocket. The coordinates were maintained to ensure consistency in the binding site analysis throughout the docking simulations. The compounds were docked into the active site of HIV-1 protease by defining a grid box with a spacing of 1 Å, centered around the coordinates 15.72867 × 22.42704 × 17.165137, with grid box dimensions set to 38 × 38 × 38 Å. The docking algorithm was executed with default parameter values. The best-docked poses for HIV-1 protease-*SRSS*, HIV-1 protease-*SSSS*, and HIV-1 protease-*SRRS* were chosen based on binding affinities, docking scores, binding free energies, and proper orientations of ligands in the active pocket of the protein. Visualization of the docked complexes was conducted using the Maestro program suite from Schrödinger (Maestro, Schrödinger, LLC, New York, NY, 2024).

#### MM/GBSA Free Energy Calculations

The Prime MM-GBSA module of the Maestro program suite from Schrödinger^62^ was employed to calculate the binding free energies of the docked compounds with the protease. Binding free energy changes were determined using molecular mechanics generalized Born surface area (MM/GBSA) calculations, following the formula:

$$\mathrm{MMGBSA}\;{\Delta G}_{\mathrm{Bind}}{=G}_{\mathrm{Complex}}-G_{\mathrm{Receptor}}-G_{\mathrm{Ligand}}$$

The protease–ligand complexes were minimized using local optimization features in Prime Wizard of the Maestro program suite in Schrödinger. The OPLS4 force field was used to determine the binding energy (ΔG-bind) of each ligand,^63^ and ligand strain energy was calculated by placing the ligand in a solution autogenerated using the VSGB 2.0 suit.^64^

### General Experimental Information

All glassware was oven-dried, and all reactions involving air-sensitive materials were carried out under a N_2_ atmosphere. Dry solvents, where needed, were procured by Inert PureSolv Solvent Purification System (Et_2_O, THF, DCM). All other solvents were AR grade and used without further purification unless stated otherwise. Commercially available reagents were purchased from Aladdin, TCI, and Adamas and were used without further purification. All column chromatography purifications were conducted using silica gel (230–400 mesh, Silicycle). Silica G TLC plates (Sorbtech, polyester backed, thickness 200 μM, fluorescence UV254) were used for monitoring the reaction progress. For NMR analysis, all samples were dissolved in CDCl_3_ (D-99.8%, + 0.05% v/v TMS) unless stated otherwise. ^1^H, ^31^P, and ^13^C NMR spectra were recorded on a Bruker Avance III 400 and 600 and processed with MestreNova software. Chemical shifts are reported in ppm, and coupling constants are reported in Hertz (Hz). ^1^H NMR spectra in CDCl_3_ are referenced to tetramethylsilane (TMS) at 0.00 ppm and reported using the format: chemical shift (ppm) [multiplicity (s = singlet, d = doublet, t = triplet, q = quartet, m = multiplet, app = apparent), coupling constant(s) (*J* in Hz), integral]. ^13^C NMR spectra are referenced to CDCl_3_ at 77.0 ppm. ^31^P NMR spectra are referenced to H_3_PO_4_. Analytical HPLC was conducted for compounds **9a**–**g** using Agilent Eclipse XDB-C18, 5 μm, 4.6*250 μm column, with eluent A: H_2_O/MeCN/TFA = 90/10/0.1 and eluent B: H_2_O/MeCN/TFA = 10/90/0.09 at flow rate 1 mL/min, at rt at wavelengths: 220 nm/254 nm. The following gradient was used: t = 0 min (10% B), t = 20 min (25% B), t = 90 min (75% B). Preparative HPLC was used for resolving isomers following the method: Mobile Phase: A: H_2_O (0.1% TFA) B: ACN, Gradient: 20% B for 0.2 min, increase to 90% B within 6.8 min, 90% B for 8 min, back to 20% B within 0.1 min, 20% B for 5 min. Flow Rate: 1.0 mL/min, at 40 °C, Column: Shim-pack PREP-ODS­(H)­KIT 4.6*250 mm, 5 μm. The detailed experimental synthetic procedures for compounds **3** and **4** have been previously described.

#### (2-Benzyl-3-ethoxy-3-oxopropyl)­phosphinic Acid (3e)


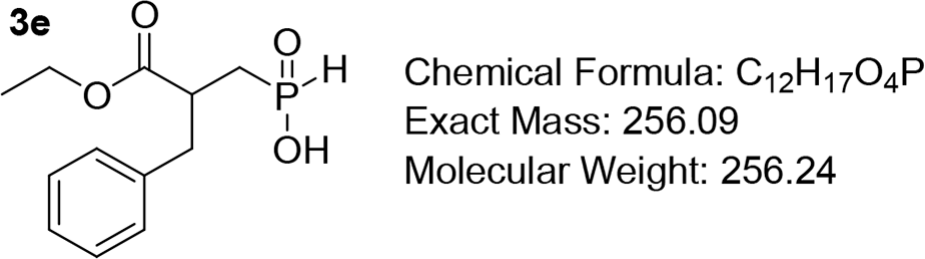


In an oven-dried flask, NH_4_H_2_PO_2_ (4.52 g, 39.25 mmol, 2.5 equiv) and HMDS (13.1 mL, 62.8 mmol, 4 equiv) were heated at 110 °C for 1 h under a continuous flow of N_2_ until a transparent solution was formed. Subsequently, the reaction mixture was cooled to 0 °C, and dry DCM (40 mL) was added. To this cold stirring mixture, a solution of the acrylate **2e**, (15.7 mmol, 1 equiv) in dry DCM (20 mL), was added dropwise over 20 min. The mixture was stirred at rt for 24–48 h. The progress of the reaction was monitored by TLC. Upon completion, MeOH (40 mL) was added slowly at 0 °C, and stirring was continued for 30 min at rt. The volatiles were evaporated under vacuum, and the residue was acidified to pH 1 with 3 M HCl, taken up with DCM, and washed with 1 M HCl. The organic phase was dried over Na_2_SO_4_ and evaporated in vacuo to yield the desired product. Pale-yellow viscous oil; Quantitative yield. R*f* (DCM/MeOH/AcOH = 7/0.8/0.4) = 0.53. ^1^H NMR (400 MHz, CDCl_3_) δ 7.29 (t, *J* = 7.2 Hz, 2H), 7.24 (d, *J* = 7.1 Hz, 1H), 7.18 – 7.15 (m, 2H), 7.08 (d, *J*_=_ 564 Hz, 1H), 4.13 (q, *J* = 7.2 Hz, 2H), 3.10 – 3.04 (m, 2H), 2.91 (dt, *J* = 12.8, 5.2 Hz, 1H), 2.12 (td, *J* = 14.6, 8.8 Hz, 1H), 1.90 – 1.80 (m, 1H), 1.20 (t, *J* = 7.1 Hz, 3H). ^13^C NMR (151 MHz, CDCl_3_) δ 172.9, 136.8, 128.4, 127.8, 126.1, 60.3, 39.9, 38.4, 29.7 (d, *J* = 95.1 Hz), 13.3. ^31^P NMR (243 MHz, CDCl_3_) δ 34.58. MS (ESI) *m*/*z* calcd for C_12_H_16_O_4_P^–^ [M – H]^−^ 255.0, found 255.0.

#### Bis­(2-benzyl-3-ethoxy-3-oxopropyl)­phosphinic Acid (4e)


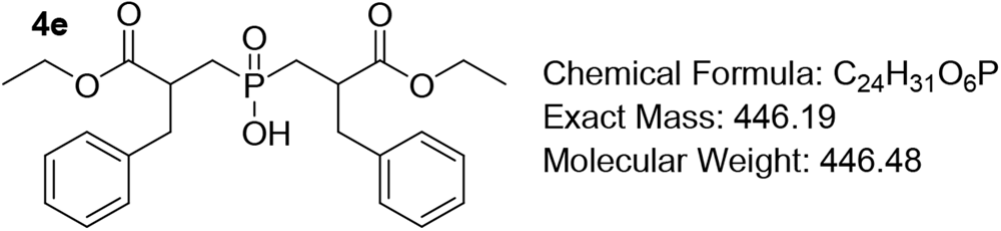


In an oven-dried flask, the phosphinic acid **3e** (1.0 equiv) in dry DCM (2 mL for 1 mmol of the phosphinic acid) was added, followed by the addition of Et_3_N (7.0 equiv) and acrylate **2e** (1.2 equiv). The mixture was cooled to 0 °C and purged with N_2_ for 15 min. Subsequently, TMSCl (7.0 equiv) was added to the reaction mixture dropwise over 15 min. The temperature was slowly raised to rt, and the clear solution was stirred for 48–72 h (or until completion, monitored by ^31^P NMR). MeOH (1 mL for 1 mmol) was added dropwise, and stirring was continued at rt for 30 min. Removal of volatiles under vacuum afforded the crude product as a pale-yellow viscous oil. The crude product was dissolved in EtOAc, and the resulting solution was washed with 1 M HCl (three times) and brine, dried over Na_2_SO_4_, and concentrated in vacuo. The residue was purified by column chromatography, using DCM/MeOH/AcOH 7/0.5/0.1–0.5 as the eluent. Pale-yellow viscous oil (92%); R*f* (DCM/MeOH/AcOH = 7/0.5/0.5) = 0.82. ^1^H NMR (600 MHz, CDCl_3_) δ 7.29 – 7.22 (m, 4H), 7.22 – 7.17 (m, 2H), 7.17 – 7.09 (m, 4H), 4.08 – 3.96 (m, 4H), 3.05 (s, 2H), 2.94 (app q, *J* = 10.5 Hz, 4H), 2.18 – 2.11 (m, 2H), 1.81 (td, *J* = 13.9, 6.5 Hz, 2H), 1.10 (td, *J* = 7.2, 2.7 Hz, 6H). ^13^C NMR (101 MHz, CDCl_3_) δ 174.1 (d, *J* = 6.0 Hz), 137.9, 129.2, 128.4, 126.7, 60.8, 41.0 (d, *J* = 7.0 Hz), 39.7 (d, *J* = 11.1 Hz), 31.1 (d, *J* = 93.9 Hz), 14.0. ^31^P NMR (162 MHz, CDCl_3_) δ 56.28, 56.19. HRMS (ESI) *m*/*z* calcd for C_24_H_31_O_6_PNa [M + Na]^+^ 469.1756, found 469.1746.

#### Ethyl 3-((Adamantan-1-yloxy)­(2-benzyl-3-ethoxy-3-oxopropyl)­phosphoryl)-2-benzylpropanoate (5)


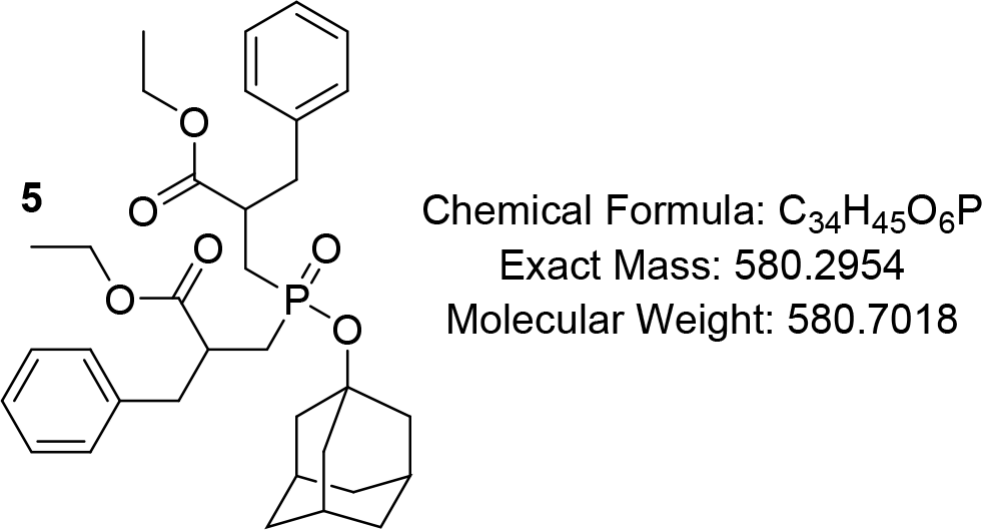


Compound **4e** (4.465 g, 10 mmol) and 1-adamantyl bromide (1.2 equiv, 12 mmol, 2.58 g) were dissolved in CHCl_3_ (100 mL), and the solution was refluxed for 15 min. Silver oxide (1.2 equiv, 12 mmol, 2.78 g) was added to the refluxing solution in five equal portions over 50 min. After the solution was refluxed for 5 h, or until completion, the solvent was removed under reduced pressure, and the residue was suspended in diethyl ether. The silver bromide was removed by filtration through Celite. The filtrates were concentrated under reduced pressure and further purified chromatographically using DCM/i-PrOH = 9.8/0.2 as eluent to provide compound **5**.

White solid (92%); R*f* (DCM/MeOH = 9.7/0.3) 0.72. ^1^H NMR (600 MHz, CDCl_3_) δ 7.21 – 7.17 (m, 4H), 7.12 (app t, *J* = 7.3 Hz, 2H), 7.08 (d, *J* = 7.6 Hz, 4H), 4.03 – 3.90 (m, 4H), 2.95 – 2.85 (m, 4H), 2.81 – 2.73 (m, 2H), 2.17 – 2.04 (m, 2H), 2.04 – 1.98 (m, 4H), 1.86 – 1.78 (m, 6H), 1.69 (tt, *J* = 14.0, 3.8 Hz, 1H), 1.50 – 1.46 (m, 6H), 1.07 – 1.01 (m, 6H); ^13^C NMR (101 MHz, CDCl_3_) δ 174.4, 174.3, 138.2, 129.3, 129.2, 128.5, 128.4, 126.7, 126.6, 64.4, 60.7, 49.3, 45.9, 44.1, 41.8, 41.7, 36.5, 35.7, 35.5, 32.6, 31.7, 31.1, 25.4, 14.0; ^31^P NMR (162 MHz, CDCl_3_) δ 48.88, 48.64, 48.42.

#### 3-((Adamantan-1-yloxy)­(2-carboxy-3-phenylpropyl)­phosphoryl)-2-benzylpropanoic Acid (6)


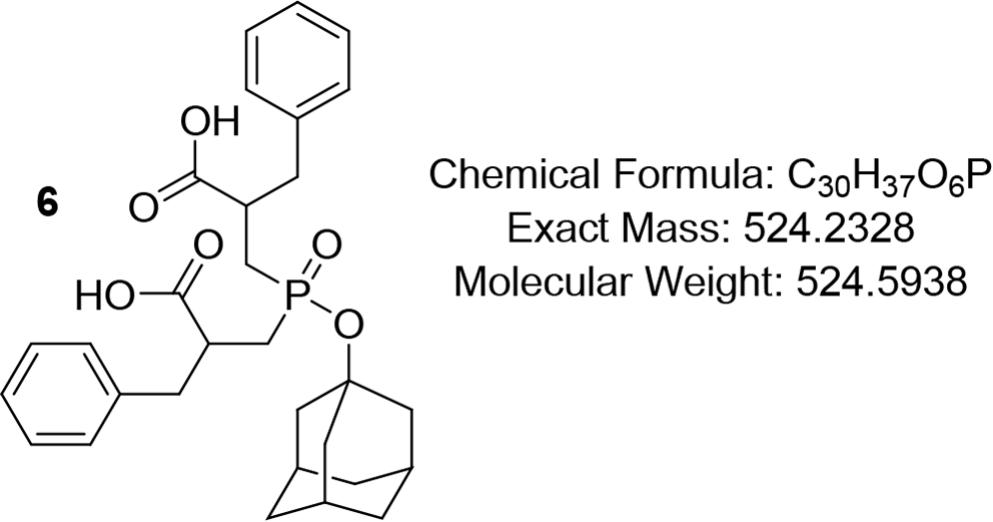


In a solution of compound **5** (13 g, 22.39 mmol) dissolved in 18.6 mL MeOH, 6 M LiOH aqueous solution (18.6 mL) was added dropwise; the final concentration of the LiOH was 3 M. The mixture was stirred for 24 h at rt. The solvent was removed under reduced pressure, and the residue was diluted with water and acidified with 0.5 M HCl to pH 1 in an ice–water bath. The aqueous solution was extracted 3 times with AcOEt, and the organic layer was washed with water and brine, then dried with Na_2_SO_4_, filtered, and concentrated under reduced pressure to provide. Column chromatography using DCM/MeOH = 9.5/0.5 provided compound **6**. White solid (85%); R*f* (DCM/MeOH = 9.5/0.5) 0.35. ^1^H NMR (400 MHz, CDCl_3_) δ 7.34 – 7.12 (m, 10H), 3.3 – 3.0 (m, 3H), 2.92 (s, 1H), 2.79 – 2.58 (m, 2H), 2.37 – 2.14 (m, 3H), 2.10 – 1.96 (m, 3H), 1.91 (s, 1H), 1.76 (app d, *J* = 13.3 Hz, 5H), 1.65 (app s, 3H), 1.59 – 1.42 (m, 6H); ^13^C NMR (101 MHz, CDCl_3_) δ 177.3, 138.2, 138.1, 129.3, 129.2, 128.6, 128.5, 126.7, 45.2, 44.0, 36.1, 35.5, 31.1, 30.7; ^31^P NMR (162 MHz, CDCl_3_) δ 54.26, 53.21, 52.10.

### General Procedure for the Synthesis of Compounds 8a–g

To a stirring suspension of compound **6** (1.4 mmol, 0.73 g) in DCM (35 mL) DIPEA (4 equiv, 5.6 mmol, 1.44 g, 0.95 mL) was added, then the amide **7a**–**g** (2.2 equiv, 3.1 mmol), and HOBt (2 equiv, 2.8 mmol, 0.37 g), followed by the addition of EDC·HCl (5.7 equiv, 8 mmol, 2.13 g) and another 5.6 mmol (4 equiv) of DIPEA. The reaction mixture was stirred at rt for 12–24 h. On completion, as monitored by TLC, the reaction mixture was diluted with DCM (60 mL) and washed successively with 1 M HCl (3 × 5 mL), saturated NH_4_HCO_3_ solution (3 × 1 mL), 1 M HCl (to pH 1), and brine (10 mL). The organic layer was dried over Na_2_SO_4_, concentrated under reduced pressure, and the products were purified by column chromatography using DCM/MeOH = 9.8/0.2 to 9/1 as eluent to afford the target products.

#### Adamantan-1-yl Bis­(3-((2-amino-2-oxoethyl)­amino)-2-benzyl-3-oxopropyl)­phosphinate (8a)


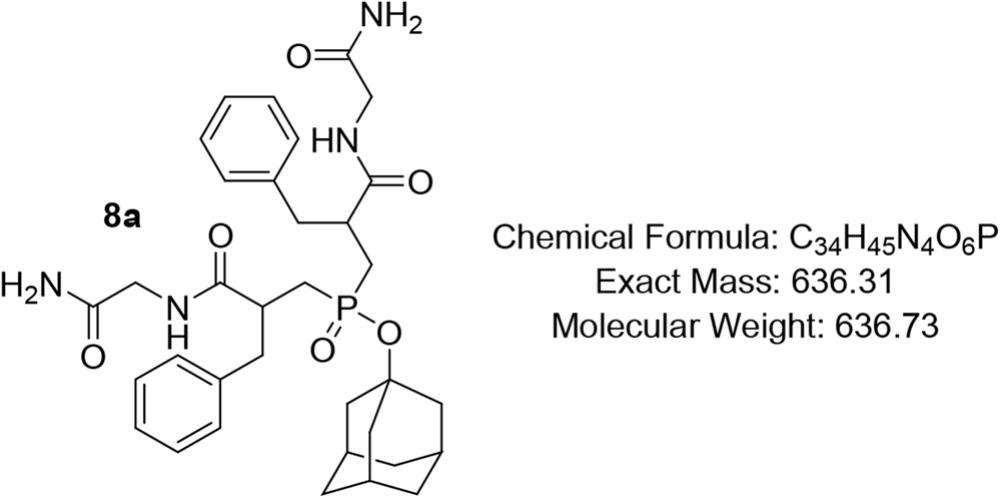


Pale-yellow oil (76%); R*f* (DCM/MeOH = 9/1) 0.37. ^1^H NMR (600 MHz, CDCl_3_) δ 8.18 (s, 1H), 7.84 (s, 1H), 7.47 (s, 1H), 7.25 – 7.19 (m, 4H), 7.18 – 7.08 (m, 6H), 6.95 (s, 1H), 6.73 (s, 1H), 6.46 (s, 1H), 4.19 – 3.87 (m, 2H), 3.68 (s, 1H), 3.55 – 3.15 (m, 2H), 3.06 – 2.79 (m, 4H), 2.61 – 2.42 (m, 2H), 2.14 (s, 2H), 1.97 (*app* d, *J* = 33.4 Hz, 3H), 1.86 (s, 1H), 1.65 – 1.59 (m, 5H), 1.50 – 1.36 (m, 7H); ^13^C NMR (101 MHz, CDCl_3_) δ 174.6 (d, *J* = 18.7 Hz), 174.5, 138.4, 138.2, 129.2, 128.6, 126.7, 50.3, 44.4, 44.3, 44.2, 43.0, 40.2, 35.6, 35.5, 31.1, 31.0; ^31^P NMR (162 MHz, CDCl_3_) δ 51.86, 50.82, 49.50.

#### Adamantan-1-yl Bis­(3-(((S)-1-amino-1-oxopropan-2-yl)­amino)-2-benzyl-3-oxopropyl)­phosphinate (8b)


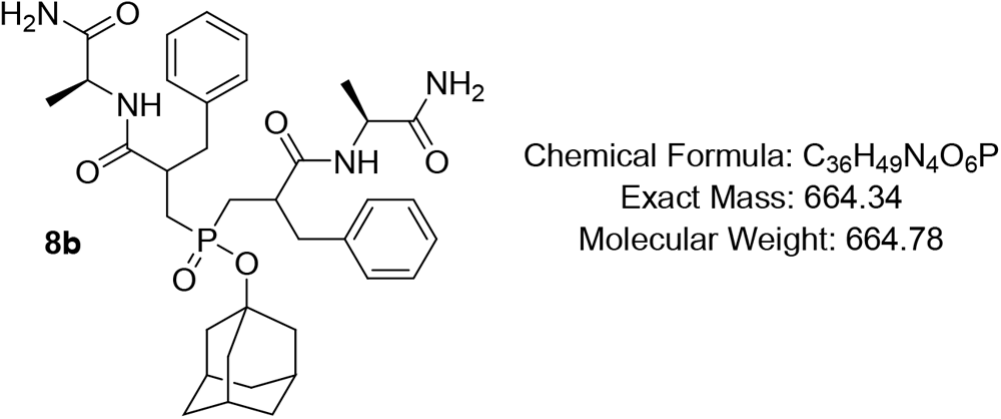


Pale-yellow oil, (83%); R*f* (DCM/MeOH = 9/1) 0.40, 0.46. ^1^H NMR (400 MHz, CDCl_3_) δ 8.10 – 7.76 (m, 1H), 7.67 – 7.40 (m, 2H), 7.33 – 7.09 (m, 10H), 7.00 – 6.69 (m, 2H), 6.45 – 6.15 (m, 1H), 4.43 – 4.23 (m, 2H), 2.80 – 2.65 (m, 4H), 2.82 – 2.63 (m, 2H), 2.52 – 2.34 (m, 1H), 2.24 – 2.14 (m, 1H), 2.08 (*app* d, *J* = 10.4 Hz, 3H), 1.93 (*app* s, 1H), 1.87 – 1.70 (m, 6H), 1.64 – 1.46 (m, 7H), 1.34 – 1.26 (m, 3H), 1.14 (*app* q, *J* = 7.7 Hz, 3H); ^13^C NMR (101 MHz, CDCl_3_) δ 176.4, 176.1, 175.8, 175.5 (d, *J* = 5.8 Hz), 175.4 (d, *J* = 4.0 Hz), 174.6, 174.0, 173.6, 173.3, 138.5, 138.4, 138.3, 129.3, 129.2, 129.1, 128.7, 128.6, 128.5, 126.7, 82.9, 82.8, 82.7, 82.4 (d, *J* = 10.0 Hz), 50.5, 49.3, 49.2, 48.9, 48.8, 48.7, 48.5, 45.4, 44.4, 44.3, 44.2, 42.6, 42.2, 40.6, 36.1, 35.6, 35.5, 31.1, 31.0, 17.5, 17.3 (d, *J* = 4.3 Hz), 17.20 (d, *J* = 3.0 Hz), 16.9; ^31^P NMR (162 MHz, CDCl_3_) δ 51.38, 50.89, 50.67, 50.22.

#### Adamantan-1-yl Bis­(3-(((S)-1-amino-3-methyl-1-oxobutan-2-yl)­amino)-2-benzyl-3-oxopropyl)­phosphinate (8c)


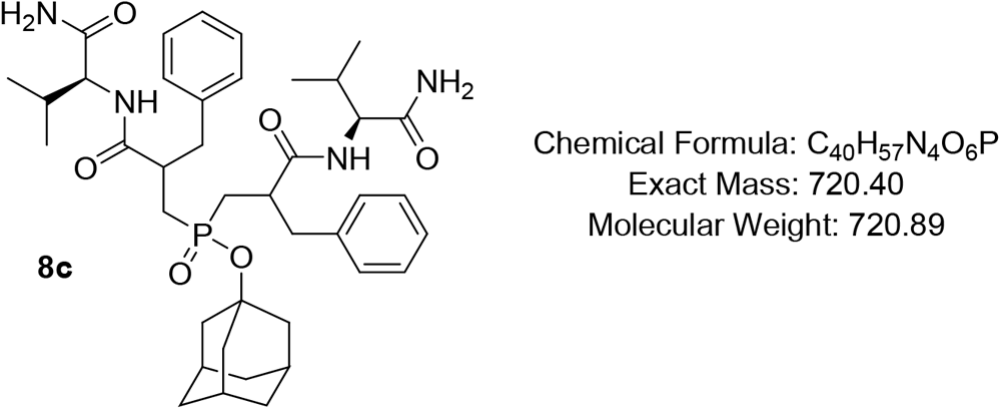


Pale-yellow oil (82%); R*f* (DCM/MeOH = 9/1) 0.51, 0.54. ^1^H NMR (600 MHz, CDCl_3_) δ 7.78 (s, 1H), 7.35 (t, *J* = 7.6 Hz, 1H), 7.29 – 7.09 (m, 11H), 7.00 (d, *J* = 7.2 Hz, 1H), 6.87 – 6.41 (m, 2H), 4.41 – 4.17 (m, 2H), 3.23 – 2.92 (m, 4H), 2.79 – 2.56 (m, 2H), 2.50 – 2.25 (m, 1H), 2.13 (*app* q, *J* = 6.8 Hz, 2H), 2.09 – 2.03 (m, 3H), 1.92 (*app* d, *J* = 11.1 Hz, 1H), 1.88 – 1.74 (m, 6H), 1.67 – 1.45 (m, 7H), 0.98 – 0.84 (m, 6H), 0.84 – 0.72 (m, 5H), 0.69 (d, *J* = 7.0 Hz, 1H), 0.59 (d, *J* = 6.9 Hz, 1H; ^13^C NMR (101 MHz, CDCl_3_) δ 175.1, 174.5, 174.1, 138.6, 138.4, 129.3, 129.2, 129.1, 128.8, 128.6, 128.5, 126.7, 126.6, 44.2, 35.6 (d, *J* = 6.2 Hz), 31. One (d, *J* = 6.9 Hz), 19.4, 19.2, 19.1 (d, *J* = 3.4 Hz); ^31^P NMR (162 MHz, CDCl_3_) δ 51.42, 51.32, 51.07, 50.61.

#### Adamantan-1-yl Bis­(3-(((S)-1-amino-4-methyl-1-oxopentan-2-yl)­amino)-2-benzyl-3-oxopropyl)­phosphinate (8d)


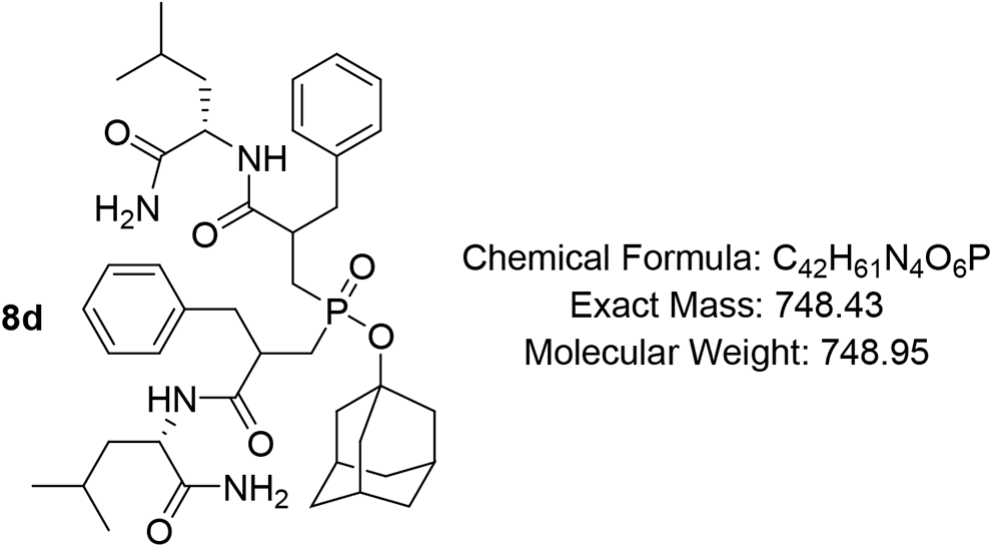


Pale-yellow oil, (85%), R*f (*DCM/MeOH = 9/1) 0.45, 0.58. ^1^H NMR (400 MHz, CDCl_3_) δ 8.10 – 7.87 (m, 1H), 7.68 – 7.54 (m, 1H), 7.37 – 7.14 (m, 10H), 7.09 (d, *J* = 7.5 Hz, 1H), 7.01 – 6.80 (m, 1H), 6.76 – 6.56 (m, 1H), 6.41 – 6.22 (m, 1H), 4.48 – 4.26 (m, 2H), 3.22 – 2.91 (m, 4H), 2.80 – 2.63 (m, 2H), 2.45 – 2.34 (m, 1H), 2.28 – 2.16 (m, 1H), 2.12 – 2.04 (m, 3H), 1.99 – 1.85 (m, 3H), 1.85 – 1.72 (m, 4H), 1.55 (d, *J* = 10.9 Hz, 11H), 1.42 – 1.20 (m, 2H), 0.92 – 0.82 (m, 8H), 0.76 (dt, *J* = 19.4, 6.0 Hz, 3H), 0.67 (d, *J* = 6.4 Hz, 1H); ^13^C NMR (101 MHz, CDCl_3_) δ 176.3, 175.9, 175.7, 174.2, 173.8, 173.7, 138.7 (d, *J* = 4.8 Hz), 138.3 (d, *J* = 4.8 Hz), 129.4, 129.3, 129.2, 129.1, 128.7, 128.6, 128.5, 126.7 (d, *J* = 6.7 Hz), 126.6, 44.3, 35.6, 31.1 (d, *J* = 2.9 Hz), 31.0 (d, *J* = 4.1 Hz), 24.7, 23.5 (d, *J* = 2.1 Hz), 21.8, 21.1 (d, *J* = 6.9 Hz); ^31^P NMR (162 MHz, CDCl_3_) δ 51.67, 51.57, 50.96, 50.71.

#### Adamantan-1-yl Bis­(3-(((S)-1-amino-1-oxo-3-phenylpropan-2-yl)­amino)-2-benzyl-3-oxopropyl)­phosphinate (8e)


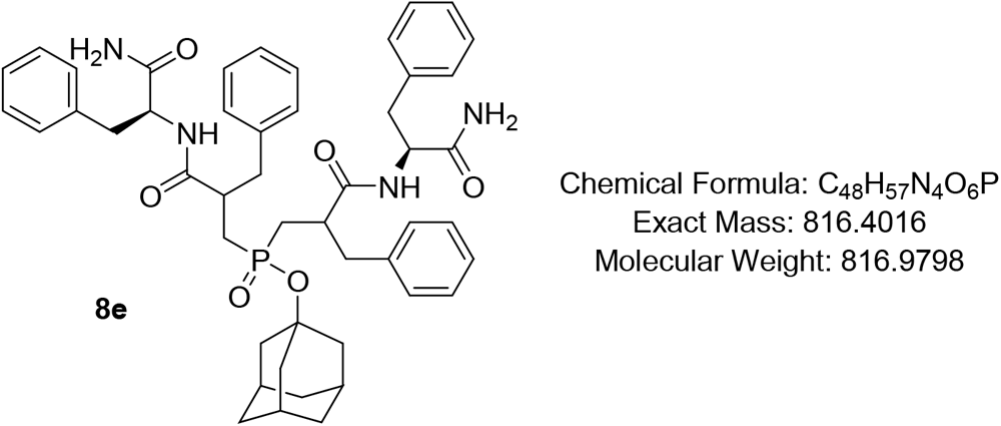


Pale-yellow oil (79%); R*f* (DCM/MeOH = 9/1) 0.68, 0.72. ^1^H NMR (600 MHz, CDCl_3_) δ 7.93 – 7.65 (m, 1H), 7.60 – 7.39 (m, 1H), 7.32 – 6.99 (m, 20H), 6.96 – 6.80 (m, 2H), 6.77 – 6.39 (m, 2H), 4.79 – 4.67 (m, 1H), 4.64 – 4.47 (m, 1H), 3.42 – 3.36 (m, 1H), 3.32 – 3.25 (m, 1H), 3.20 – 3.07 (m, 2H), 3.03 – 2.96 (m, 1H), 2.95 – 2.88 (m, 1H), 2.88 – 2.75 (m, 3H), 2.68 (app d, *J* = 6.8 Hz, 1H), 2.64 – 2.56 (m, 1H), 2.55 – 2.44 (m, 1H), 2.41 – 2.31 (m, 1H), 2.26 – 2.10 (m, 1H), 2.10 – 2.00 (m, 2H), 1.96 (*app* d, *J* = 20.1 Hz, 1H), 1.90 – 1.80 (m, 3H), 1.70 – 1.57 (m, 3H), 1.57 – 1.45 (m, 4H), 1.41 (t, *J* = 14.2 Hz, 1H), 1.34 – 1.24 (m, 1H); ^13^C NMR (101 MHz, CDCl_3_) δ 175.1, 174.4, 173.9, 173.8, 138.5, 137.7, 129.5, 129.4 (d, *J* = 2.8 Hz), 129.3 (d, *J* = 4.5 Hz), 129.2 (d, *J* = 2.6 Hz), 129.1, 128.9, 128.7 (d, *J* = 3.9 Hz), 128.6, 128.5, 128.4, 127.0, 126.9, 126.8, 126.7, 126.6, 50.4, 45.3, 44.3, 44.2, (d, *J* = 3.3 Hz), 36.1, 35.6, 35.4, 31.1 (d, *J* = 3.0 Hz), 31.0; ^31^P NMR (162 MHz, CDCl_3_) δ 51.51, 51.35, 51.00, 50.53.

#### Adamantan-1-yl Bis­(2-benzyl-3-((S)-2-carbamoylpyrrolidin-1-yl)-3-oxopropyl)­phosphinate (8f)


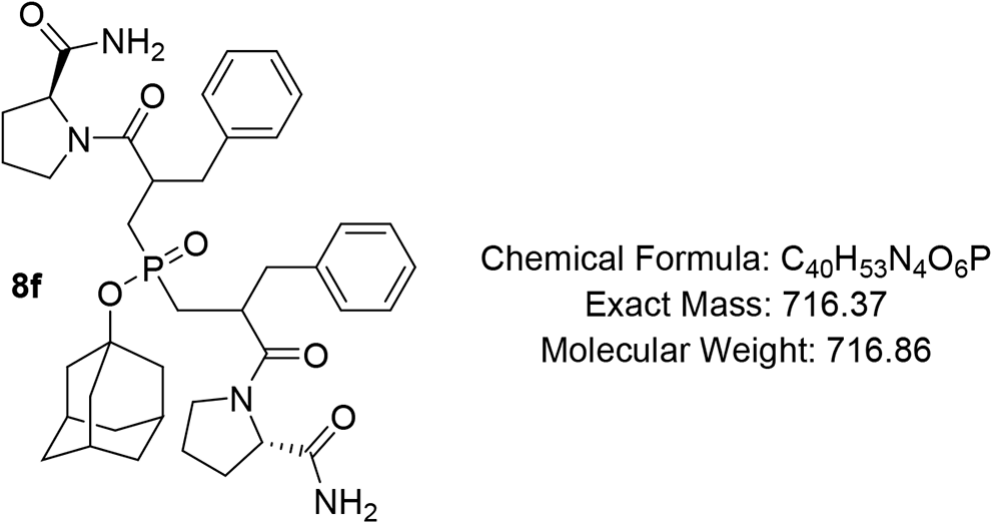


Pale-yellow oil (68%); R*f* (DCM/MeOH = 9/1) 0.72, 0.75. ^1^H NMR (400 MHz, CDCl_3_) δ 7.89 – 7.63 (m, 1H), 7.50 – 7.42 (m, 1H), 7.28 – 7.10 (m, 10H), 6.53 – 6.13 (m, 1H), 5.89 – 5.59 (m, 1H), 4.58 – 4.27 (m, 2H), 3.67 – 3.47 (m, 2H), 3.46 – 3.26 (m, 2H), 3.19 – 3.01 (m, 2H), 2.94 – 2.82 (m, 2H), 2.80 – 2.73 (m, 1H), 2.71 – 2.54 (m, 3H), 2.51 – 2.39 (m, 1H), 2.19 – 2.01 (m, 6H), 1.98 – 1.91 (m, 2H), 1.90 – 1.82 (m, 3H), 1.80 – 1.76 (m, 2H), 1.73 – 1.38 (m, 11H); ^13^C NMR (101 MHz, CDCl_3_) δ 175.1, 175.0, 174.3, 173.7 (d, *J* = 2.6 Hz), 173.1, 172.9, 172.8, 172.6, 138.3 (d, *J* = 2.7 Hz), 138.0, 137.8, 129.3, 129.2, 129.1, 129.0, 128.8, 128.7, 128.6, 128.5 (d, *J* = 2.0 Hz), 128.4 (d, *J* = 2.6 Hz), 127.1, 127.0, 126.9, 83.2, 60.4, 60.2, 59.9, 59.7, 47.3, 46.8, 46.6 (d, *J* = 9.1 Hz), 44.5, 44.3 (d, *J* = 3.7 Hz), 40.8, 35.6, 31.0, 29.3 (d, *J* = 7.8 Hz), 29.1, 27.8, 24.7 (d, *J* = 5.8 Hz), 23.8; ^31^P NMR (162 MHz, CDCl_3_) δ 57.40, 56.59, 55.99, 55.26, 51.33, 50.72, 50.59, 50.25, 50.07, 49.72, 49.45, 49.17, 49.02.

#### Adamantan-1-yl Bis­(3-(((S)-1-amino-3-(1H-indol-3-yl)-1-oxopropan-2-yl)­amino)-2-benzyl-3-oxopropyl)­phosphinate (8g)


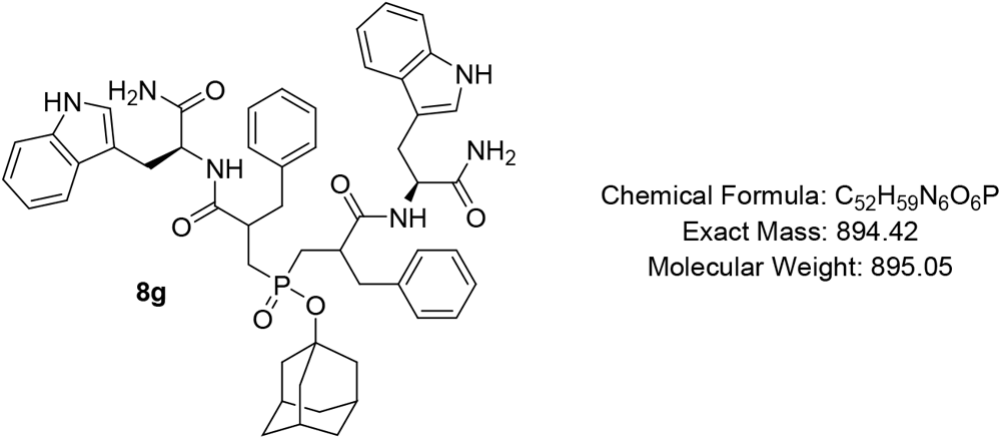


Pale-yellow oil (66%); R*f* (DCM/MeOH = 9/1) 0.42. ^1^H NMR (600 MHz, CDCl_3_) δ 9.43 – 8.69 (m, 2H), 7.56 – 7.52 (m, 1H), 7.33 – 6.95 (m, 20H), 6.82 – 6.68 (m, 4H), 6.32 (s, 1H), 4.71 (app s, 1H), 4.55 (app s, 1H), 3.27 – 3.07 (m, 3H), 2.93 (app s, 1H), 2.79 (app s, 2H), 2.61 – 2.31 (m, 5H), 1.94–1.93 (m, 3H), 1.81 – 1.70 (m, 5H), 1.45 – 1.34 (m, 8H), 1.27 (t, *J* = 9.5 Hz, 2H); ^13^C NMR (101 MHz, CDCl_3_) δ 175.3, 174.9, 173.9, 173.7, 138.6, 138.2, 136.2 (d, *J* = 19.0 Hz), 129.2 (d, *J* = 7.7 Hz), 128.7, 127.7 (d, *J* = 4.4 Hz), 127.5, 126.8, 124.3, 121.9, 119.3, 118.6, 111.6, 110.1, 50.5, 44.2, 44.1, 44.0 (d, *J* = 3.7 Hz), 43.9 (d, *J* = 3.6 Hz), 35.5, 35.4, 31.0 (d, *J* = 3.5 Hz), 30.9 (d, *J* = 2.2 Hz); ^31^P NMR (162 MHz, CDCl_3_) δ 51.25, 50.41, 50.11.

### General Procedure for the Synthesis of Compounds 9a–g

The *P*-Ad protected phosphinopeptide derivatives **8a**–**g** (1.5 mmol) were treated with a mixture of 10 mL DCM/TFA/TIS/H_2_O = 50/50/0.1/0.1 and stirred for 3 h at rt. The solvents were removed under reduced pressure upon completion of the reaction, as monitored by TLC. The products were dissolved in the minimum amount of DCM, and a mixture of Et_2_O/hexanes = 1/1 was added to precipitate the products, which were filtered and washed with Et_2_O/hexanes = 1/1.

#### Bis­(3-((2-amino-2-oxoethyl)­amino)-2-benzyl-3-oxopropyl)­phosphinic Acid (PAC-Phe-Gly) (9a)


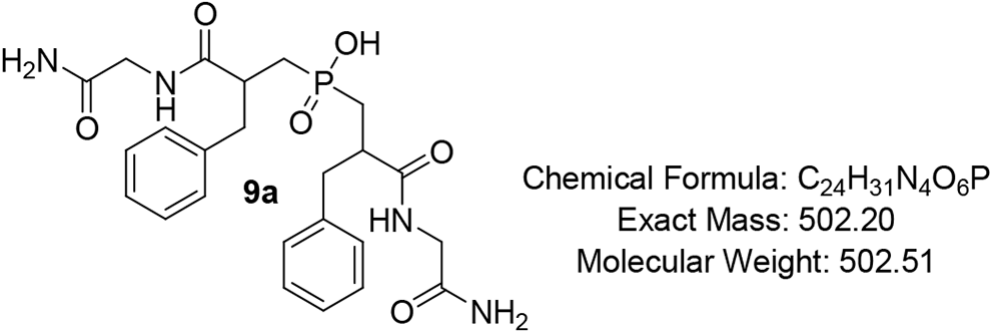


Pale-yellow oil (86%); R*f* (DCM/MeOH/AcOH = 7/0.5/0.5) 0.16. ^1^H NMR (600 MHz, DMSO) δ 8.34 – 8.24 (m, 2H), 7.56 (app d, *J* = 5.4 Hz, 1H), 7.28 – 7.24 (m, 5H), 7.20 – 7.14 (m, 7H), 6.98 – 6.92 (m, 1H), 3.73 – 3.67 (m, 2H), 3.33 – 3.25 (m, 1H), 2.89 – 2.80 (m, 4H), 2.65 – 2.59 (m, 2H), 2.21 – 2.19 (m, 2H), 1.99 – 1.95 (m, 2H), 1.89 (t, *J* = 3.6 Hz, 2H); ^13^C NMR (151 MHz, DMSO) δ 174.1 (d, *J* = 3.4 Hz), 174.0 (d, *J* = 3.2 Hz), 172.1, 172.0, 139.5, 139.4, 129.5, 128.6, 126.7, 87.9, 66.4, 45.7, 42.7, 40.7, 36.4, 35.6, 30.9, 30.5, 29.3, 18.3, 12.5; ^31^P NMR (243 MHz, DMSO) δ 46.60, 46.56; MS (ESI) *m*/*z* calcd. for C_24_H_30_N_4_O_6_P^–^ [M—H]^−^ = 501.1, found 501.1; HRMS (ESI) *m*/*z* calcd. for C_24_H_31_N_4_O_6_P [M + H]^+^ 503.2053, found 503.2045; HPLC: DAD1A/B, Sig = 220/254: rt 12.374, 14.780, purity = 80%

#### Bis­(3-(((S)-1-amino-1-oxopropan-2-yl)­amino)-2-benzyl-3-oxopropyl)­phosphinic Acid (PAC-Phe-Ala) (9b)


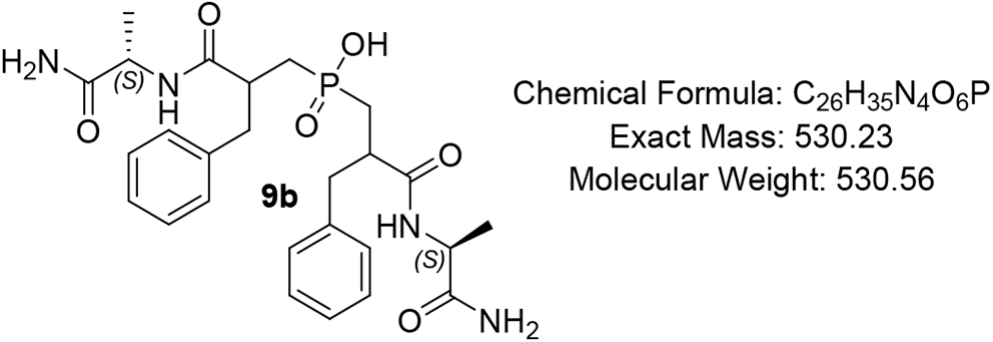


Pale-yellow oil (84%); R*f* (DCM/MeOH/AcOH = 7/0.5/0.5) 0.17. ^1^H NMR (600 MHz, DMSO-*d*_6_) δ 8.11 (d, *J* = 7.6 Hz, 1H), 8.03 – 7.96 (m, 2H), 7.83 (d, *J* = 5.4 Hz, 1H), 7.56 (s, 1H), 7.30 – 7.22 (m, 4H), 7.21 – 7.11 (m, 6H), 6.92 – 6.85 (m, 1H), 4.16 – 3.97 (m, 2H), 2.90 – 2.80 (m, 3H), 2.77 – 2.67 (m, 2H), 2.60 – 2.60 (m, 1H), 1.93 – 1.85 (m, 1H), 1.23 (app s, 2H), 1.21 – 1.15 (m, 1H), 1.71 – 1.13 (m, 1H), 0.97 – 0.86 (m, 6H); ^13^C NMR (151 MHz, DMSO) δ 173.4, 173.3, 172.9 (d, *J* = 2.3 Hz), 171.5, 127.7, 126.7, 86.1, 64.7, 43.9, 38.9, 34.6, 33.8, 29.2, 28.8, 16.5, 10.8; ^31^P NMR (243 MHz, DMSO) δ 46.96, 46.89, 46.81; MS (ESI) *m*/*z* calcd. for C_26_H_34_N_4_O_6_P^–^ [M—H]^−^ = 529.3, found 529.3; HRMS (ESI) *m*/*z* calcd. for C_26_H_35_N_4_O_6_P [M + H]^+^ 531.2366 found 531.2369; HPLC: DAD1A/B, Sig = 220/254: rt 12.070, 14.255, 21.342, purity = 90%

#### Bis­(3-(((S)-1-amino-3-methyl-1-oxobutan-2-yl)­amino)-2-benzyl-3-oxopropyl)­phosphinic Acid (9c)


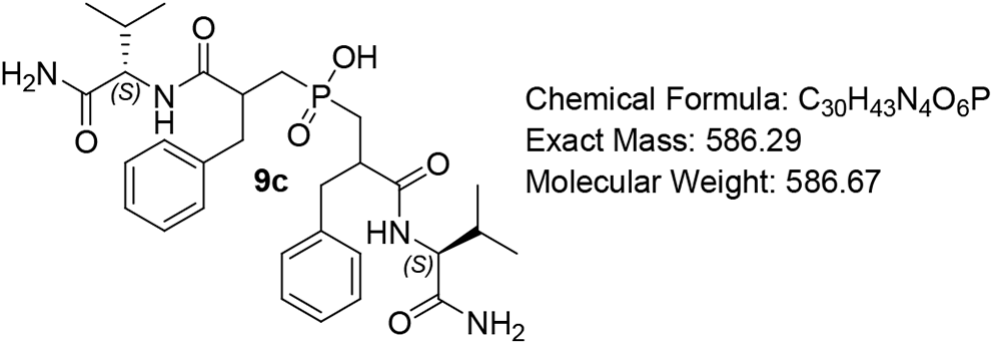


White solid (82%); R*f* (DCM/MeOH/AcOH = 7/0.5/0.5) 0.20. ^1^H NMR (600 MHz, DMSO-*d*_6_) δ 8.10 (d, *J* = 8.9 Hz, 1H), 8.00 – 7.87 (m, 1H), 7.75 – 7.68 (m, 1H), 7.28 – 7.13 (m, 11H), 6.97 – 6.90 (m, 1H), 6.78 (s, 1H), 4.06 (ddd, *J* = 26.0, 8.9, 6.5 Hz, 1H), 3.92 (ddd, *J* = 45.4, 8.7, 4.5 Hz, 1H), 3.22 (app d, *J* = 11.3 Hz, 1H), 3.07 – 3.01 (m, 1H), 2.96 – 2.86 (m, 1H), 2.78 – 2.67 (m, 3H), 2.04 – 1.94 (m, 3H), 1.92 – 1.86 (m, 1H), 1.59 – 1.53 (m, 3H), 0.99 (d, *J* = 7.0 Hz, 1H), 0.85 (ddd, *J* = 19.2, 6.8, 3.7 Hz, 5H), 0.64 (dd, *J* = 21.9, 6.9 Hz, 3H), 0.49 (d, *J* = 6.8 Hz, 1H), 0.36 (d, *J* = 6.8 Hz, 1H); ^13^C NMR (151 MHz, DMSO) δ 173.9, 173.8, 173.5 (d, *J* = 6.8 Hz), 139.7 (d, *J* = 20.5 Hz), 139.4, 126.5 (d, *J* = 4.1 Hz), 129.5, 128.6, 128.5, 126.5, 58.3 (d, *J* = 11.5 Hz), 57.9 (d, *J* = 3.8 Hz), 47.7, 45.7, 36.4, 35.6, 30.9, 30.5, 19.8 (d, *J* = 20.4 Hz), 19.4 (d, *J* = 15.3 Hz), 18.7 (d, *J* = 12.3 Hz), 18.3, 17.6, 17.3; ^31^P NMR (243 MHz, DMSO) δ 47.03, 46.76; MS (ESI) *m*/*z* calcd. for C_30_H_42_N_4_O_6_P^–^ [M—H]^−^ = 585.3, found 585.3; HRMS (ESI) *m*/*z* calcd. for C_30_H_43_N_4_O_6_P [M + H]^+^ 587.2992, found 587.2993; HPLC: DAD1A/B, Sig = 220/254:16.106, 24.295, 32.821, purity = 94%

#### Bis­(3-(((S)-1-amino-4-methyl-1-oxopentan-2-yl)­amino)-2-benzyl-3-oxopropyl)­phosphinic Acid (9d)


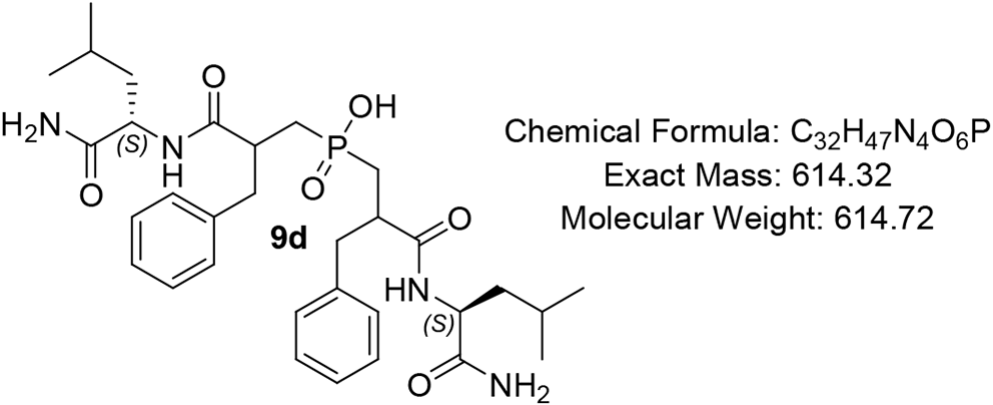


White solid (80%); R*f* (DCM/MeOH/AcOH = 7/0.5/0.5) 0.22. ^1^H NMR (600 MHz, DMSO-*d*_6_) δ 8.19 (d, *J* = 8.1 Hz, 1H), 8.06 – 8.01 (m, 1H), 7.92 (d, *J* = 8.0 Hz, 1H), 7.68 (s, 1H), 7.30 – 7.10 (m, 10H), 6.93 – 6.87 (m, 1H), 6.77 (s, 1H), 4.20 – 4.07 (m, 1H), 3.97 – 3.84 (m, 1H), 3.00 – 2.85 (m, 3H), 2.78 – 2.64 (m, 3H), 2.15 – 2.11 (m, 2H), 2.04 – 1.95 (m, 1H), 1.67 – 1.61 (m, 2H), 1.56 – 1.52 (m, 2H), 1.50 – 1.44 (m, 1H), 1.43 – 1.37 (m, 1H), 1.28 – 1.17 (m, 1H), 0.99 (app d, *J* = 7.1 Hz, 2H), 0.88 (app d, *J* = 6.6 Hz, 1H), 0.84 (app t, *J* = 7.4 Hz, 2H), 0.79 (app d, *J* = 6.4 Hz, 1H), 0.68 (dd, *J* = 26.3, 6.6 Hz, 3H), 0.55 (dd, *J* = 32.1, 6.4 Hz, 3H); ^13^C NMR (151 MHz, DMSO) δ 175.3, 175.2, 174.7 (d, *J* = 4.7 Hz), 139.5 (d, *J* = 8.0 Hz), 139.3 (d, *J* = 13.8 Hz), 129.6, 129.4 (d, *J* = 4.4 Hz), 129.3, 128.6 (d, *J* = 8.0 Hz), 128.5 (d, *J* = 8.5 Hz), 51.6 (d, *J* = 4.4 Hz), 51.3 (d, *J* = 19.0 Hz), 45.7, 40.7, 36.4, 35.6, 30.9, 30.5, 24.6 (d, *J* = 8.3 Hz), 23.9, 23.8, 23.7, 23.6, 22.0, 21.9, 21.37 (d, *J* = 10.3 Hz), 18.3, 12.5; ^31^P NMR (243 MHz, DMSO) δ 47.07, 46.95; MS (ESI) *m*/*z* calcd. for C_32_H_46_N_4_O_6_P ^–^ [M—H]^−^ = 613.3, found 613.3; HRMS (ESI) *m*/*z* calcd. for C_32_H_47_N_4_O_6_P [M + H]^+^ 615.3305, found 615.3304; HPLC DAD1A/B, Sig = 220/254:27.4, 38.8, 52.2, purity = 96%

#### Bis­(3-(((S)-1-amino-1-oxo-3-phenylpropan-2-yl)­amino)-2-benzyl-3-oxopropyl)­phosphinic Acid (9e)


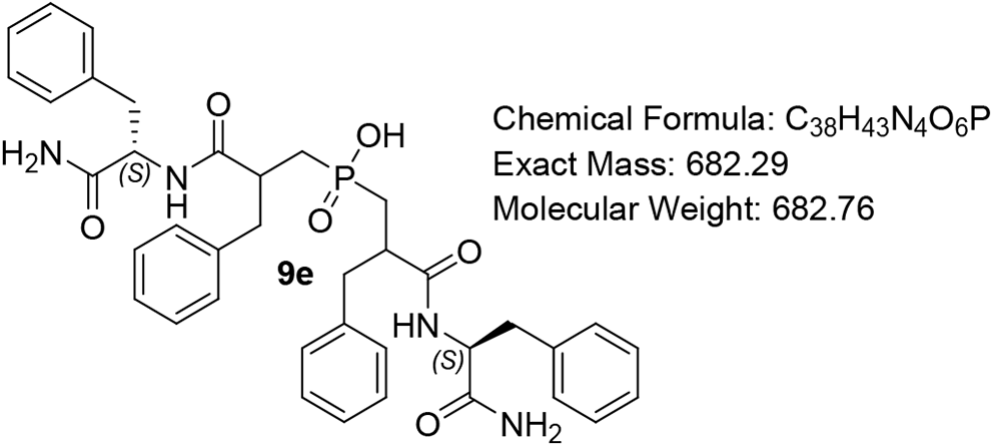


White solid (80%); R*f* (DCM/MeOH/AcOH = 7/0.5/0.5) 0.24. ^1^H NMR (600 MHz, DMSO-*d*_6_) δ 8.28 (ddd, *J* = 54.8, 35.5, 8.5 Hz, 2H), 7.39 (d, *J* = 7.2 Hz, 1H), 7.31 (d, *J* = 9.6 Hz, 1H), 7.24 – 7.10 (m, 15H), 7.08 – 6.94 (m, 5H), 4.39 – 4.27 (m, 2H), 3.12 – 3.04 (m, 2H), 2.93 – 2.75 (m, 3), 2.74 – 2.62 (m, 2H), 2.36 – 2.27 (m, 1H), 2.17 – 2.00 (m, 2H), 1.93 – 1.71 (m, 2H), 1.59 (*app* d, *J* = 19.2 Hz, 2H); ^13^C NMR (151 MHz, DMSO) δ 174.1 (d, *J* = 17.1 Hz), 173.6, 139.7 (d, *J* = 5.5 Hz), 139.3, 139.2, 139.1, 139.0, 138.9 (d, *J* = 3.5 Hz), 129.7, 129.6, 129.5, 129.4, 128.6, 128.5, 128.4, 126.6, 126.5, 126.5, 47.7, 45.7, 40.7, 36.4, 35.6, 34.5, 31.7, 30.9, 30.5, 18.3, 12.6; ^31^P NMR (243 MHz, DMSO) δ 47.31, 47.17, 46.75; MS (ESI) *m*/*z* calcd. for C_38_H_42_N_4_O_6_P ^–^ [M—H]^−^ = 681.8, found 681.8. HRMS (ESI) *m*/*z* calcd. for C_38_H_43_N_4_O_6_PNa [M + Na]^+^ 705.2812, found 705.2809; HPLC DAD1A/B, Sig = 220/254:34.3, 40.0, 47.1, purity = 96%

#### Bis­(2-benzyl-3-((S)-2-carbamoylpyrrolidin-1-yl)-3-oxopropyl)­phosphinic Acid (9f)


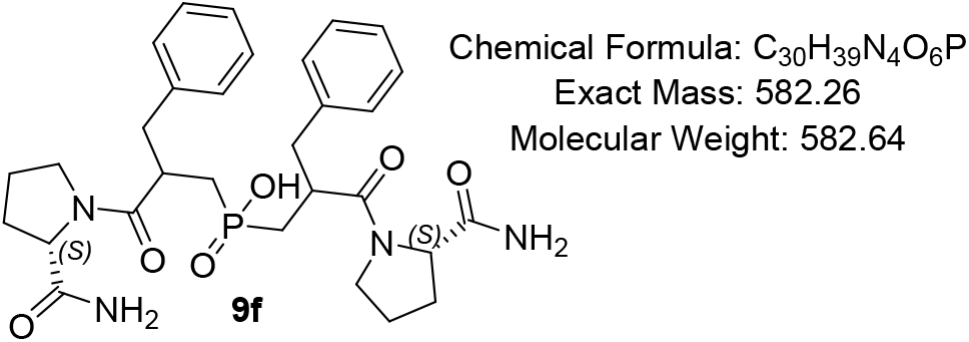


Pale-yellow oil (78%); R*f* (DCM/MeOH/AcOH = 7/1/0.5) 0.22. ^1^H NMR (600 MHz, DMSO-*d*_6_) δ 7.85 – 7.78 (m, 1H), 7.52 (s, 1H), 7.32 – 7.11 (m, 10H), 6.95 – 6.92 (m, 1H), 6.86 – 6.80 (m, 1H), 4.08 – 3.98 (m, 1H), 3.52 – 3.42 (m, 1H), 3.41 – 3.30 (m, 1H), 3.08 – 2.93 (m, 2H), 2.79 – 2.67 (m, 2H), 2.64 – 2.60 (m, 1H), 2.21 – 2.12 (m, 2H), 2.07 – 1.99 (m, 1H), 1.93 – 1.86 (m, 1H), 1.82 – 1.75 (m, 3H), 1.73 – 1.68 (m, 1H), 1.64 (app t, *J* = 3.0 Hz, 1H), 1.62 – 1.41 (m, 6H), 0.99 (app d, *J* = 7.0 Hz, 2H); ^13^C NMR (151 MHz, DMSO) δ 174.3 (d, *J* = 3.1 Hz), 174.2, 172.9, 172.7 (d, *J* = 2.6 Hz), 158.9, 158.7, 139.2, 129.8, 129.7, 129.4, 129.3, 128.8, 128.7, 128.6, 126.9, 126.8, 117.6, 115.6, 87.9, 60.2 (d, *J* = 10.6 Hz), 47.4, 46.8, 46.7, 45.7, 36.4, 35.6, 30.9, 30.5, 29.6 (d, *J* = 8.4 Hz), 29.5, 24.6, 24.0, 23.9, 18.3, 12.5; ^31^P NMR (243 MHz, DMSO) δ 47.32, 46.55, 46.35, 46.24, 46.22; MS (ESI) *m*/*z* calcd. for C_30_H_38_N_4_O_6_P ^–^ [M—H]^−^ = 581.3, found 581.3; HRMS (ESI) *m*/*z* calcd. for C_30_H_39_N_4_O_6_P [M + H]^+^ 583.2679, found 583.2678; HPLC: DAD1A/B, Sig = 220/254:16.740, 20.416, 25.200, purity = 92%

#### Bis­(3-(((S)-1-amino-3-(1H-indol-3-yl)-1-oxopropan-2-yl)­amino)-2-benzyl-3 oxopropyl)­phosphinic Acid (9g)


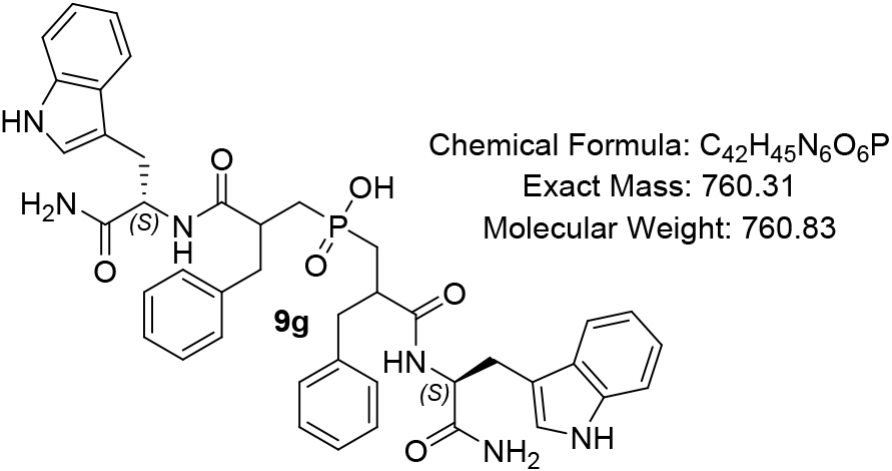


White Solid (75%); R*f* (DCM/MeOH/AcOH = 7/1/0.5) 0.13. ^1^H NMR (600 MHz, DMSO-*d*_6_) δ 8.37 – 7.95 (m, 2H), 7.78 – 7.45 (m, 3H), 7.40 – 7.29 (m, 2H), 7.28 – 7.07 (m, 10H), 7.07 – 7.01 (m, 3H), 7.00 – 6.91 (m, 5H), 6.91 – 6.68 (m, 4H), 4.51 – 4.32 (m, 1H), 3.58 – 3.38 (m, 1H), 3.02 – 2.61 (m, 5H), 2.14 – 2.03 (m, 6H), 1.92 – 1.85 (m, 3H); ^13^C NMR (151 MHz, DMSO) δ 174.6, 174.5, 174.1, 173.8, 173.7, 173.5, 159.2, 159.0, 158.7, 158.5, 136.6, 129.7, 129.5, 129.0, 128.5, 127.7, 126.6, 126.5, 124.0, 121.2, 119.1, 118.9, 118.6, 117.2, 115.2, 113.3, 111.7, 111.1, 110.9, 55.5, 49.1, 43.9, 42.1, 36.7, 29.0, 28.9, 28.6, 28.6, 18.3, 12.6; ^31^P NMR (243 MHz, DMSO) δ 47.50, 47.47, 47.46, 47.38, 47.33, 47.22, 47.04, 46.96, 46.85, 46.81; MS (ESI) *m*/*z* calcd. *m*/*z* calcd for C_42_H_44_N_4_O_6_P ^–^ [M—H]^−^ 760.3, found 760.3; HRMS (ESI) *m*/*z* calcd. for C_42_H_45_N_4_O_6_P [M + H]^+^ 761.3210, found 761.3207; HPLC: DAD1A/B, Sig = 220/254:20.2, 24.9, 30.8, purity = 60%

## Supplementary Material



## Notes

^1^H NMR, ^13^C NMR, ^3^P NMR, and HRMS spectra, along with the HPLC chromatograms of the most potent compound 9c and its three resolved diastereoisomers (PDF)
