# Supplementary material for: Highly Potent Phosphinic HIV‑1 Protease Inhibitors: Synthesis, In Vitro Evaluation, and Docking Studies
Source: ACS Omega. 2025 Dec 10;10(50):62083–98. doi: 10.1021/acsomega.5c09361 (PMC12750381; doi:10.1021/acsomega.5c09361)
Supplement: Supplementary file 1 [file ao5c09361_si_001.pdf]

SUPPORTING INFORMATION

Highly potent phosphinic HIV-1 protease inhibitors: Synthesis, *in-vitro* evaluation, and docking studies

Komal Hayat[1], Danwen Qiu[1], Yuanyuan Wang[2], Palmer Sivoko Imbenzi[1], Faez Iqbal Khan[2], Magdalini Matziari[1,*]

[1]*Department of Chemistry and Materials Science, School of Science, Xi'an Jiaotong-Liverpool University, 111 Ren'ai Road, SIP, Suzhou, Jiangsu Province, 215123, P. R. China*

[2]*Department of Biosciences and Bioinformatics, School of Science, Xi'an Jiaotong-Liverpool University, 111 Ren'ai road, SIP, Suzhou, Jiangsu Province, 215123, P. R. China*

Copies of ^{1}H NMR, ^{13}C NMR, ^{31}P NMR, and HRMS Spectra plus HPLC Chromatograms

*Figure S1. ^{1}HNMR of Compound **3e*** 4
*Figure S2. ^{13}CNMR of Compound **3e*** 4
*Figure S3. ^{31}PNMR of Compound **3e*** 5
*Figure S4. ^{1}HNMR of Compound **4e*** 5
*Figure S5. ^{13}CNMR of Compound **4e*** 6
*Figure S6. ^{31}PNMR of Compound **4e*** 6
*Figure S7. HRMS spectrum of Compound **4e*** 7
*Figure S8. ^{1}HNMR of Compound **5*** 7
*Figure S9.^{13}CNMR of Compound **5*** 8
*Figure S10. ^{31}PNMR of Compound **5*** 8
*Figure S11. ^{1}HNMR of Compound **6*** 9
*Figure S12. ^{13}CNMR of Compound **6*** 9
*Figure S13. ^{31}PNMR of Compound **6*** 10
*Figure S14. ^{1}HNMR of Compound **8a*** 10
*Figure S15. ^{13}CNMR of Compound **8a*** 11
*Figure S16. ^{31}PNMR of Compound **8a*** 11
*Figure S17. ^{1}HNMR of Compound **8b*** 12
*Figure S18. ^{13}CNMR of Compound **8b*** 12
*Figure S19. ^{31}PNMR of Compound **8b*** 13
*Figure S20. ^{1}HNMR of Compound **8c*** 13
*Figure S21. ^{13}CNMR of Compound **8c*** 14
*Figure S22. ^{31}PNMR of Compound **8c*** 14
*Figure S23. ^{1}HNMR of Compound **8d*** 15
*Figure S24. ^{13}CNMR of Compound **8d*** 15
*Figure S25. ^{31}PNMR of Compound **8d*** 16

*Figure S26. ^{1}HNMR of Compound **8e***...16
*Figure S27. ^{13}CNMR of Compound **8e***...17
*Figure S28. ^{31}PNMR of Compound **8e***...17
*Figure S29. ^{1}HNMR of Compound **8f***...18
*Figure S30. ^{13}CNMR of Compound **8f***...18
*Figure S31. ^{31}PNMR of Compound **8f***...19
*Figure S32. ^{1}HNMR of Compound **8g***...19
*Figure S33. ^{13}CNMR of Compound **8g***...20
*Figure S34. ^{31}PNMR of Compound **8g***...20
*Figure S35. ^{1}HNMR of Compound **9a***...21
*Figure S36. ^{13}CNMR of Compound **9a***...21
*Figure S37. ^{31}PNMR of Compound **9a***...22
*Figure S38. HRMS Spectrum of Compound **9a***...22
*Figure S39. ^{1}HNMR of Compound **9b***...23
*Figure S40. ^{13}CNMR of Compound **9b***...23
*Figure S41. ^{31}PNMR of Compound **9b***...24
*Figure S42. HRMS Spectrum of Compound **9b***...24
*Figure S43. ^{1}HNMR of Compound **9c***...25
*Figure S44. ^{13}CNMR of Compound **9c***...25
*Figure S45. ^{31}PNMR of Compound of **9c***...26
*Figure S46. HRMS Spectrum of Compound **9c***...26
*Figure S47. HPLC chromatogram of Compound **9c***...27
*Figure S48.HPLC chromatogram of Isomer **A** (**9c**)*...27
*Figure S49. HPLC chromatogram of Isomer **B** (**9c**)*...28
*Figure S50. HPLC chromatogram of Isomer **C** (**9c**)*...28
*Figure S51. ^{1}HNMR of Compound **9d***...29
*Figure S52. ^{13}CNMR of Compound **9d***...29
*Figure S53. ^{31}PMNR of Compound **9d***...30
*Figure S54. HRMS Spectrum of Compound **9d***...30
*Figure S55. ^{1}HNMR of Compound **9e***...31
*Figure S56. ^{13}CNMR of Compound **9e***...31
*Figure S57. ^{31}PNMR of Compound **9e***...32
*Figure S58. HRMS Spectrum of Compound **9e***...32
*Figure S59. ^{1}HNMR of Compound **9f***...33
*Figure S60. ^{13}CNMR of Compound **9f***...33
*Figure S61. ^{31}PNMR of Compound **9f***...34
*Figure S62. HRMS Spectrum of Compound **9f***...34
*Figure S63. ^{1}HNMR of Compound **9g***...35
*Figure S64. ^{13}CNMR of Compound **9g***...35
*Figure S65. ^{31}PNMR of Compound **9g***...36
*Figure S66. HRMS Spectrum of Compound **9g***...36
*Figure S67. ^{1}HNMR of Isomer **A** (**9c**)*...37
*Figure S68. ^{13}CNMR of Isomer **A** (**9c**)*...38
*Figure S69. COSY NMR of Isomer **A** (**9c**)*...39
*Figure S70. ^{31}PNMR of Isomer **A** (**9c**)*...39
*Figure S71. ^{1}HNMR of Isomer **B** (**9c**)*...40

*Figure S72. ^{13}CNMR of Isomer **B** (**9c**)* 40
*Figure S73. COSY NMR of Isomer **B** (**9c**)*..41
*Figure S74. ^{31}PNMR of Isomer **B** (**9c**)* 41
*Figure S75. ^{1}HNMR of Isomer **C** (**9c**)* 42
*Figure S76. ^{13}CNMR of Isomer **C** (**9c**)* 42
*Figure S77. COSY NMR of Isomer **C** (**9c**)*..43
*Figure S78. ^{31}PNMR of Isomer **C** (**9c**)* 43

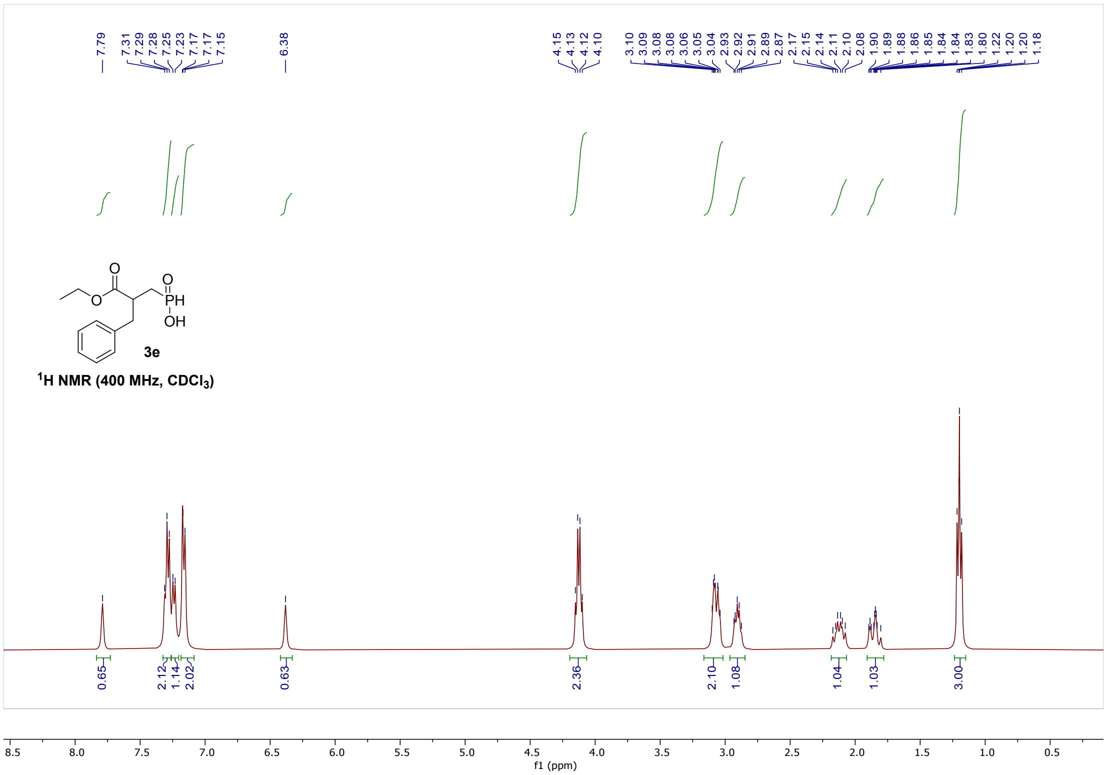

*Figure S1. ¹HNMR of Compound **3e***

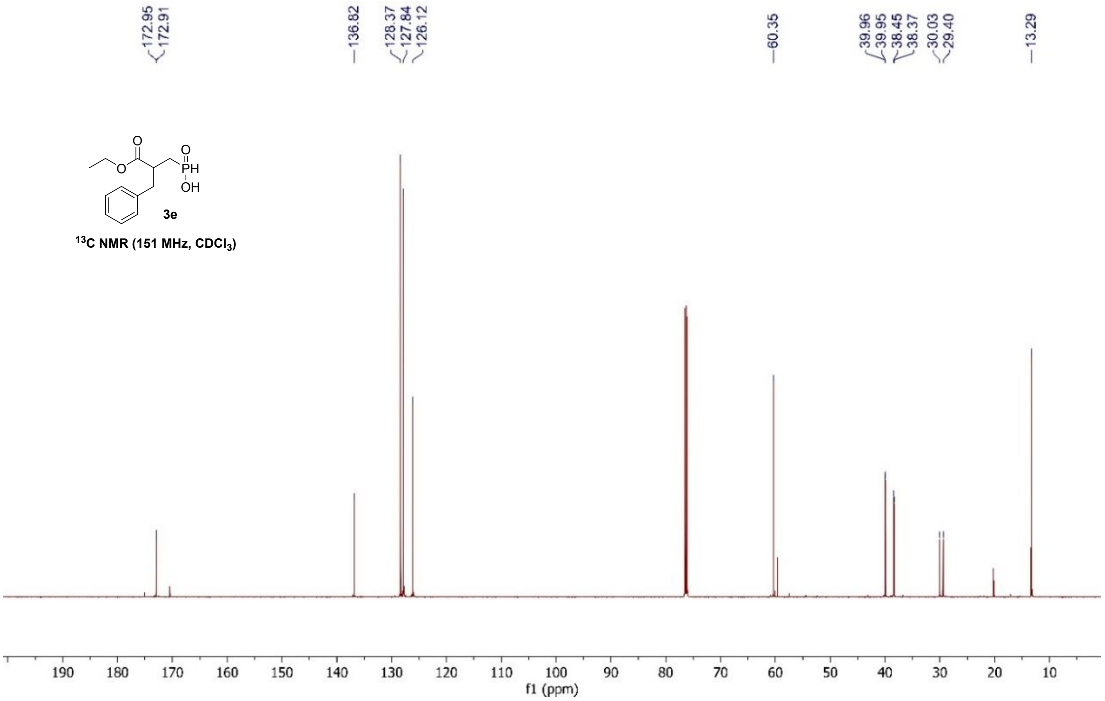

*Figure S2. ¹³CNMR of Compound **3e***

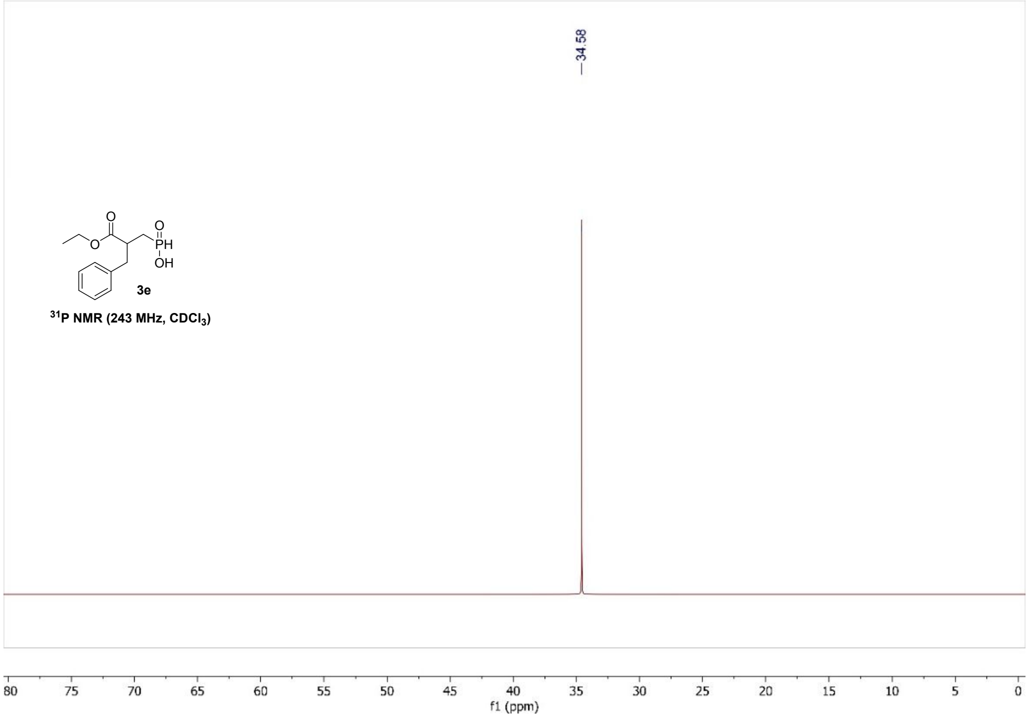

*Figure S3. ^{31}PNMR of Compound **3e***

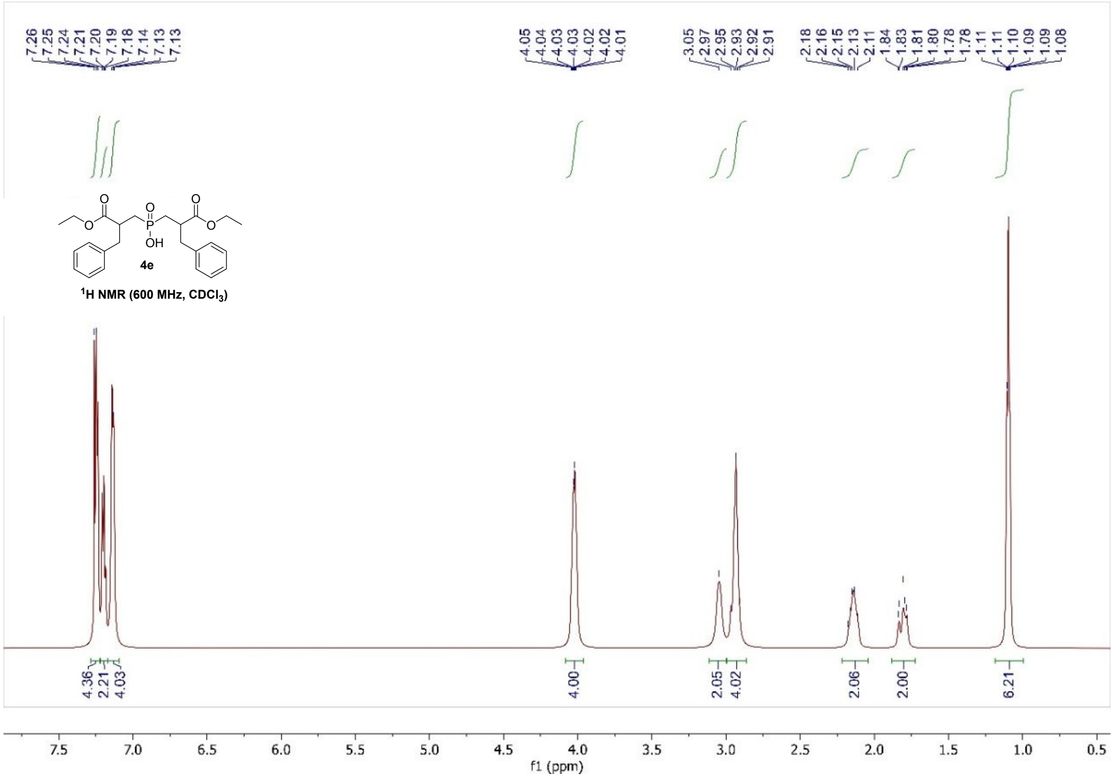

*Figure S4. ^{1}HNMR of Compound **4e***

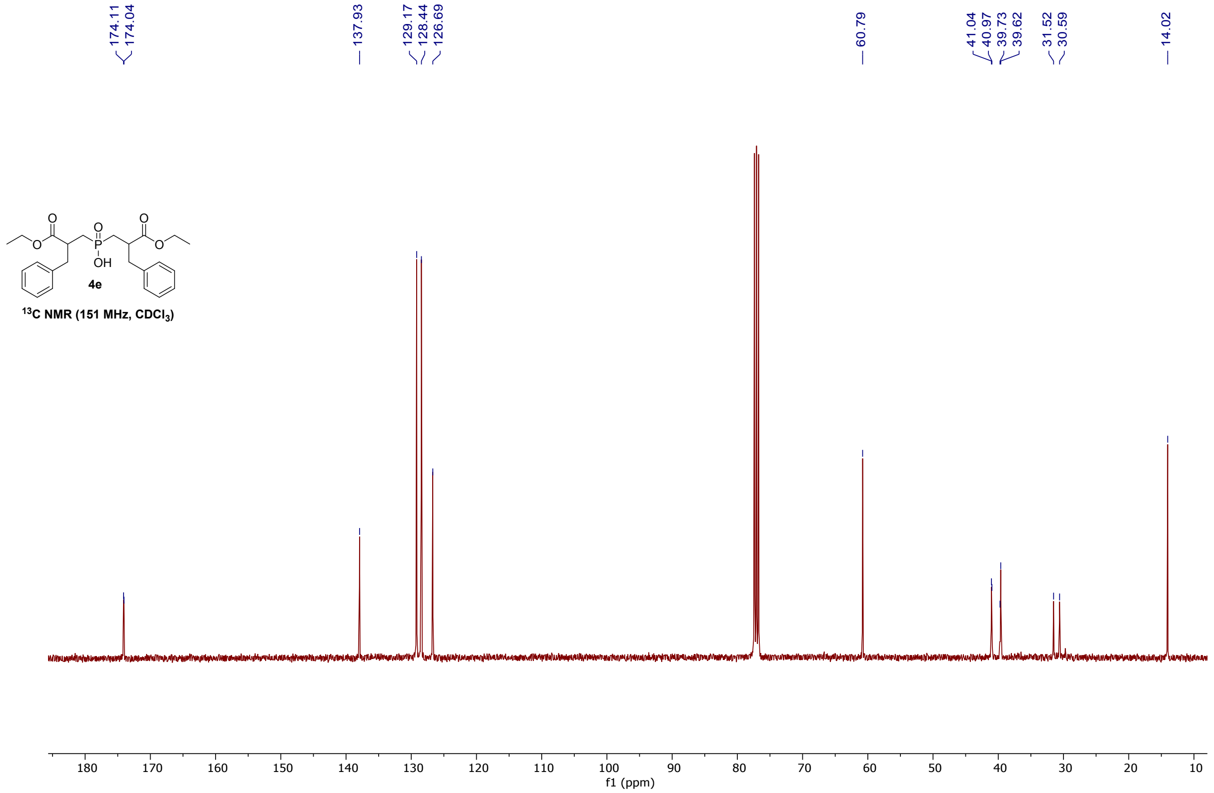

*Figure S5. ^{13}CNMR of Compound **4e***

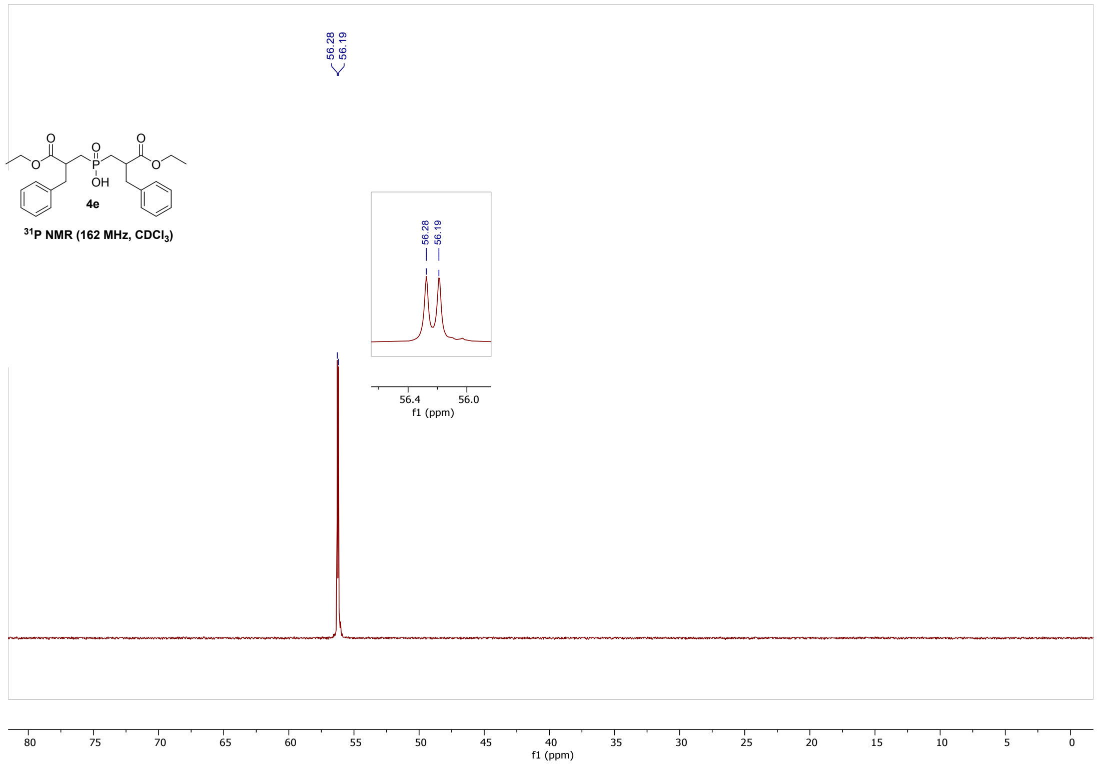

*Figure S6. ^{31}PNMR of Compound **4e***

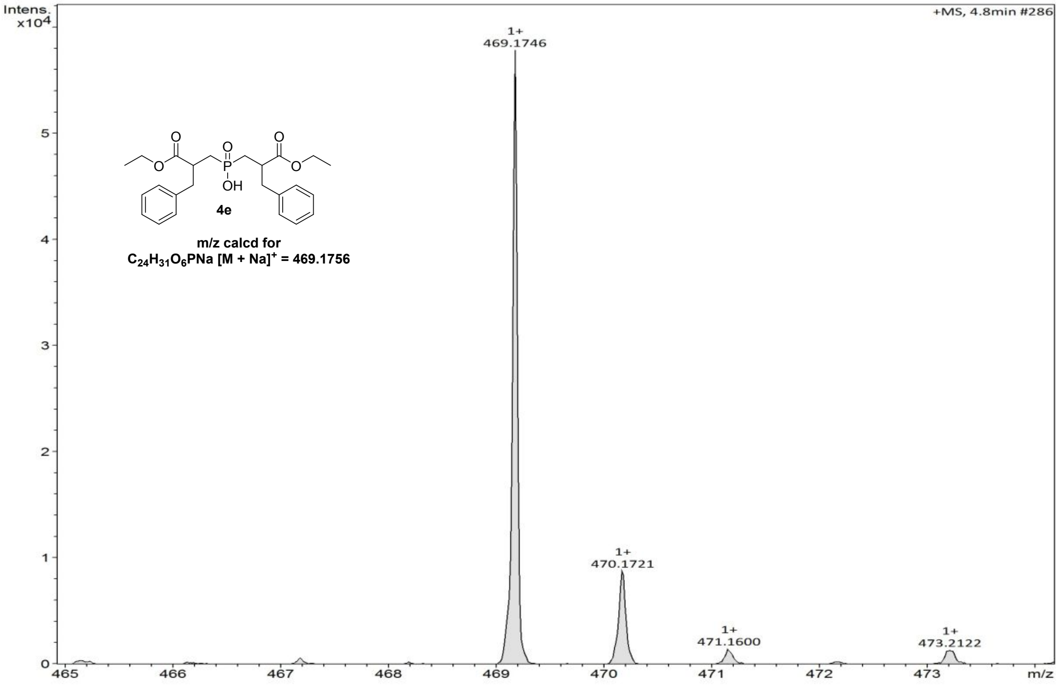

*Figure S7. HRMS spectrum of Compound **4e***

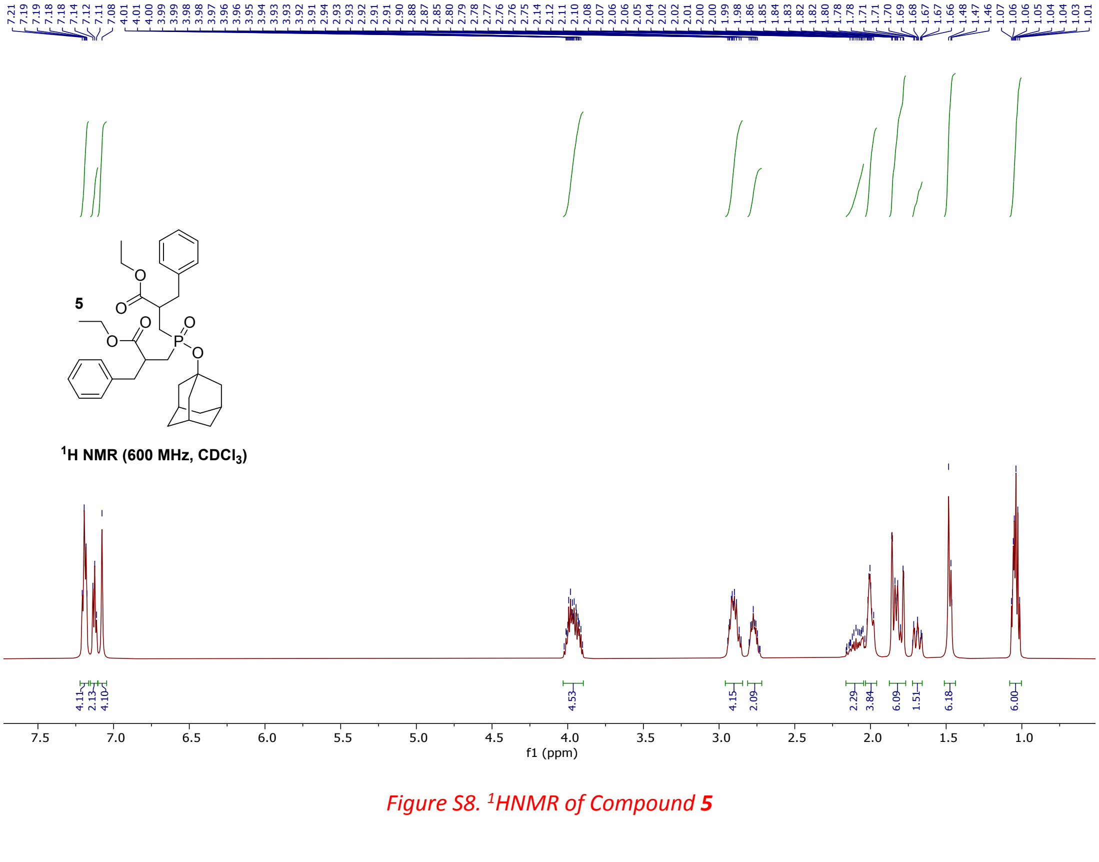

*Figure S8. ^{1}HNMR of Compound **5***

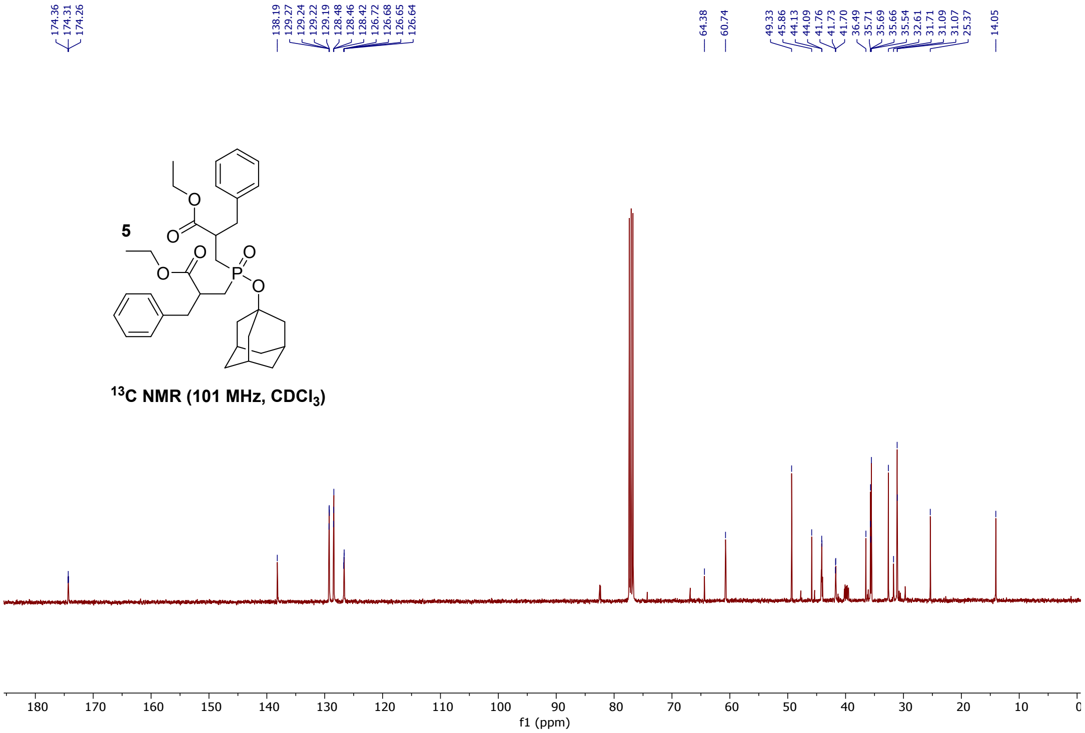

*Figure S9.^{13}CNMR of Compound **5***

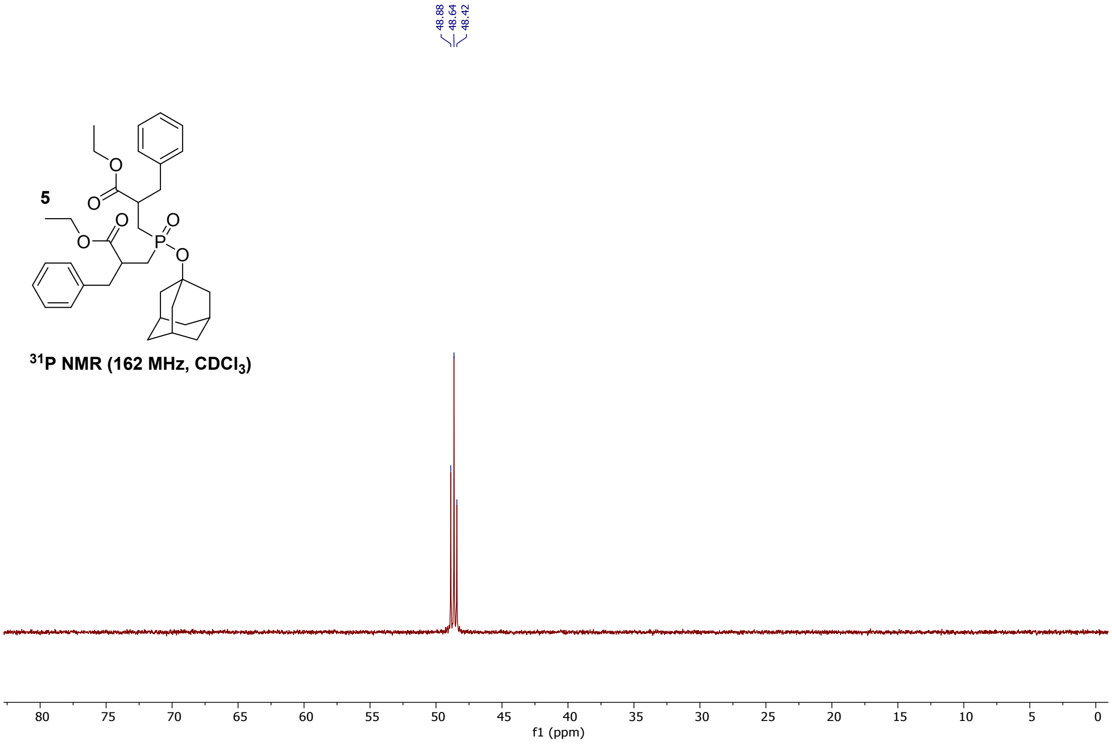

*Figure S10. ^{31}PNMR of Compound **5***

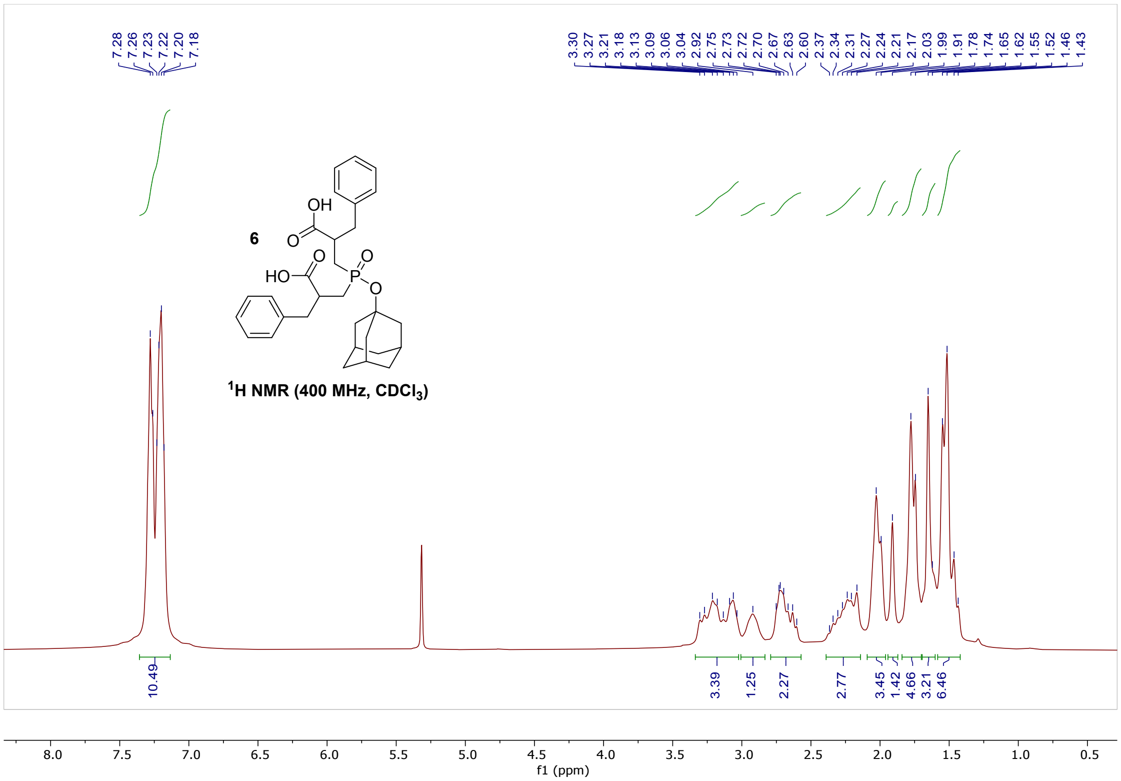

Figure S11. 1HNMR of Compound ***6***

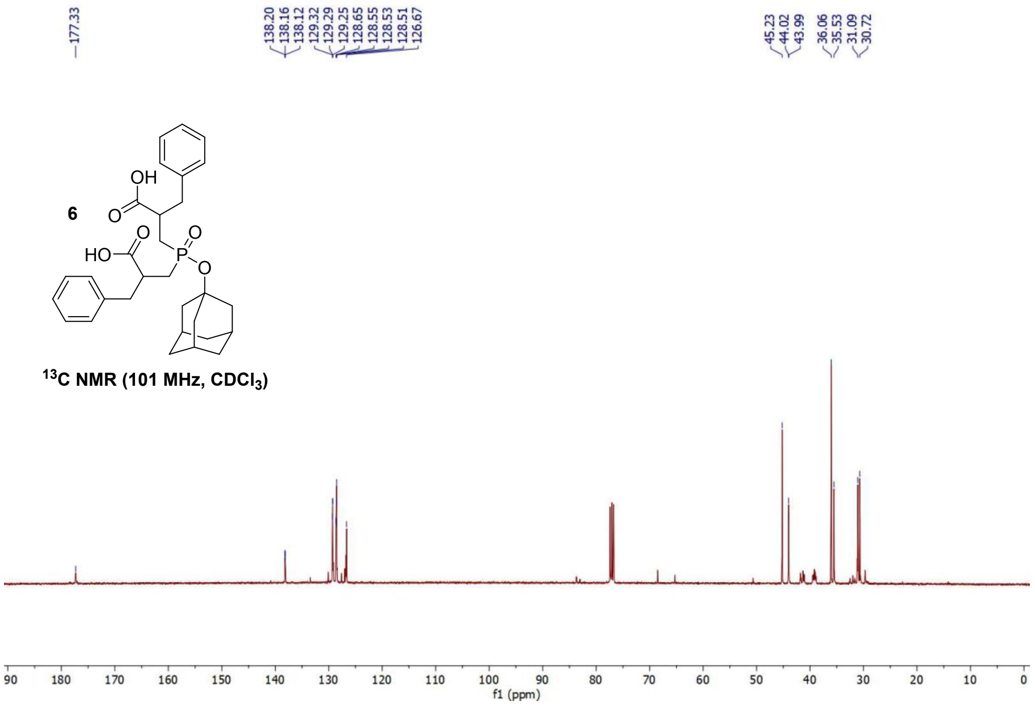

Figure S12. 13CNMR of Compound ***6***

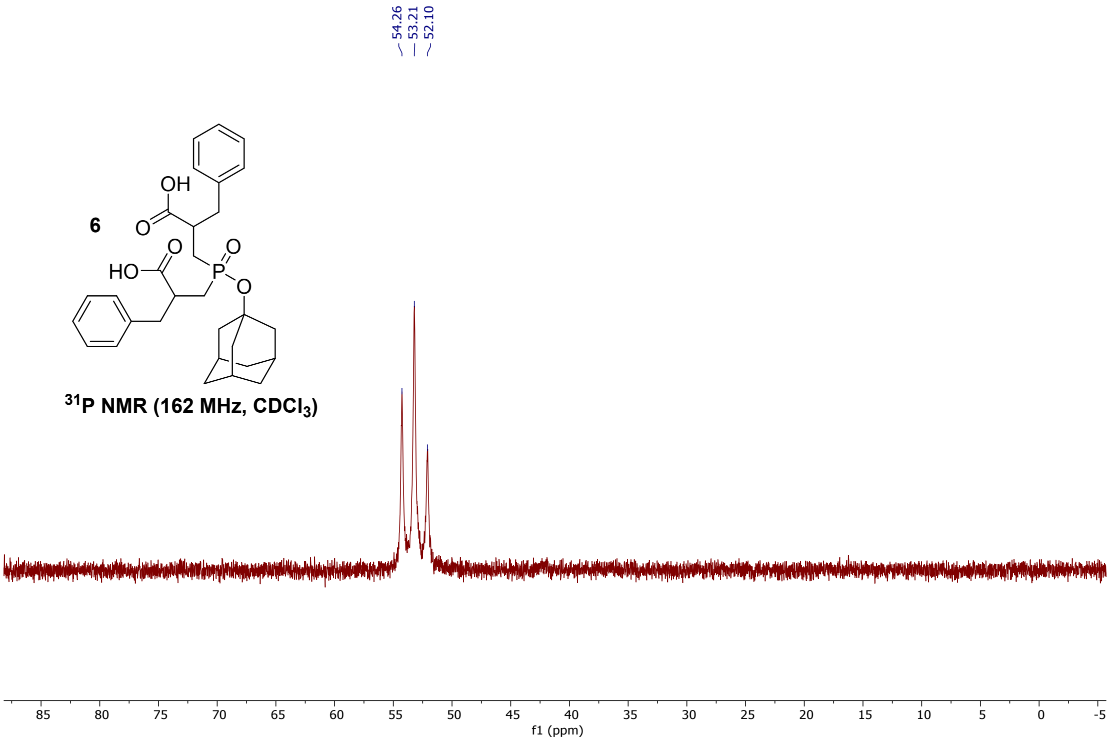

*Figure S13. ^{31}PNMR of Compound **6***

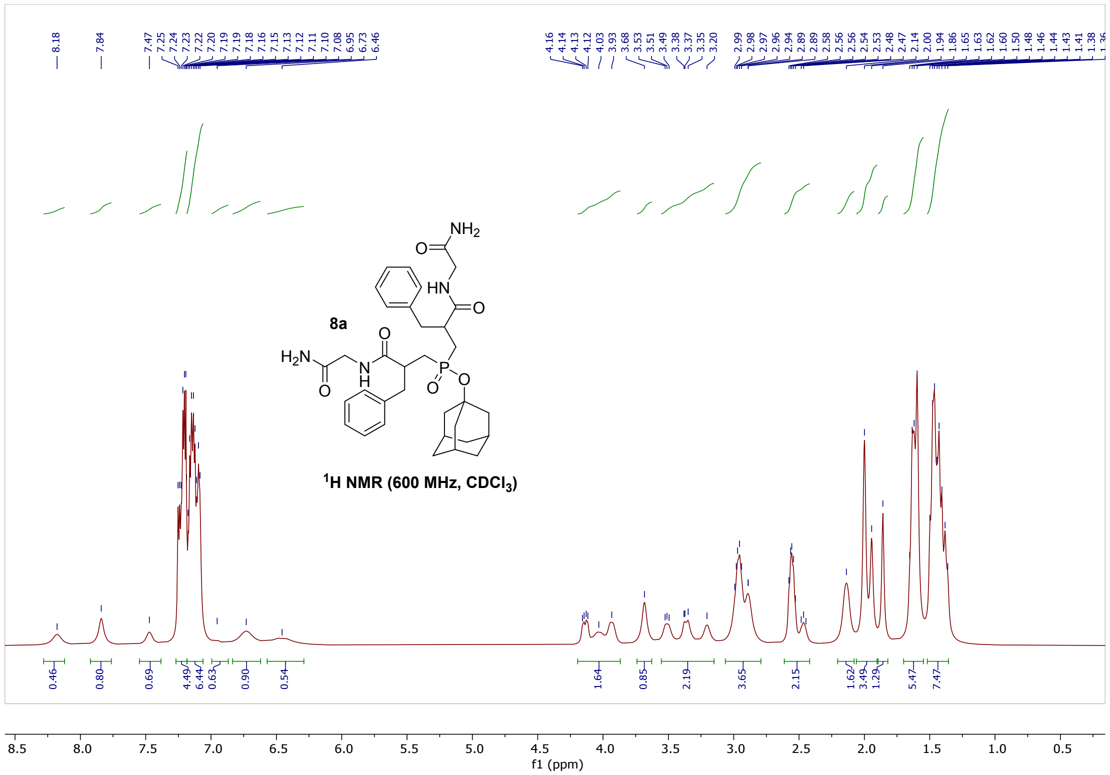

*Figure S14. ^{1}HNMR of Compound **8a***

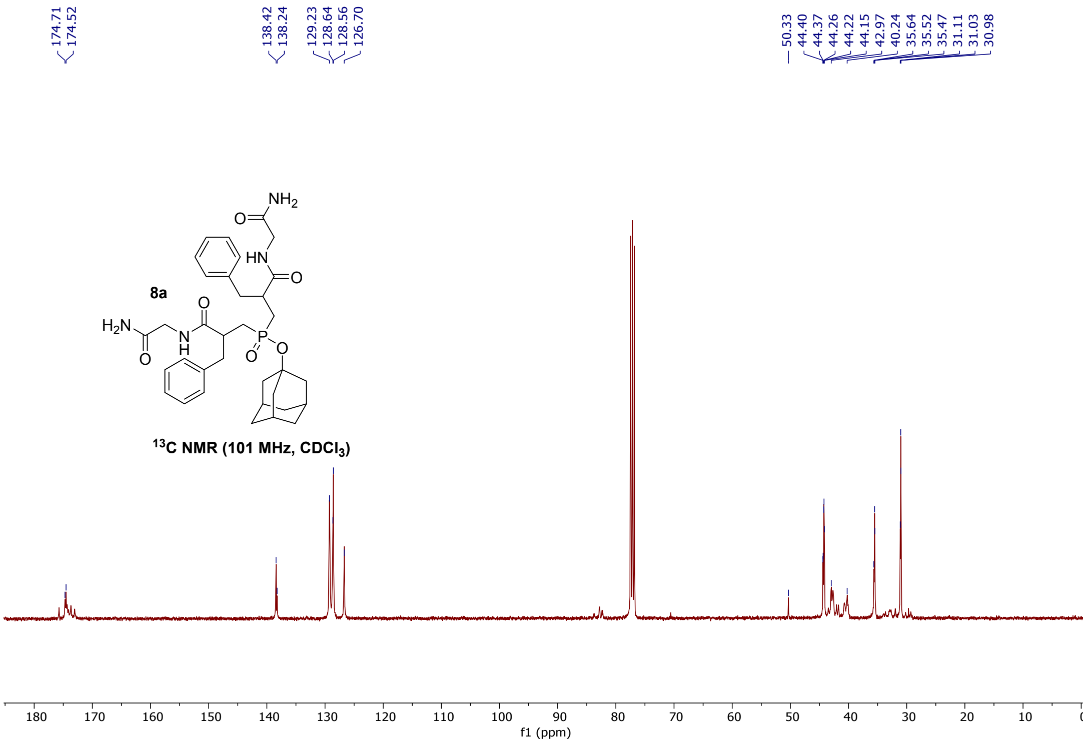

Figure S15. ^{13}CNMR of Compound ***8a***

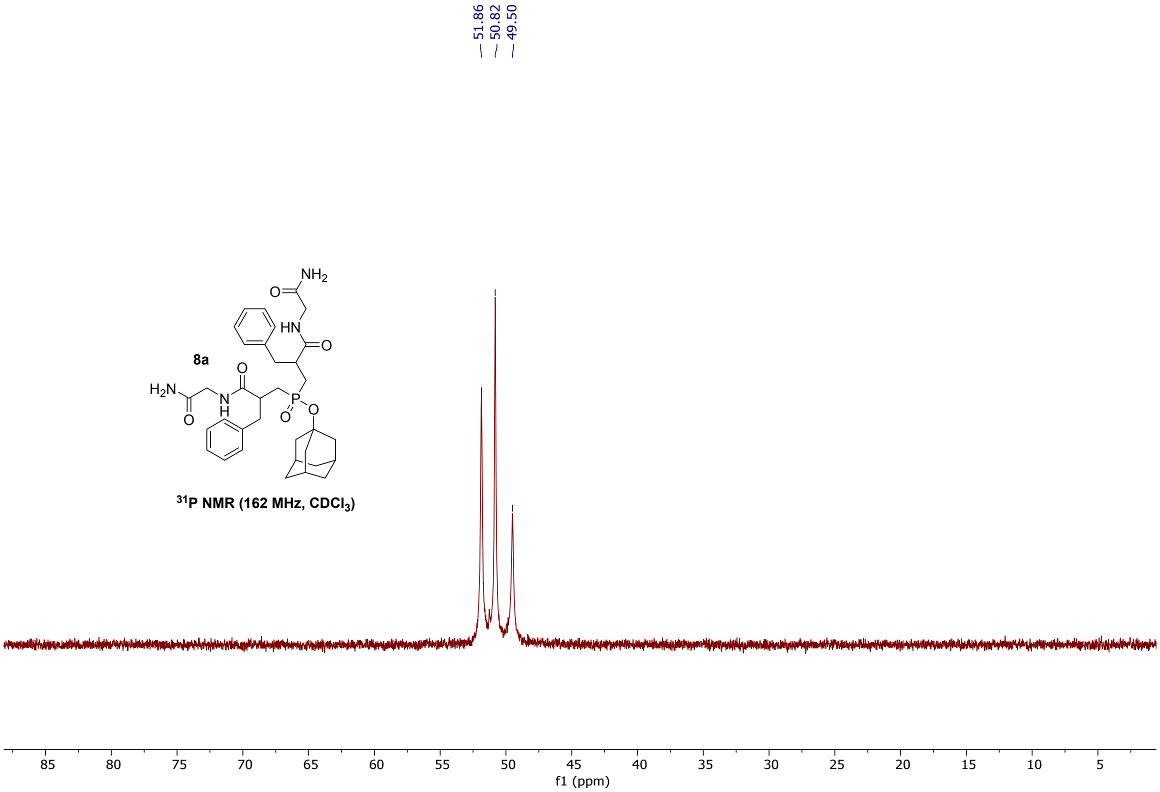

Figure S16. ^{31}PNMR of Compound ***8a***

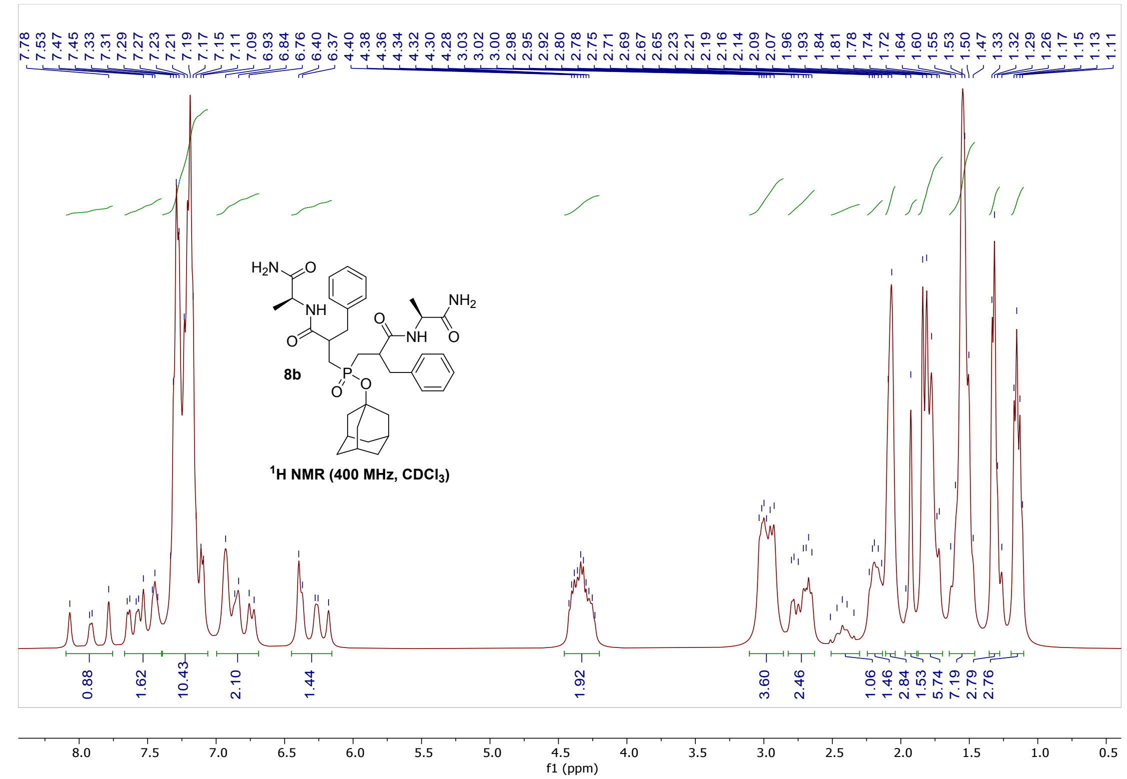

*Figure S17. ^{1}HNMR of Compound **8b***

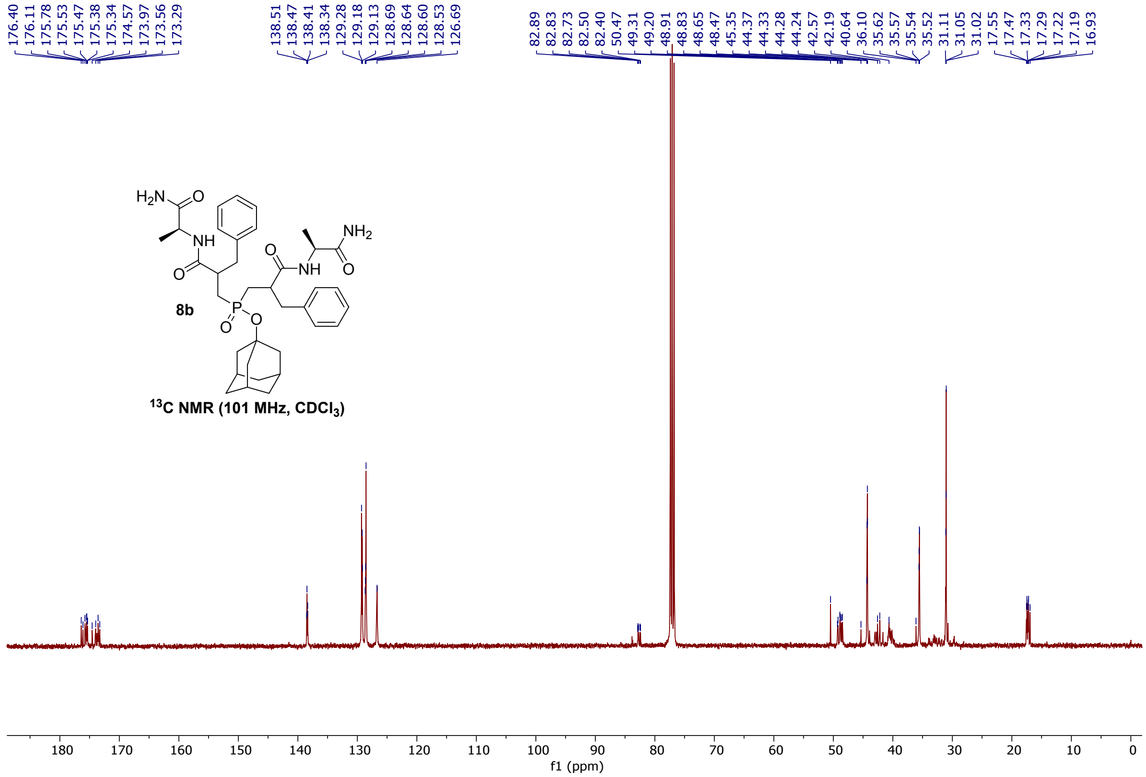

*Figure S18. ^{13}CNMR of Compound **8b***

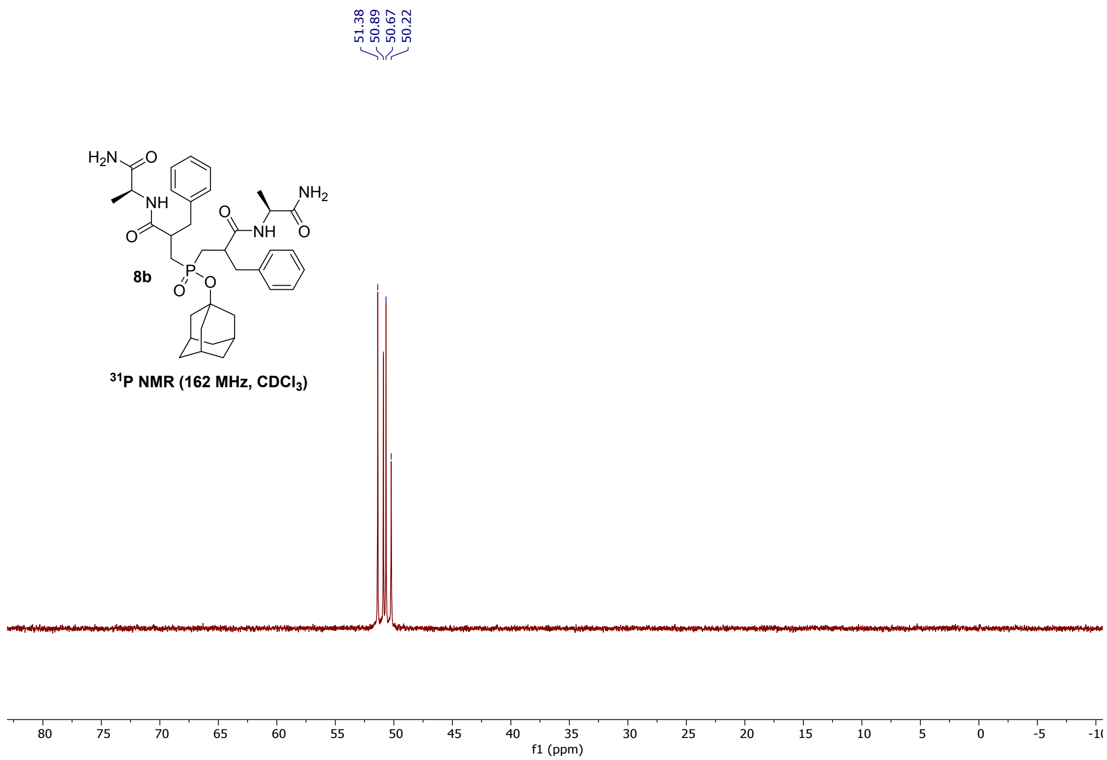

*Figure S19. ^{31}PNMR of Compound **8b***

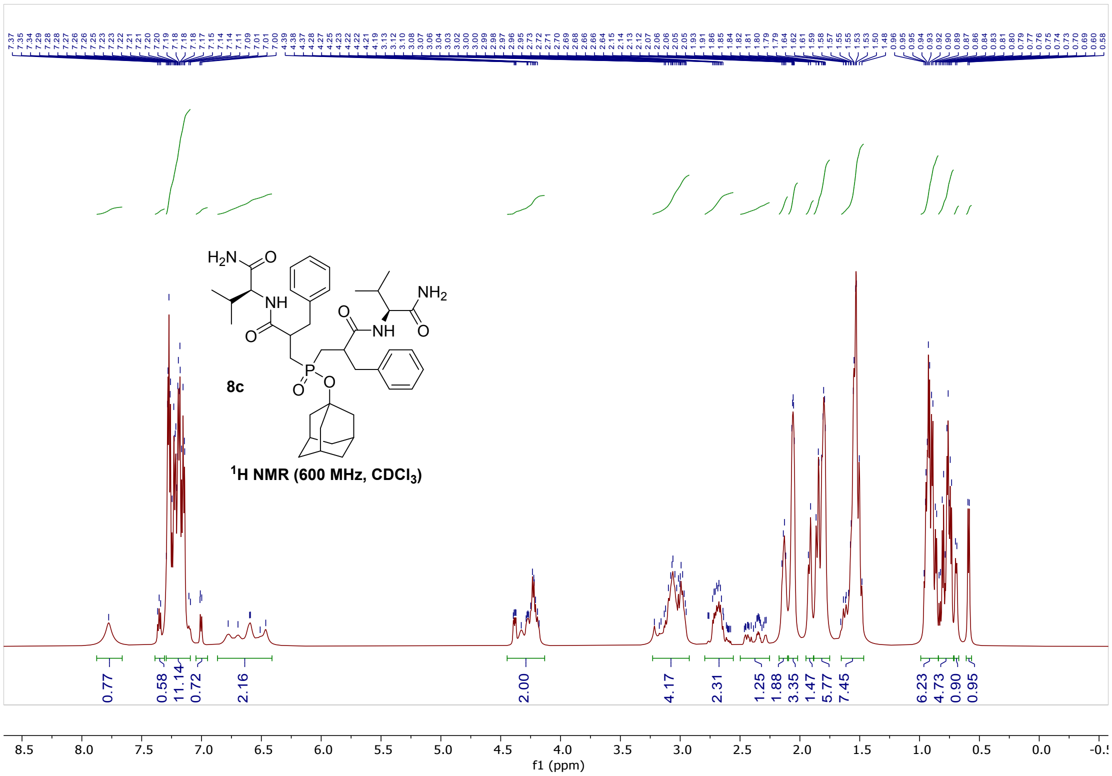

*Figure S20. ^{1}HNMR of Compound **8c***

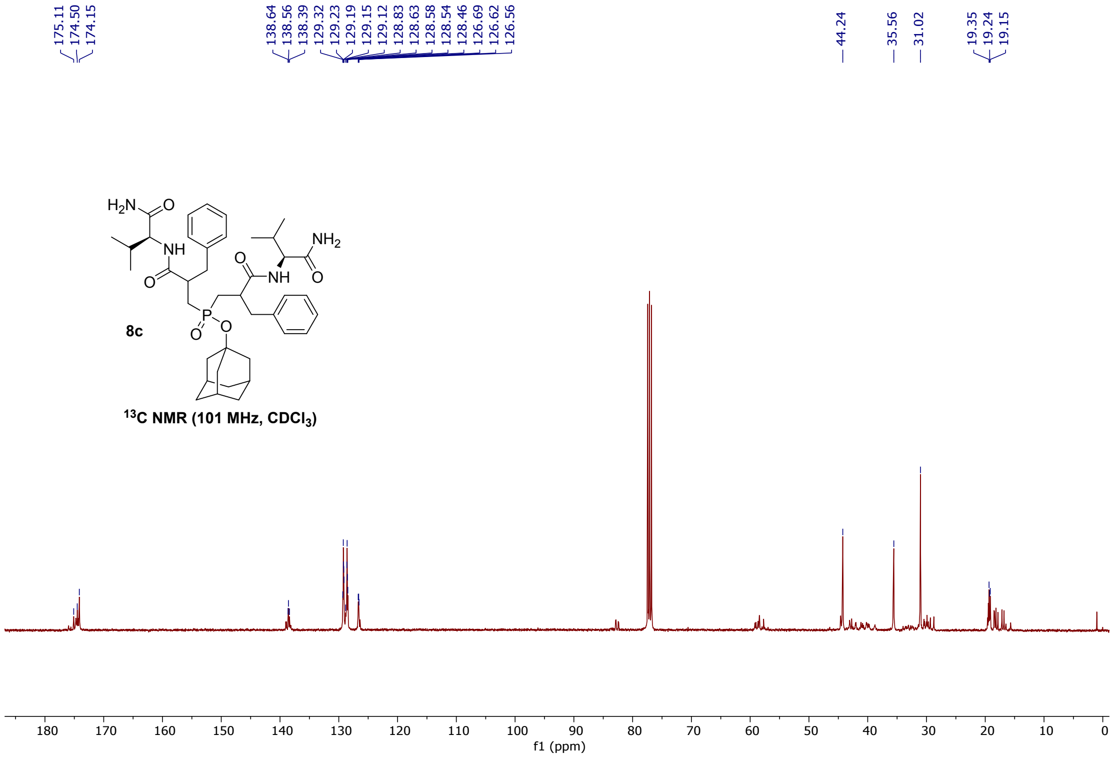

*Figure S21. ^{13}CNMR of Compound **8c***

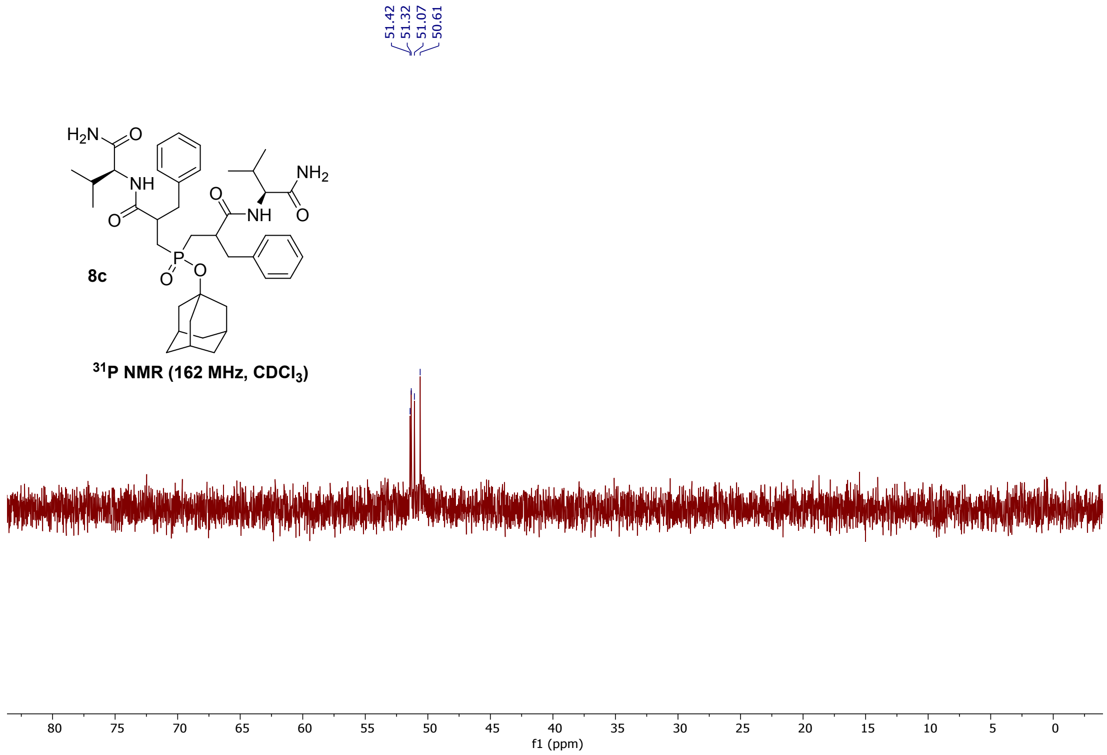

*Figure S22. ^{31}PNMR of Compound **8c***

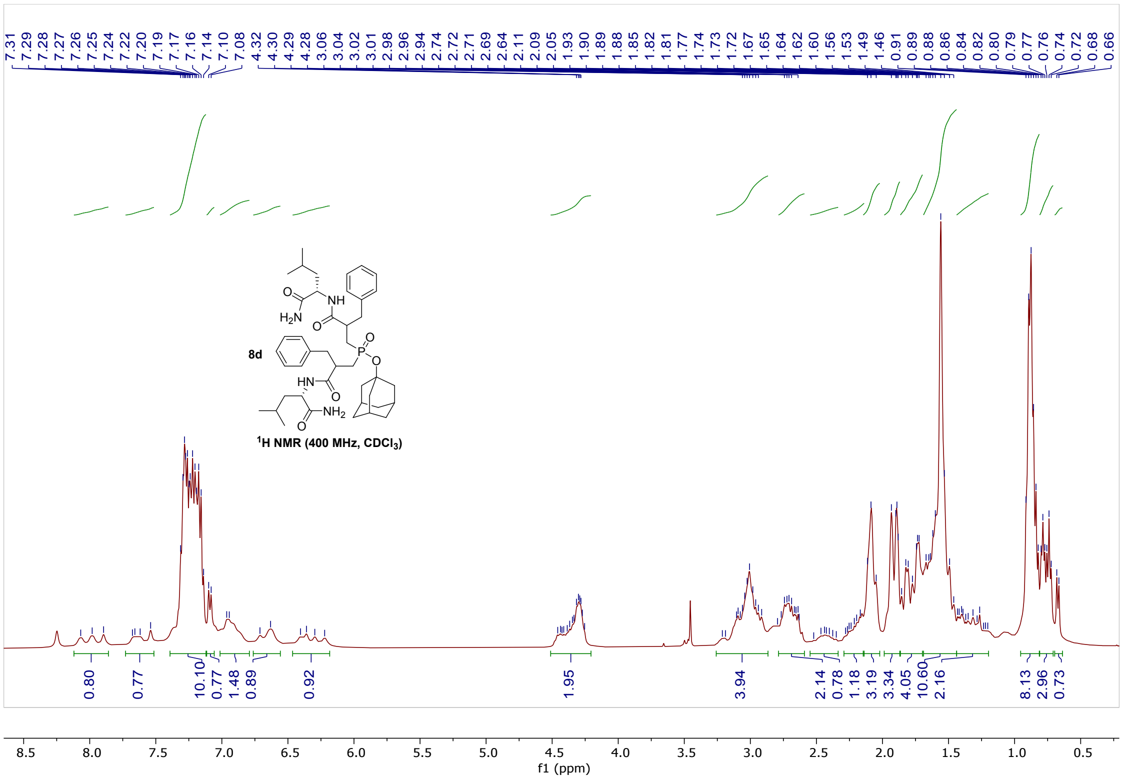

*Figure S23. ^{1}HNMR of Compound **8d***

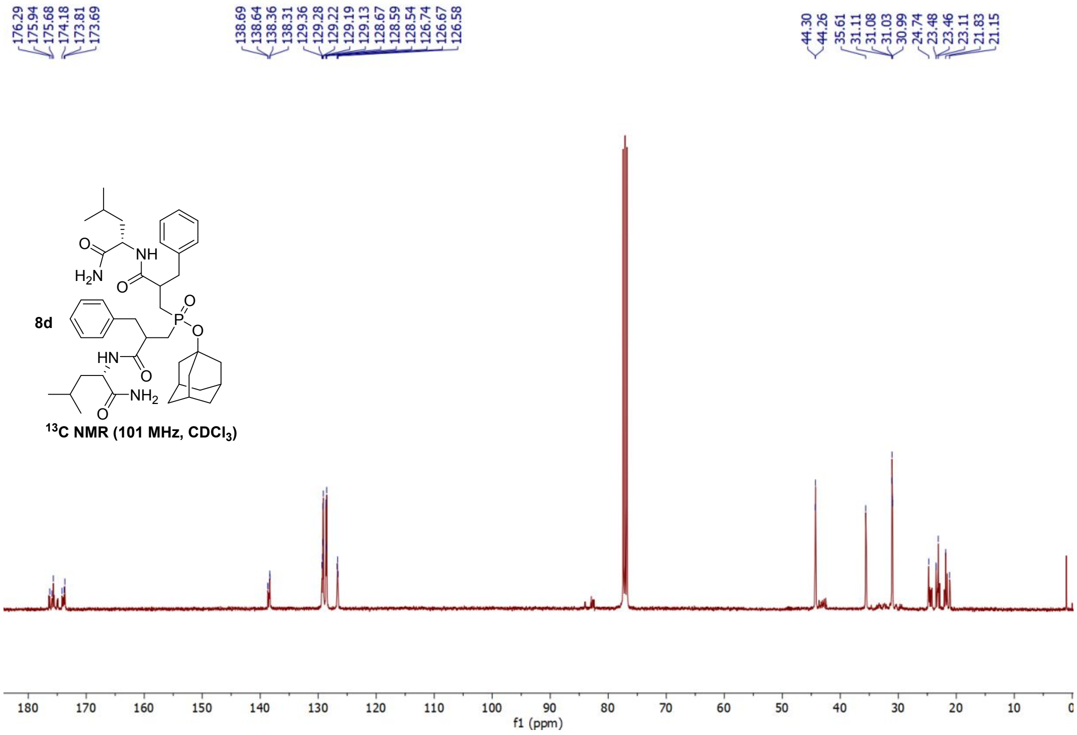

*Figure S24. ^{13}CNMR of Compound **8d***

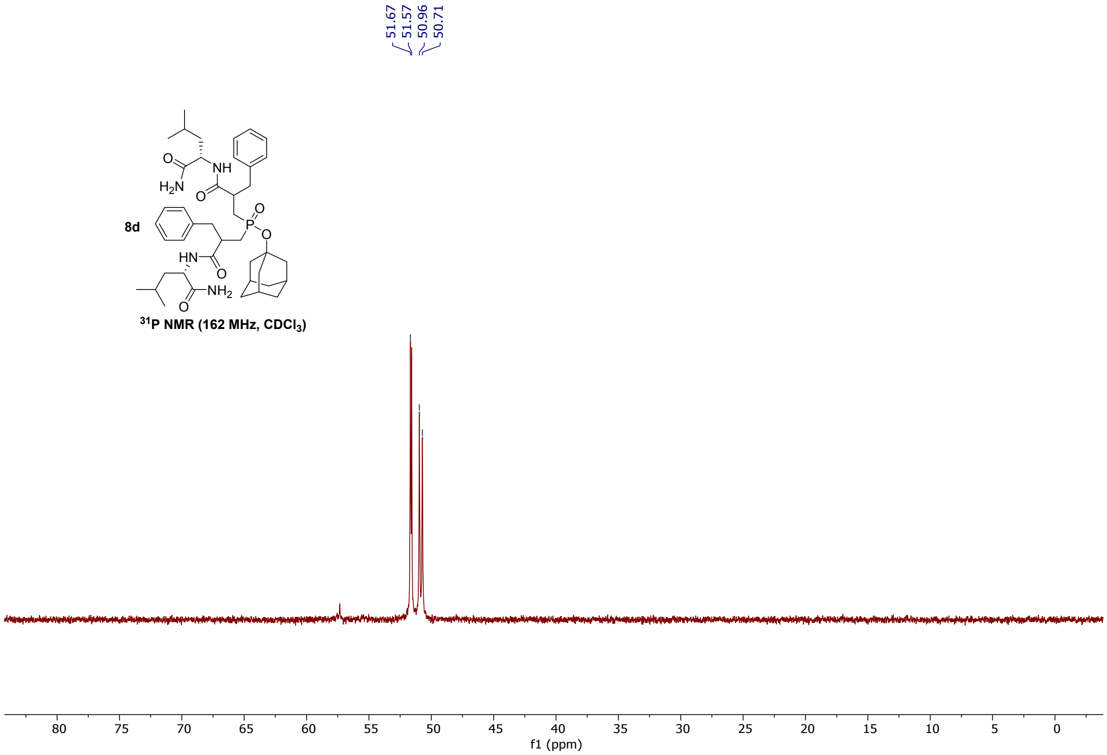

*Figure S25. ^{31}PNMR of Compound **8d***

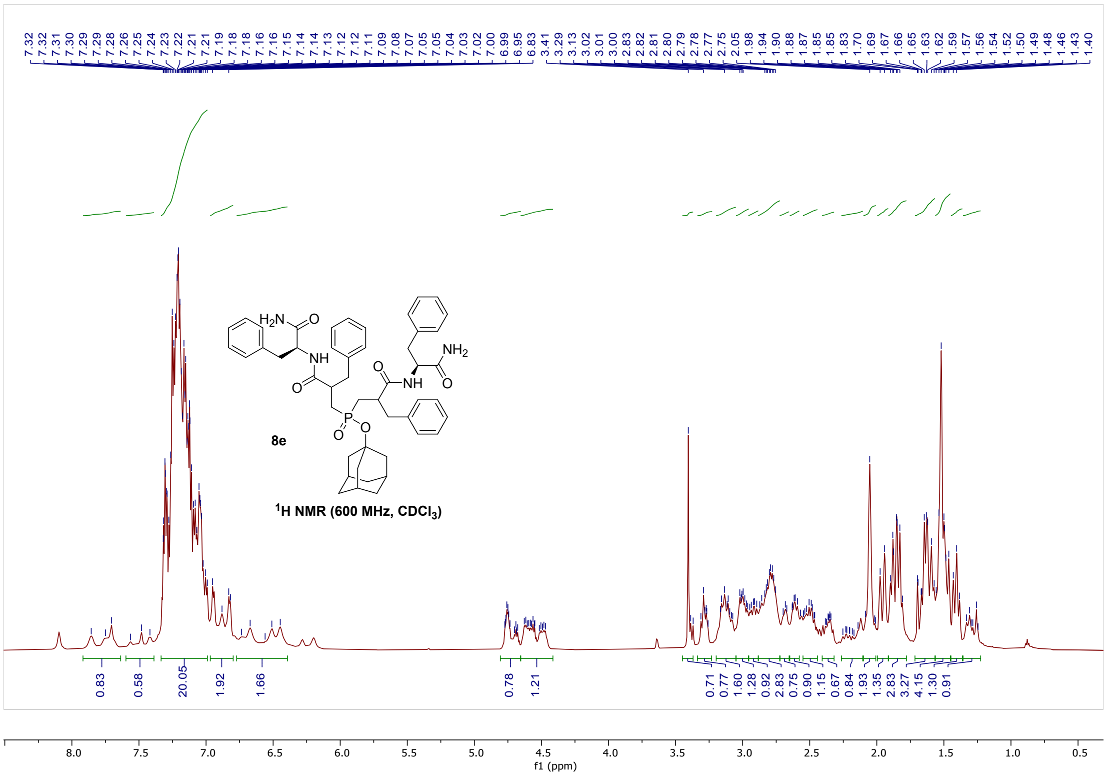

*Figure S26. ^{1}HNMR of Compound **8e***

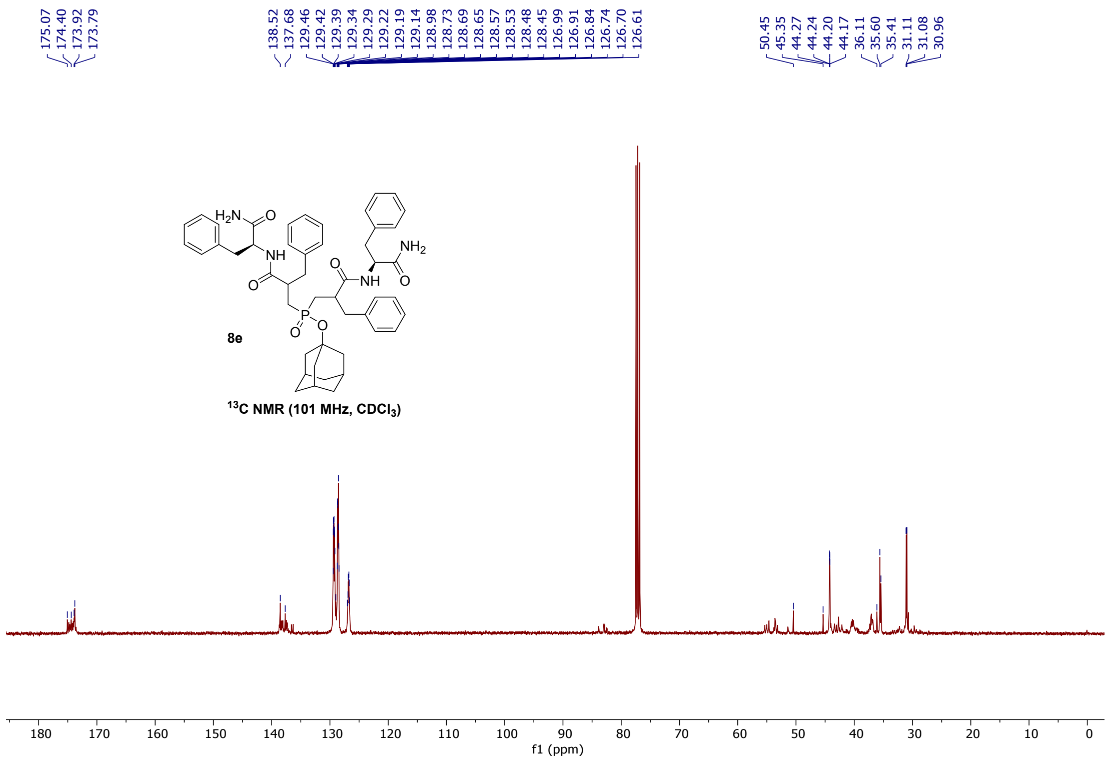

*Figure S27. ^{13}CNMR of Compound **8e***

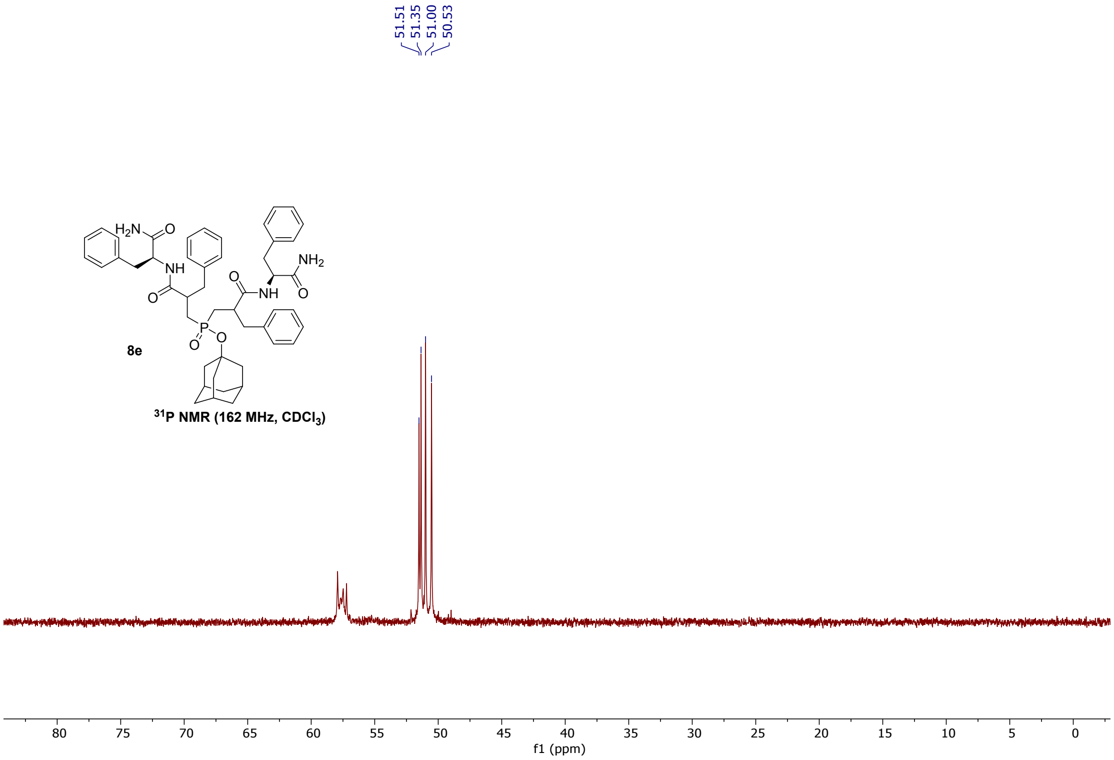

*Figure S28. ^{31}PNMR of Compound **8e***

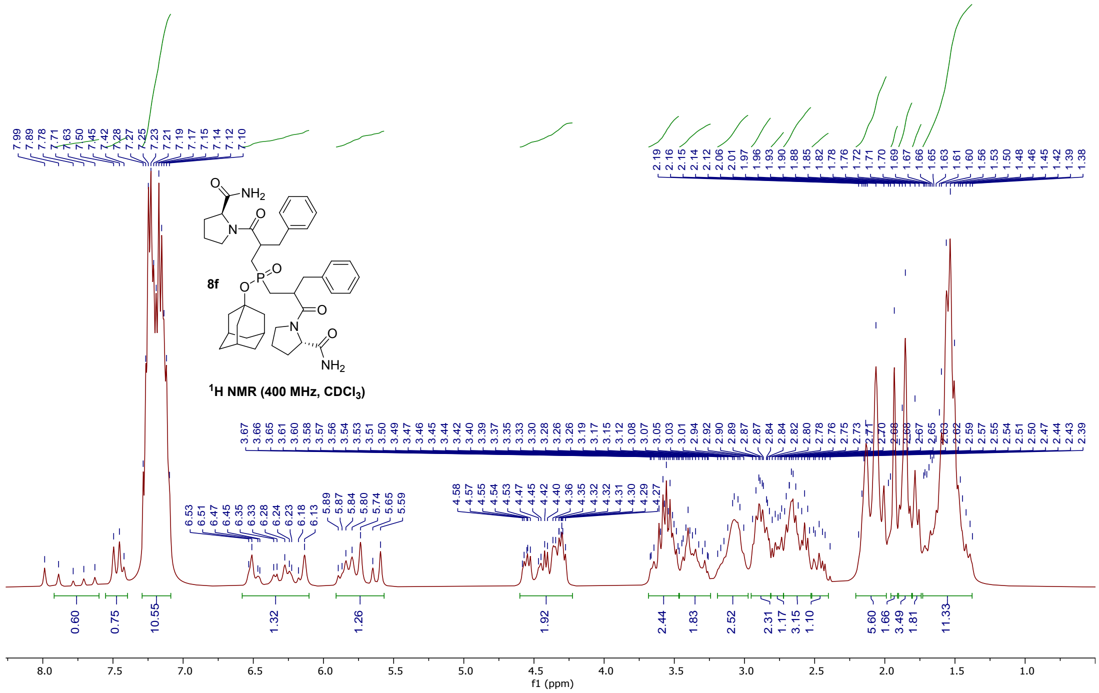

*Figure S29. ^{1}HNMR of Compound **8f***

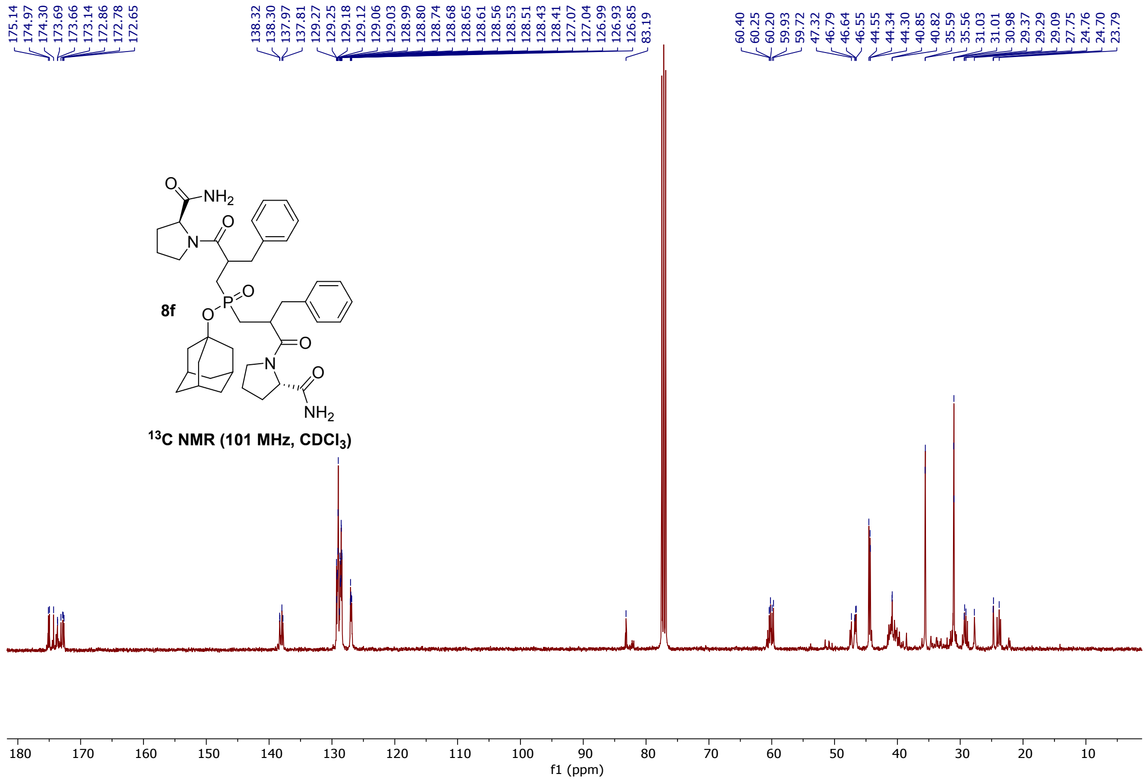

*Figure S30. ^{13}CNMR of Compound **8f***

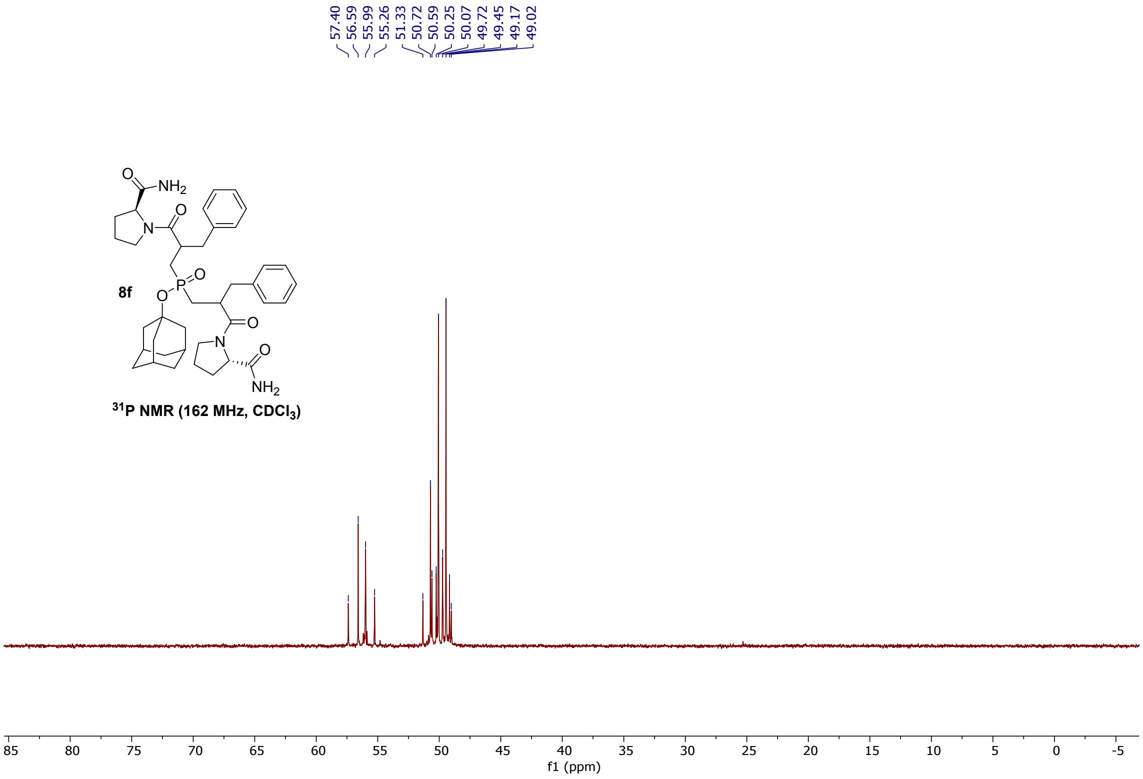

*Figure S31. ³¹PNMR of Compound **8f***

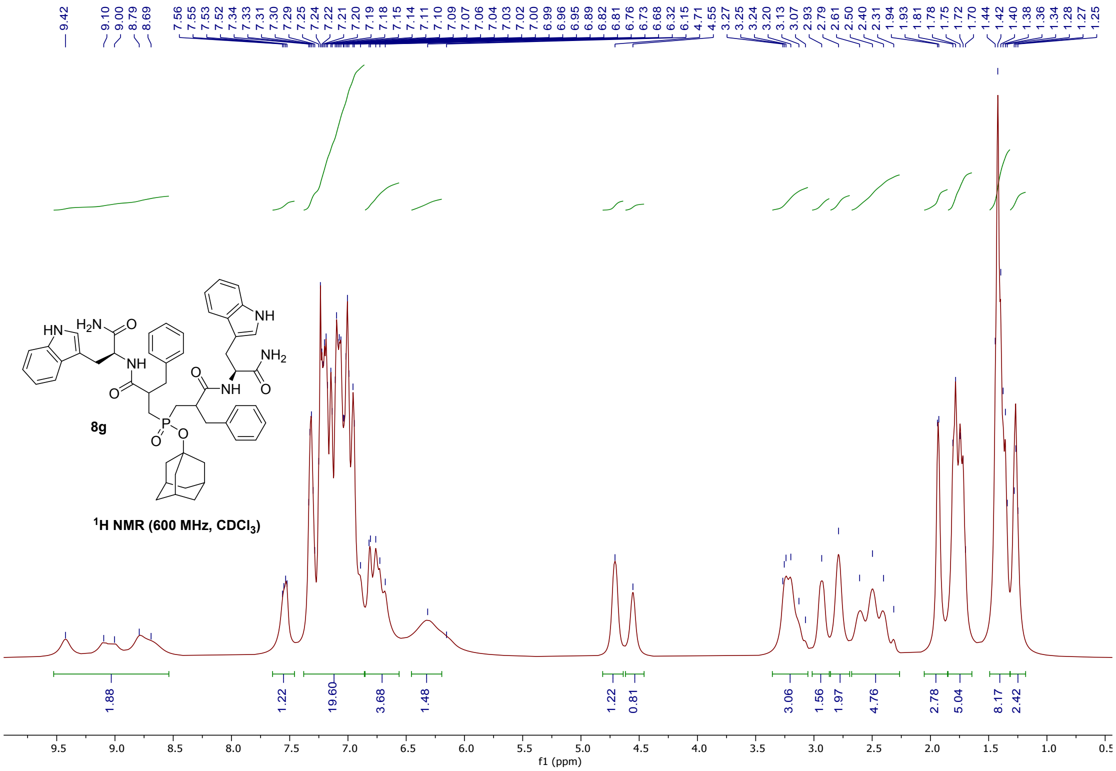

*Figure S32. ¹HNMR of Compound **8g***

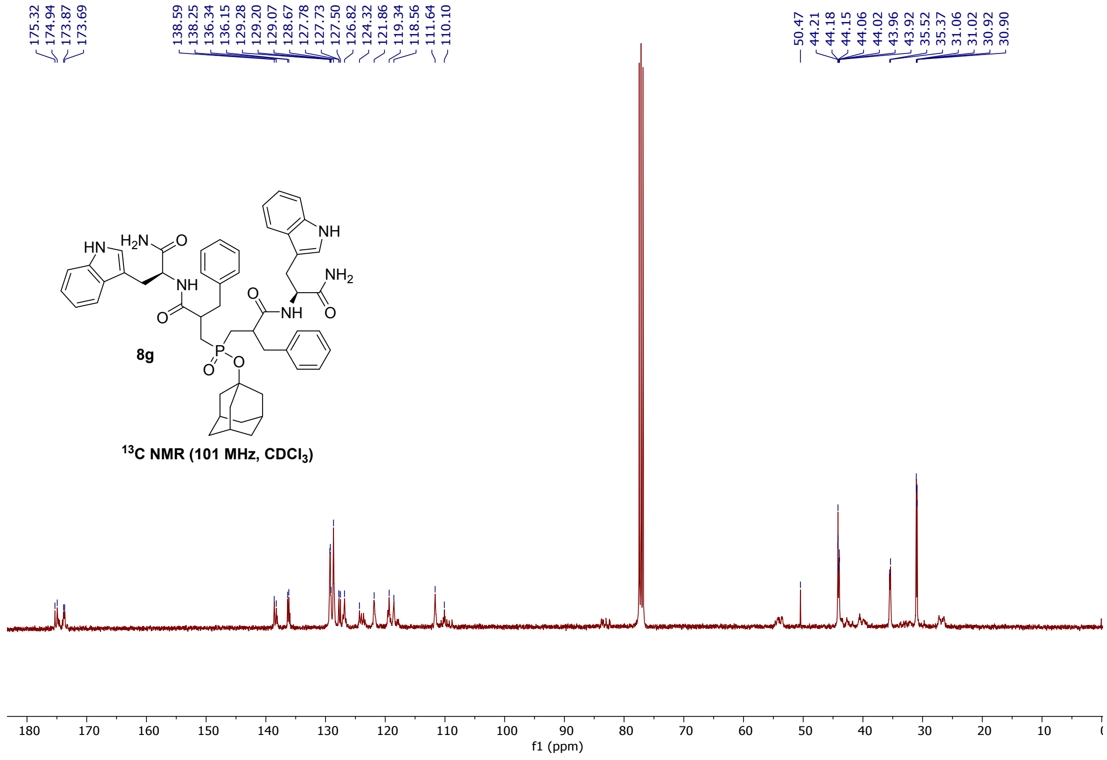

*Figure S33. ^{13}CNMR of Compound **8g***

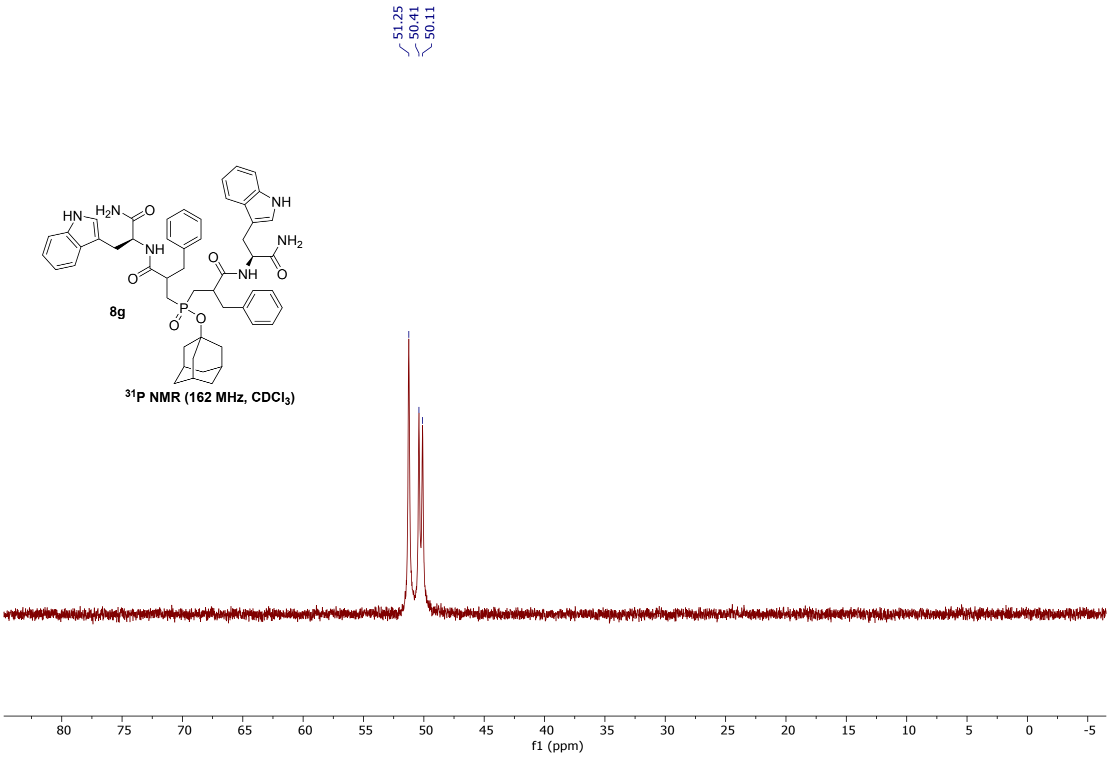

*Figure S34. ^{31}PNMR of Compound **8g***

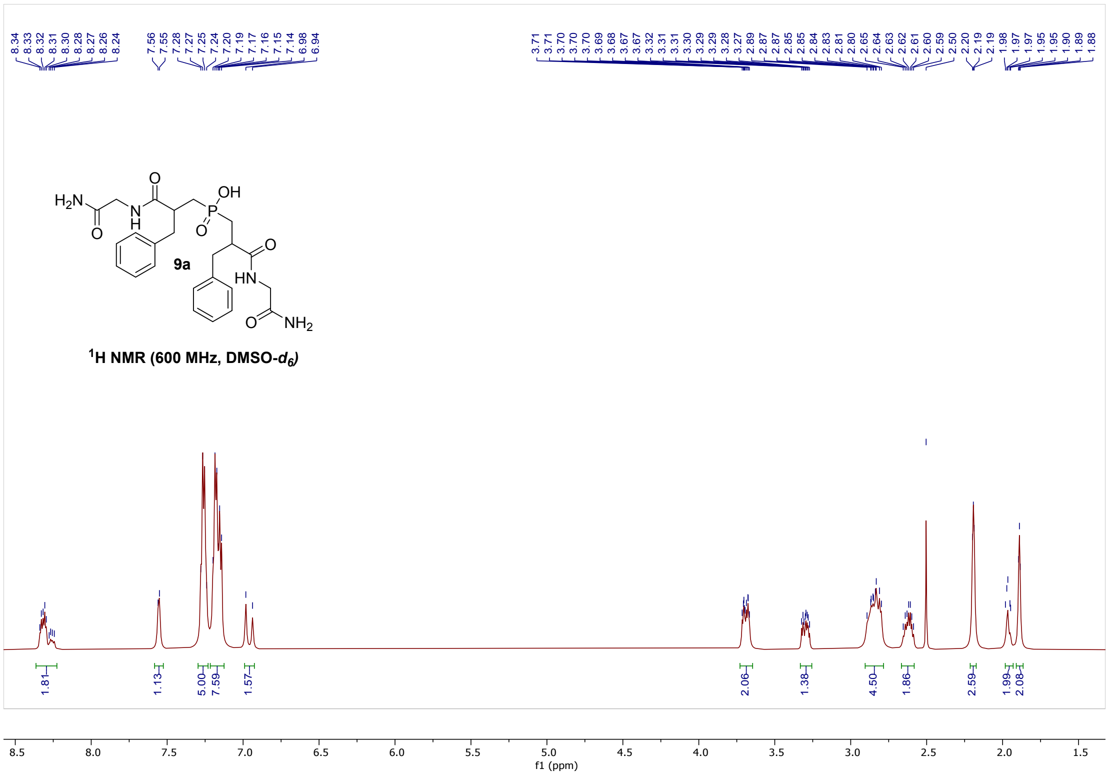

*Figure S35. ^{1}HNMR of Compound **9a***

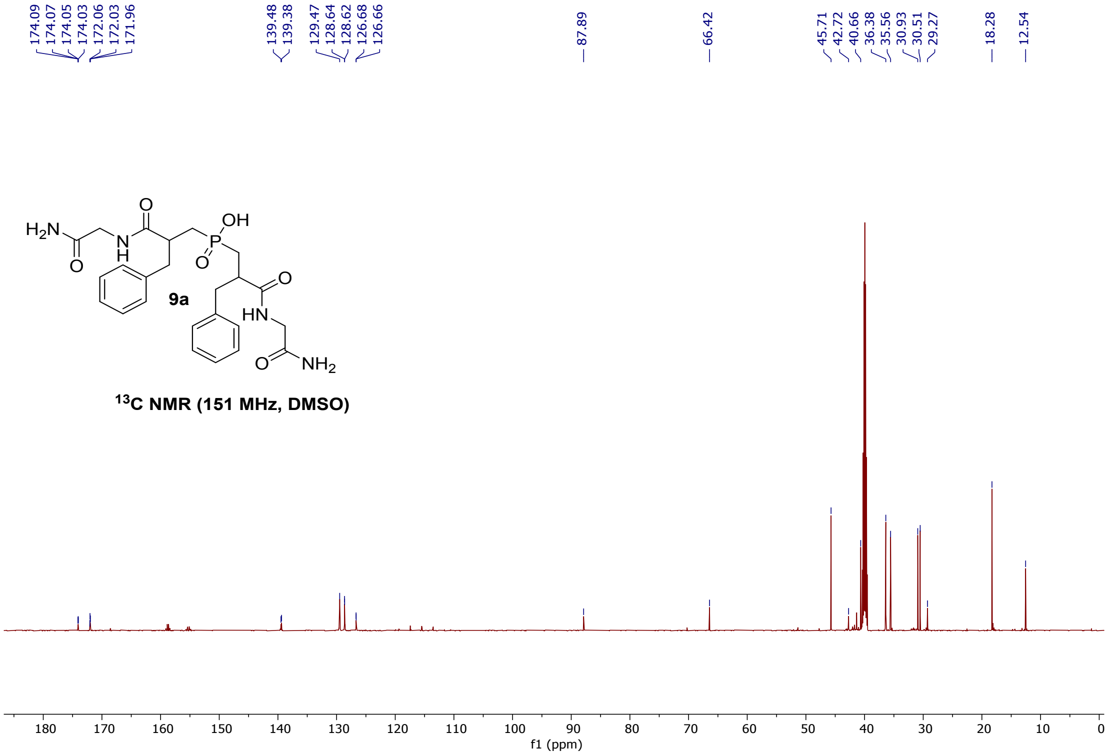

*Figure S36. ^{13}CNMR of Compound **9a***

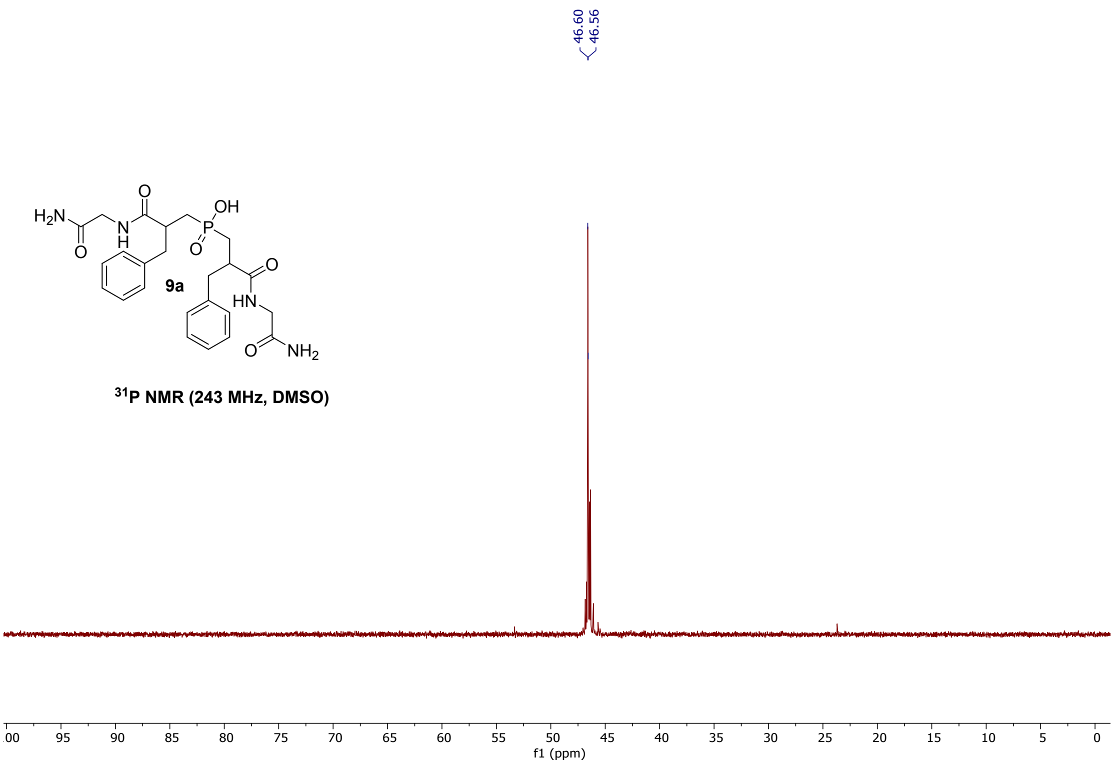

*Figure S37. ^{31}PNMR of Compound **9a***

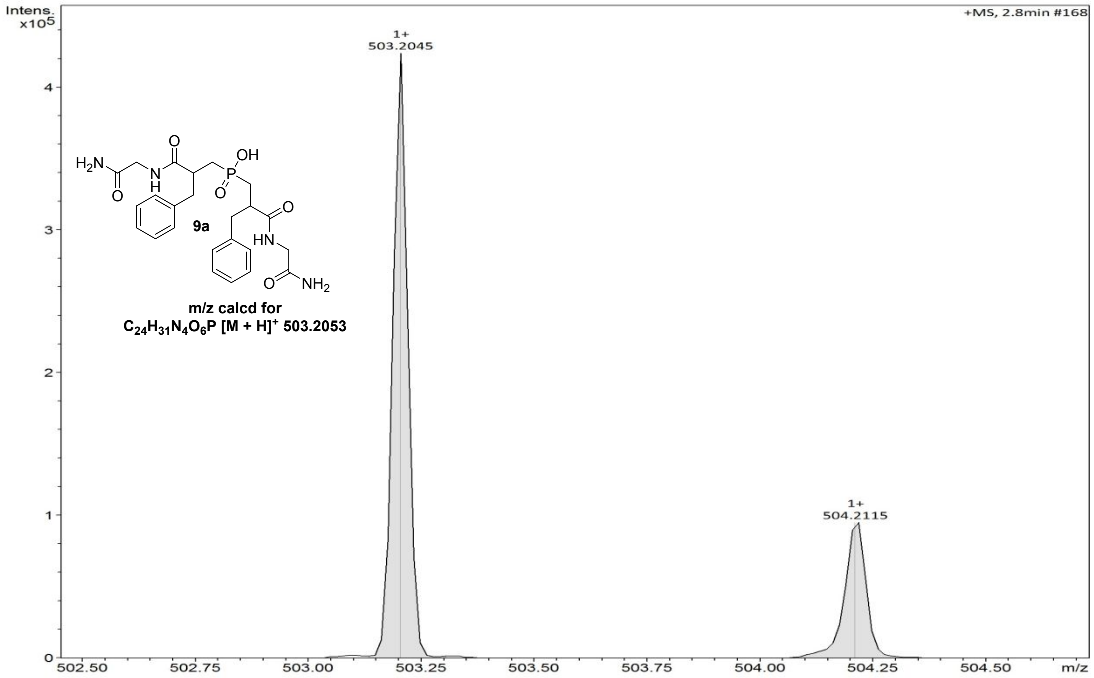

*Figure S38. HRMS Spectrum of Compound **9a***

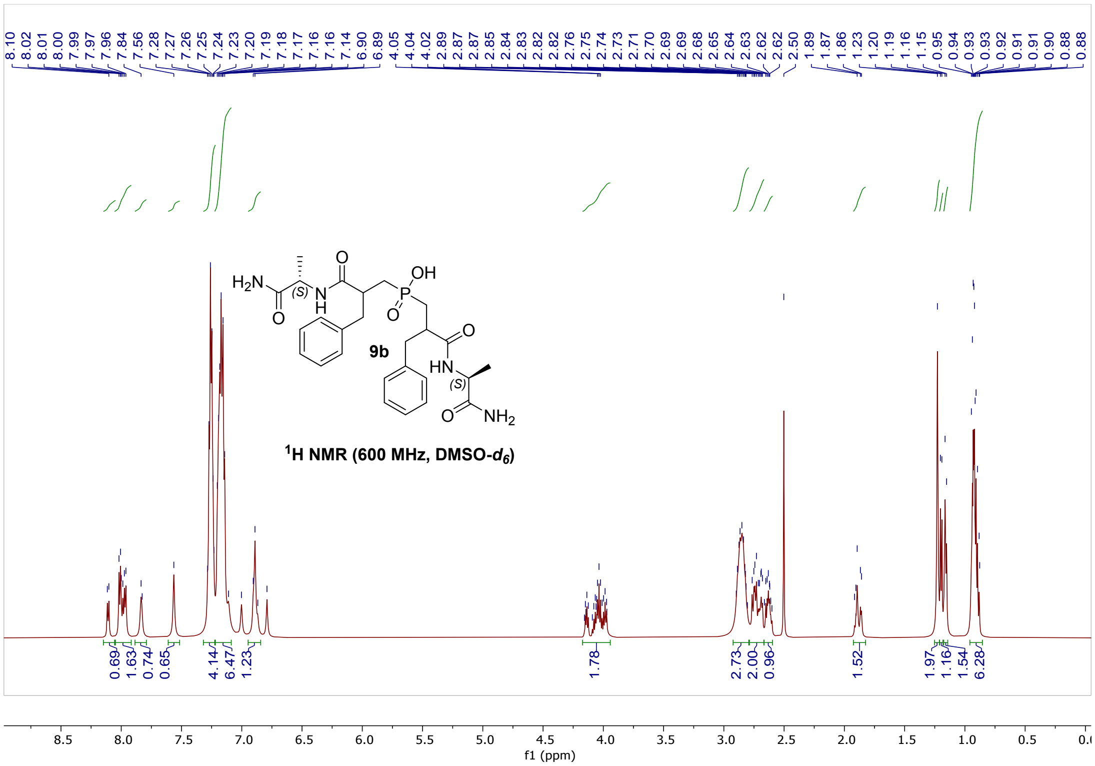

Figure S39. ^{1}HNMR of Compound ***9b***

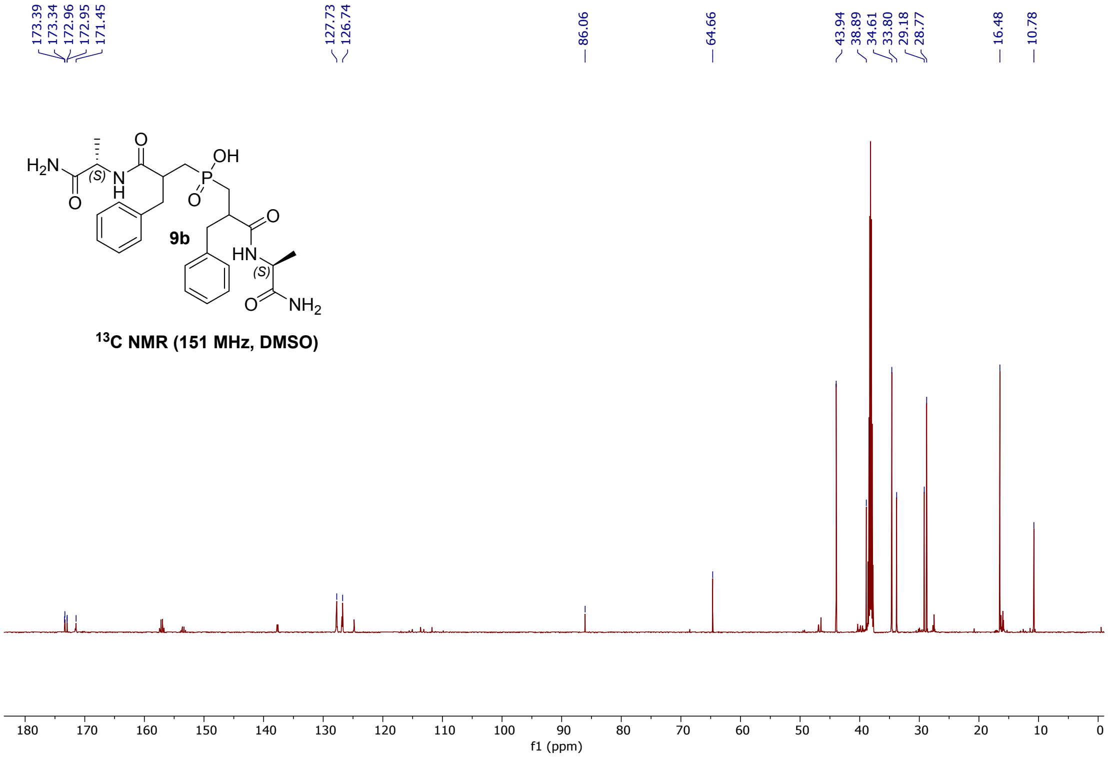

Figure S40. ^{13}CNMR of Compound ***9b***

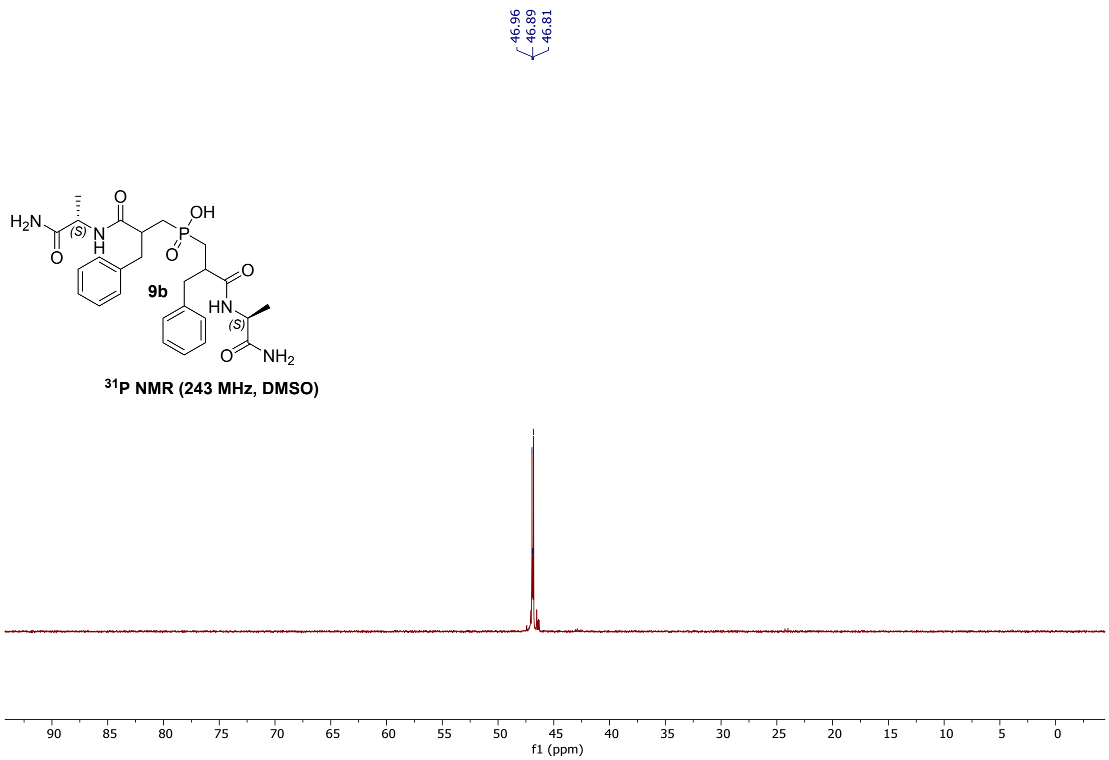

Figure S41. ^{31}PNMR of Compound ***9b***

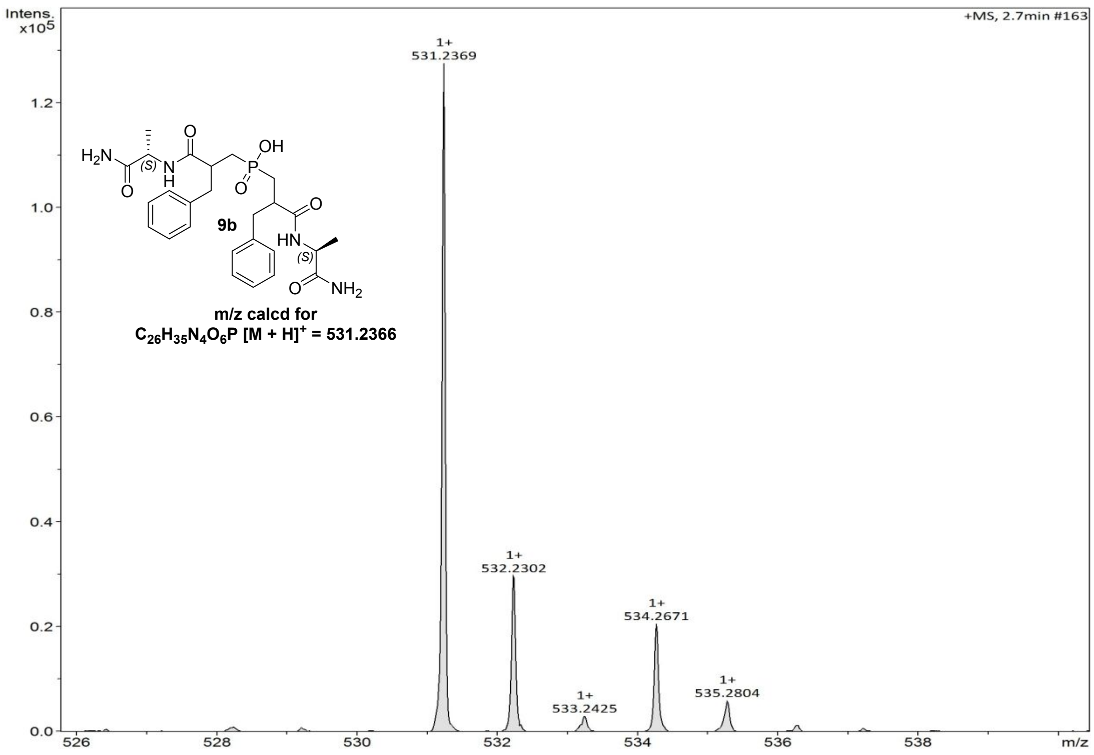

Figure S42. HRMS Spectrum of Compound ***9b***

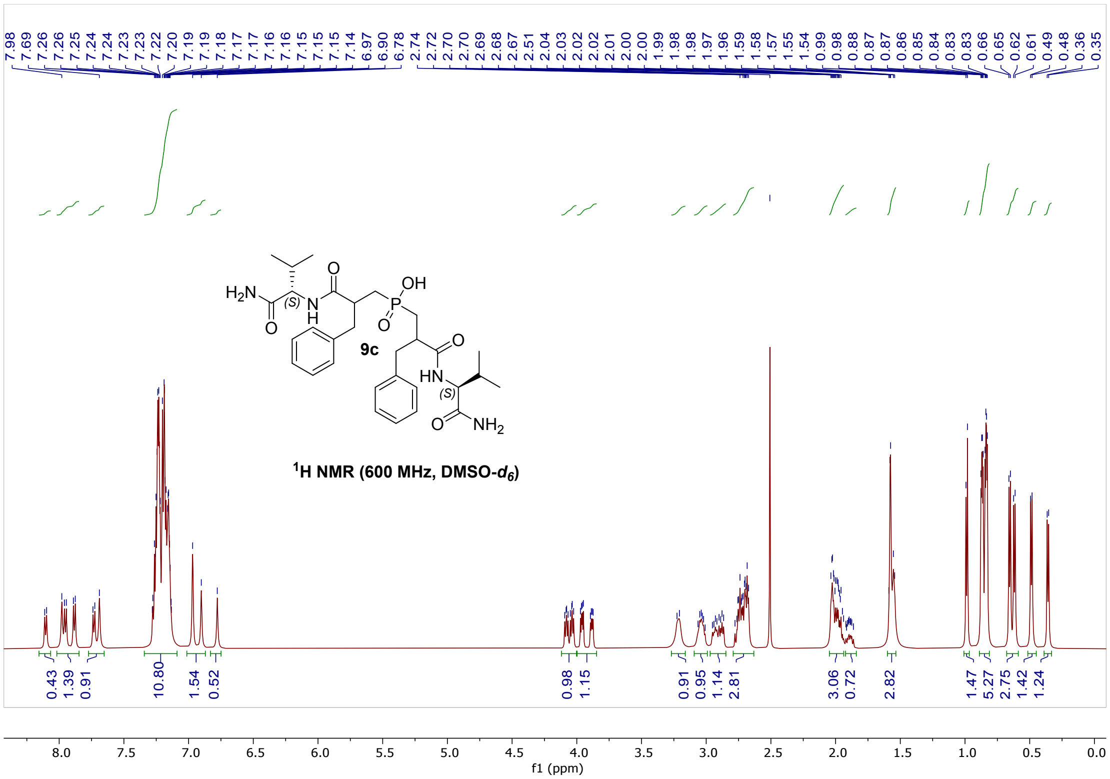

*Figure S43. ^{1}HNMR of Compound **9c***

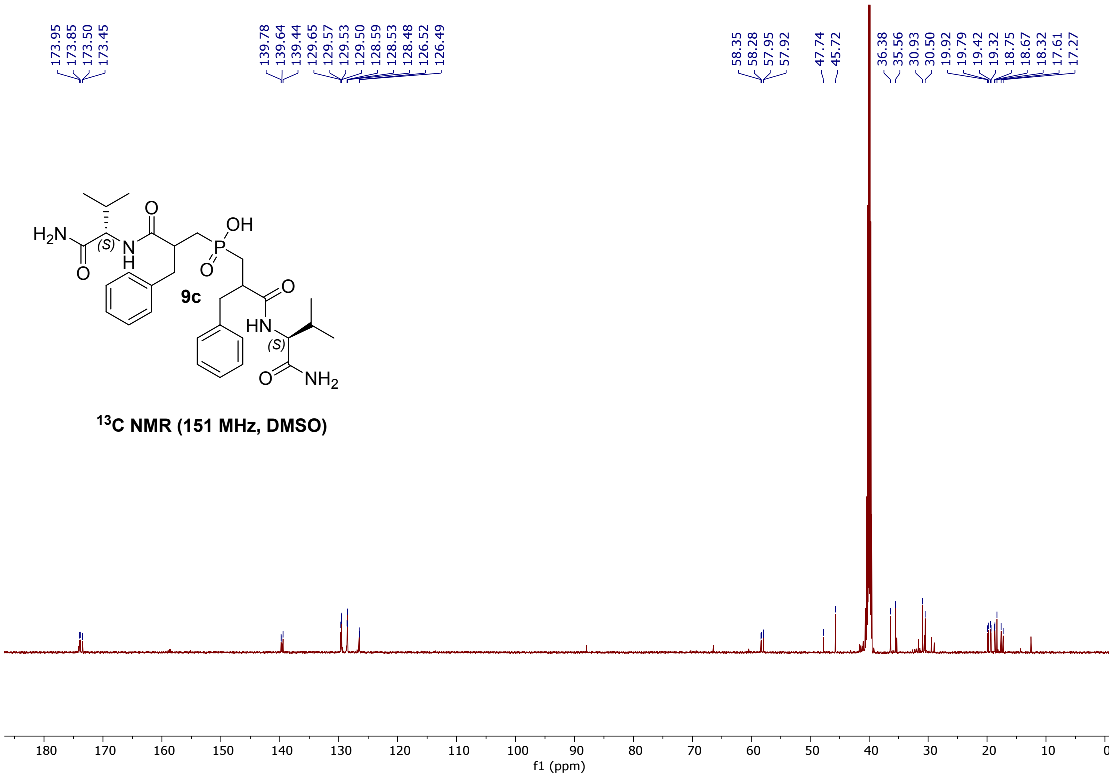

*Figure S44. ^{13}CNMR of Compound **9c***

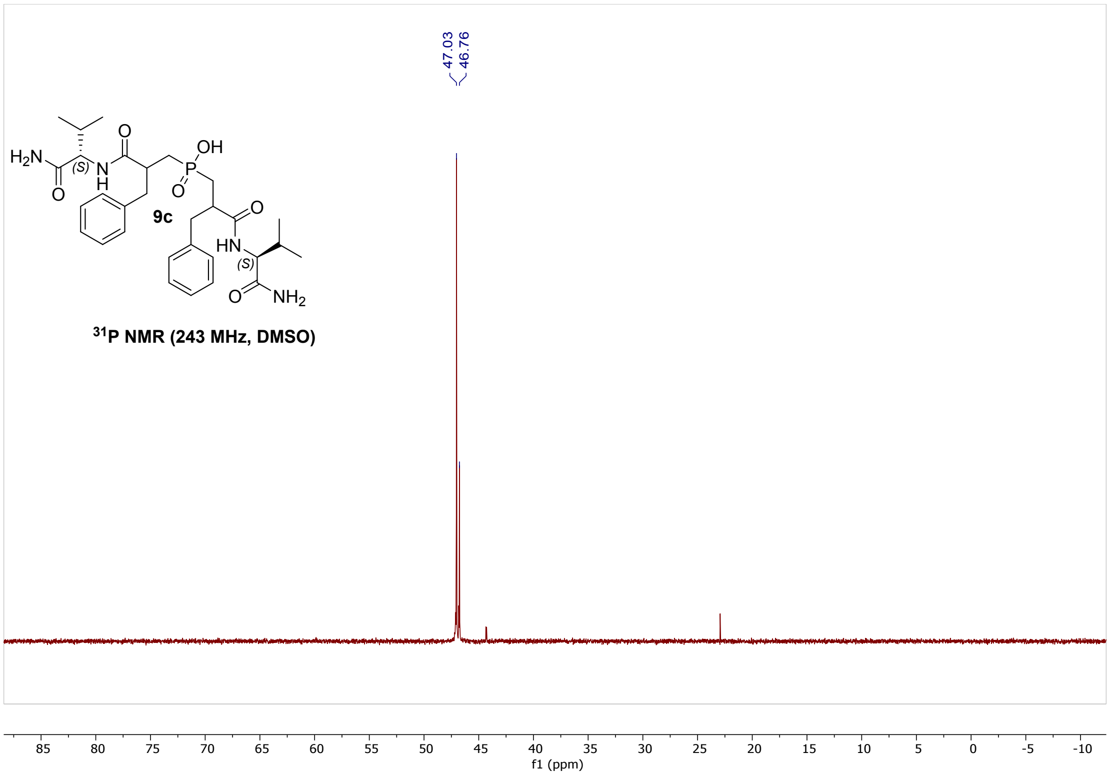

*Figure S45. ^{31}PNMR of Compound of **9c***

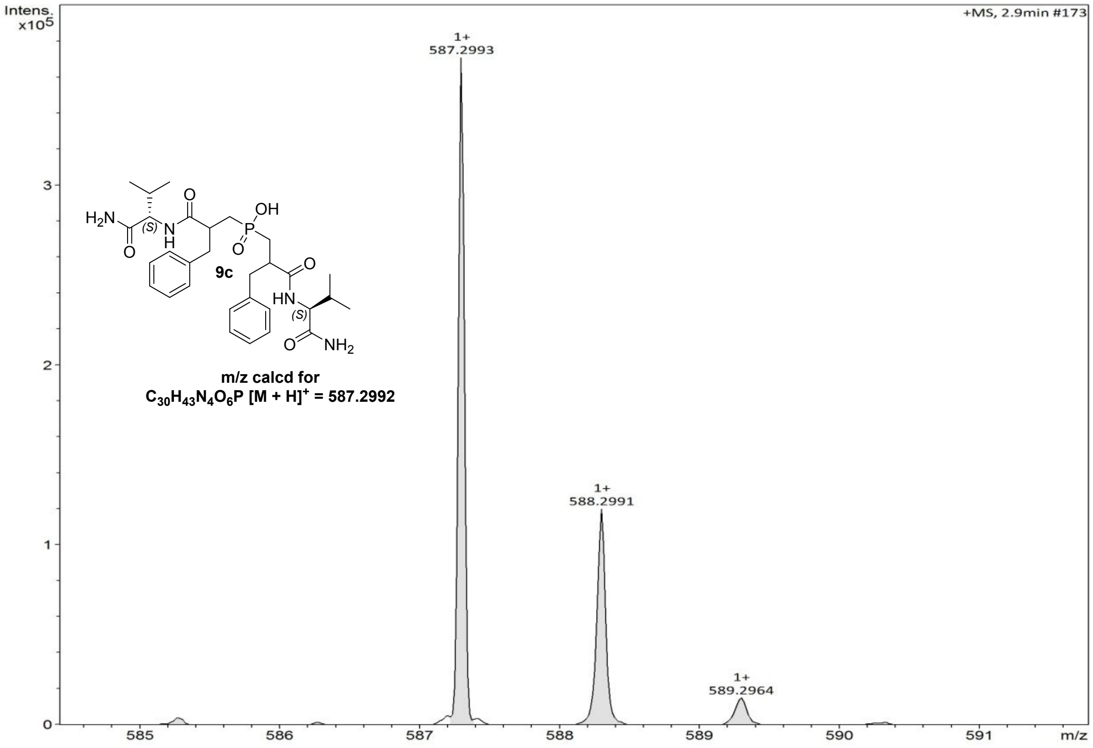

*Figure S46. HRMS Spectrum of Compound **9c***

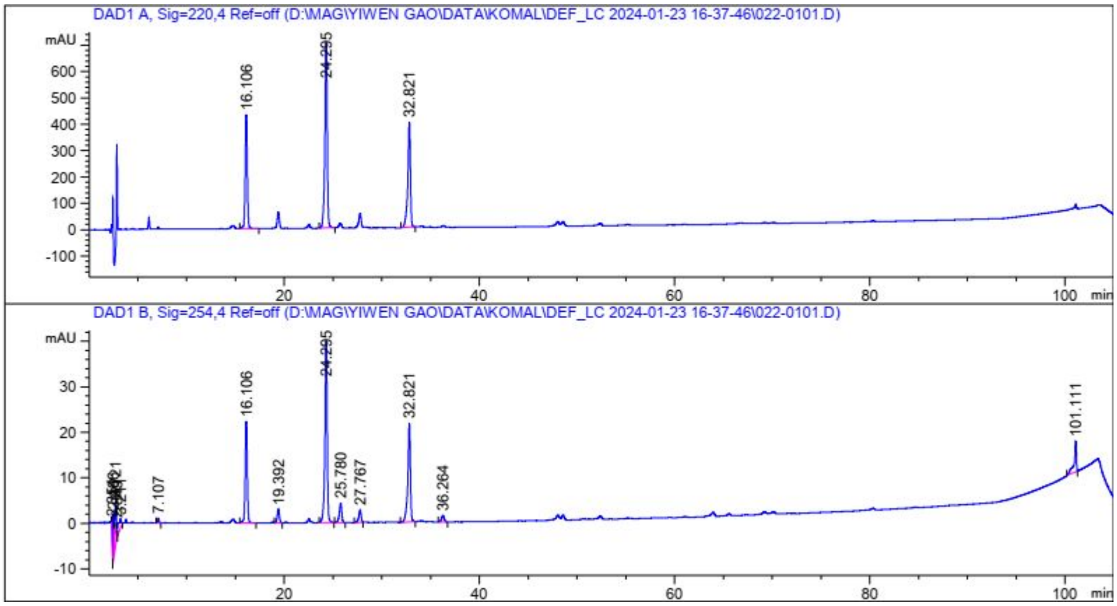

*Figure S47. HPLC chromatogram of Compound **9c***

Method Info :Mobile Phase :A:Water(0.1%TFA) B:ACN
Gradient:20%B for0.2 min,increase to90%B within6.8 min,90%B for8min,back to20%B within 0.1 min,20%B for5min
Flow Rate:1.0ml/min
Oven Temperature:40°C
Column:Shim-pack PREP-ODS(H)KIT 4.6*250mm,5um

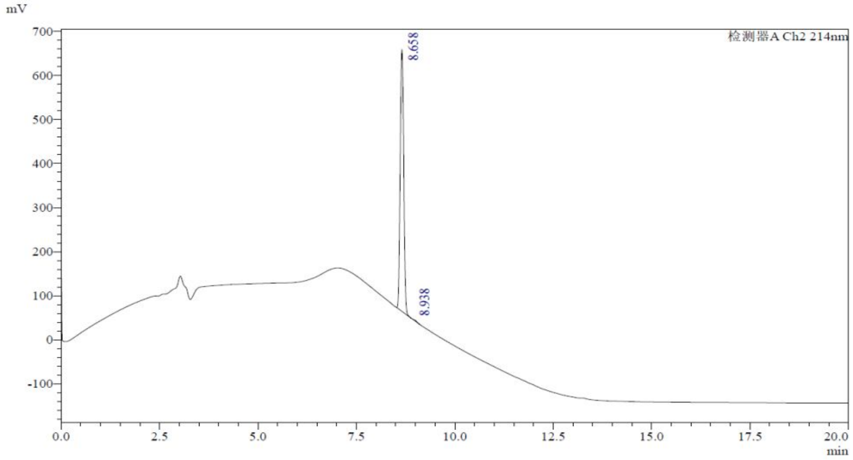

*Figure S48.HPLC chromatogram of Isomer **A** (**9c**)*

Method Info :Mobile Phase :A:Water(0.1%TFA) B:ACN
Gradient:20%B for0.2 min,increase to90%B within6.8 min,90%B for8min,back to20%B within 0.1 min,20%B for5min
Flow Rate:1.0ml/min
Oven Temperature:40°C
Column:Shim-pack PREP-ODS(H)KIT 4.6*250mm,5um

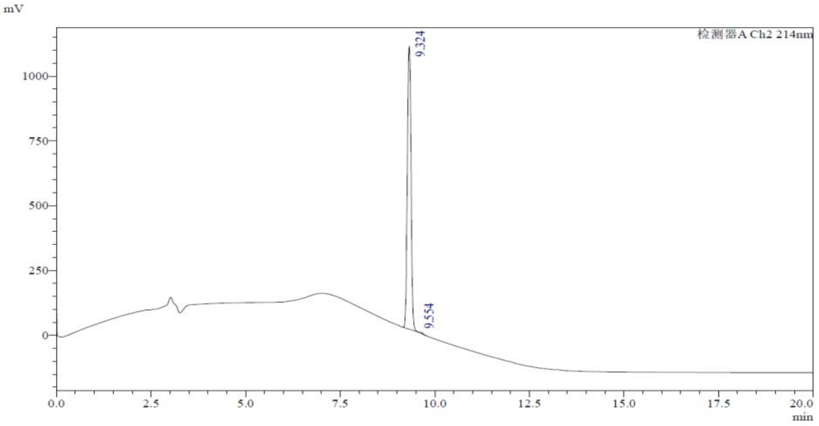

*Figure S49. HPLC chromatogram of Isomer **B** (**9c**)*

Method Info :Mobile Phase :A:Water(0.1%TFA) B:ACN
Gradient:20%B for0.2 min,increase to90%B within6.8 min,90%B for8min,back to20%B within 0.1 min,20%B for5min
Flow Rate:1.0ml/min
Oven Temperature:40°C
Column:Shim-pack PREP-ODS(H)KIT 4.6*250mm,5um

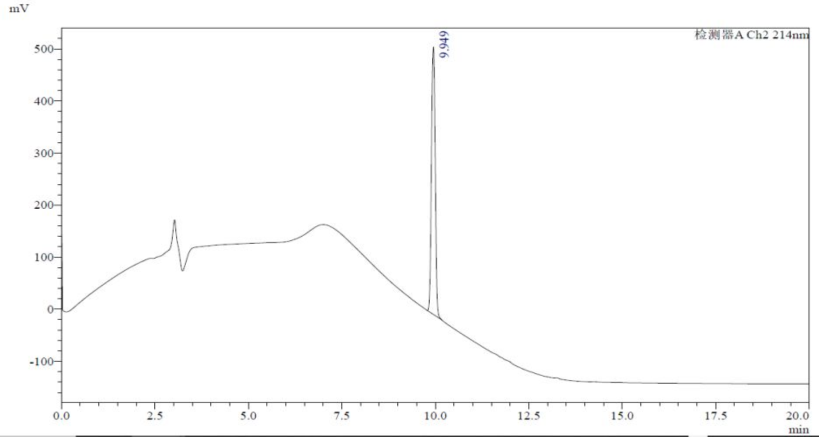

*Figure S50. HPLC chromatogram of Isomer **C** (**9c**)*

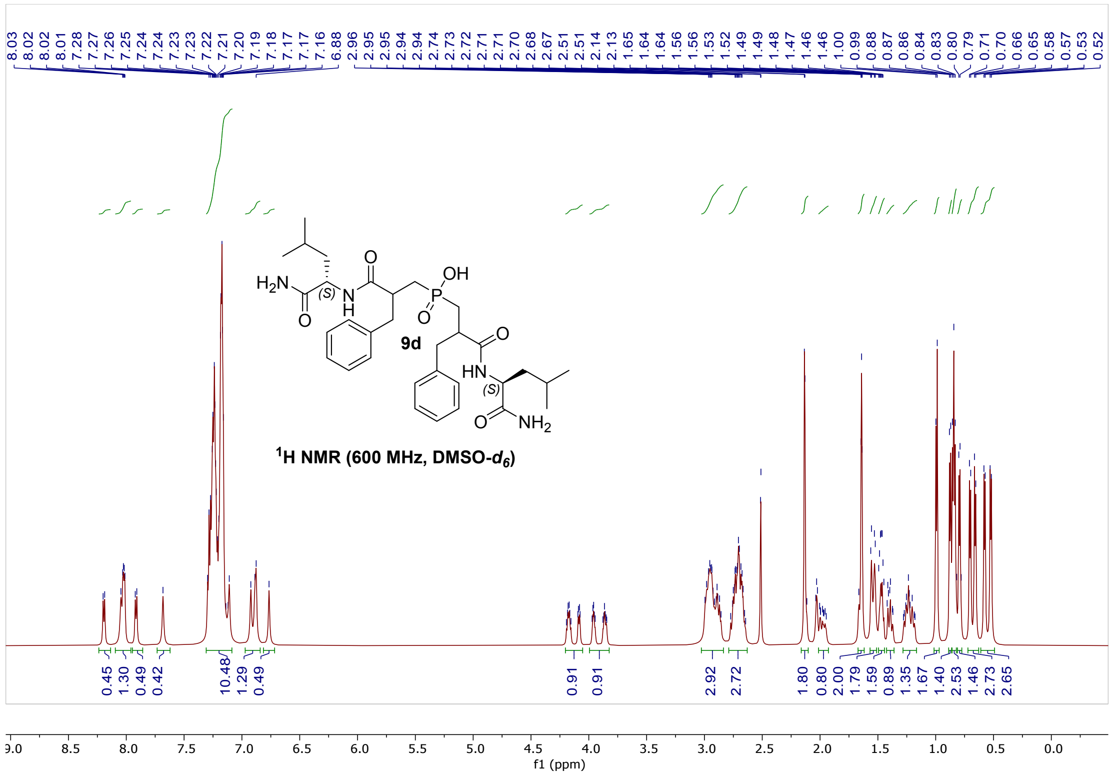

*Figure S51. ^{1}HNMR of Compound **9d***

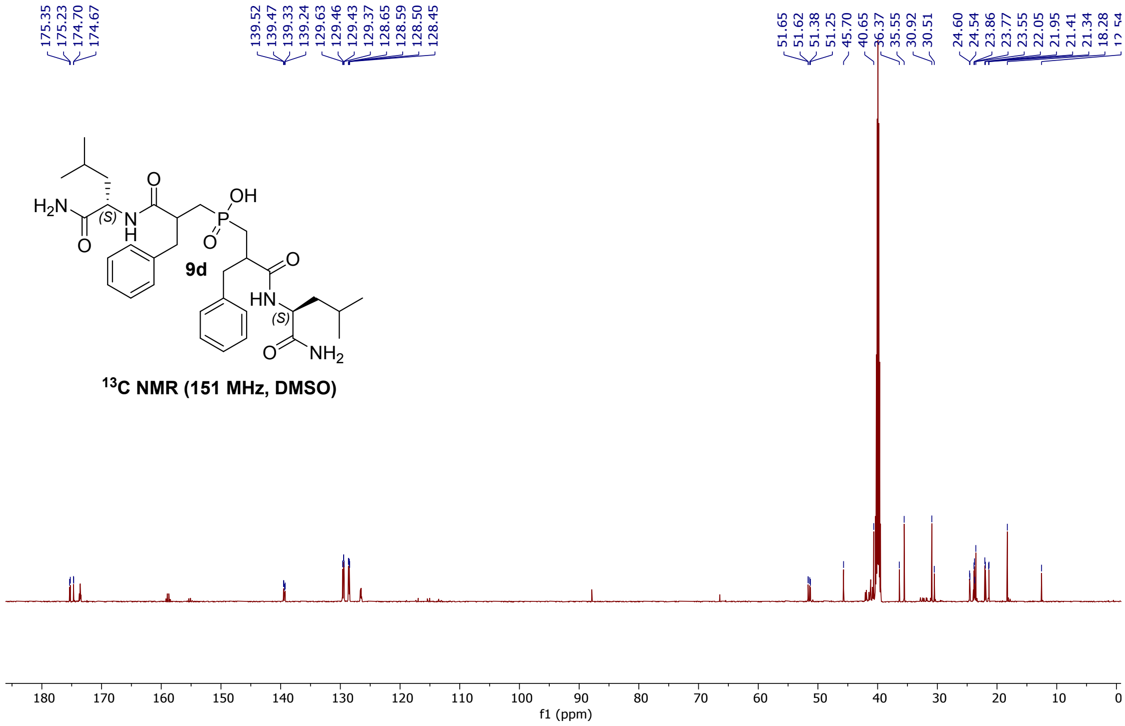

*Figure S52. ^{13}CNMR of Compound **9d***

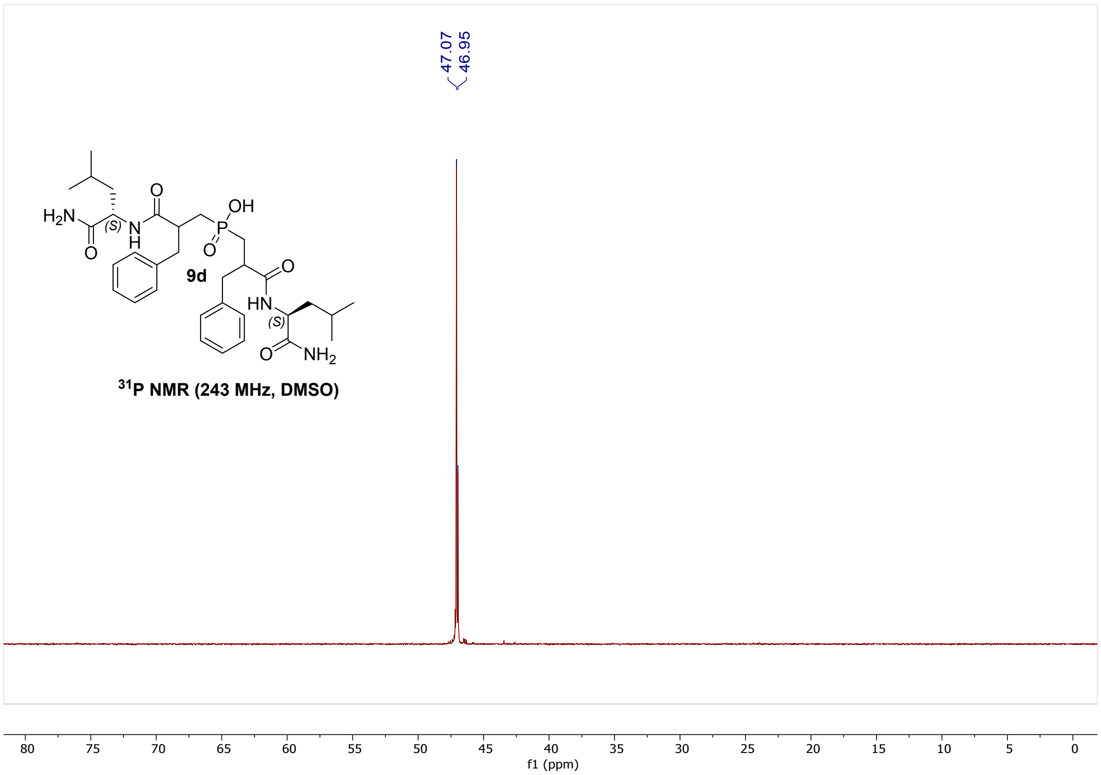

*Figure S53. [31]PMNR of Compound **9d***

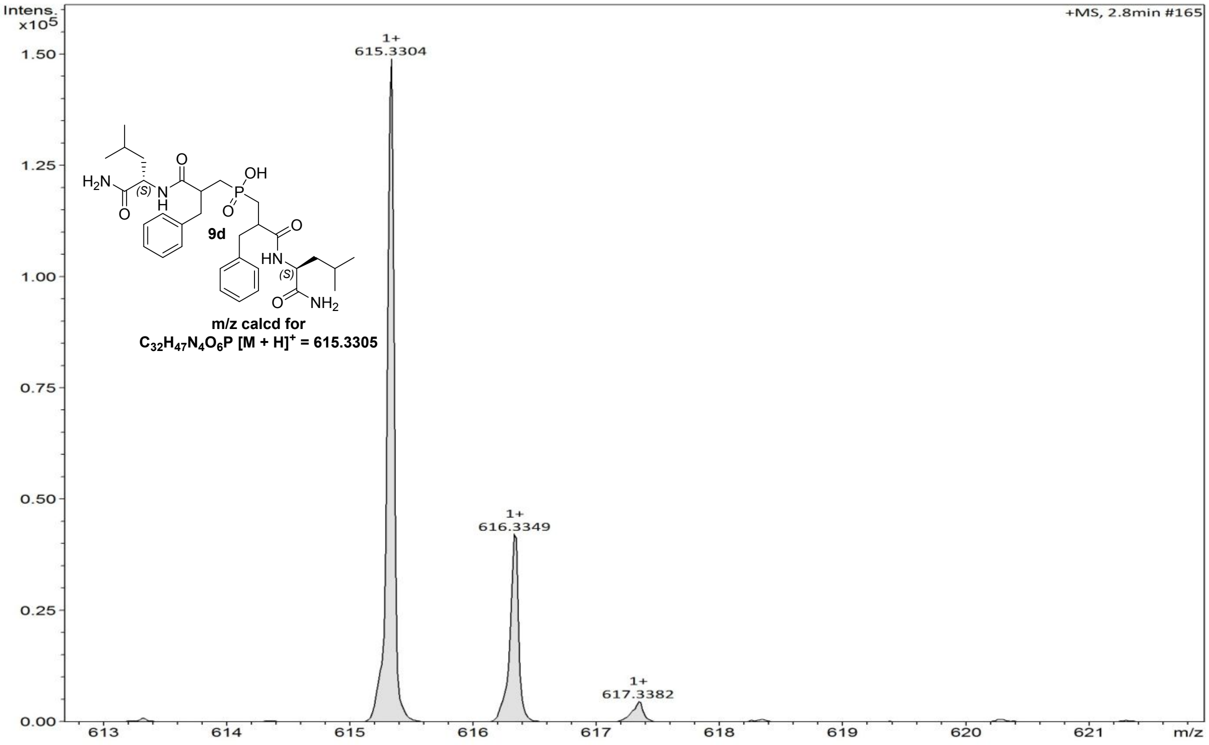

*Figure S54. HRMS Spectrum of Compound **9d***

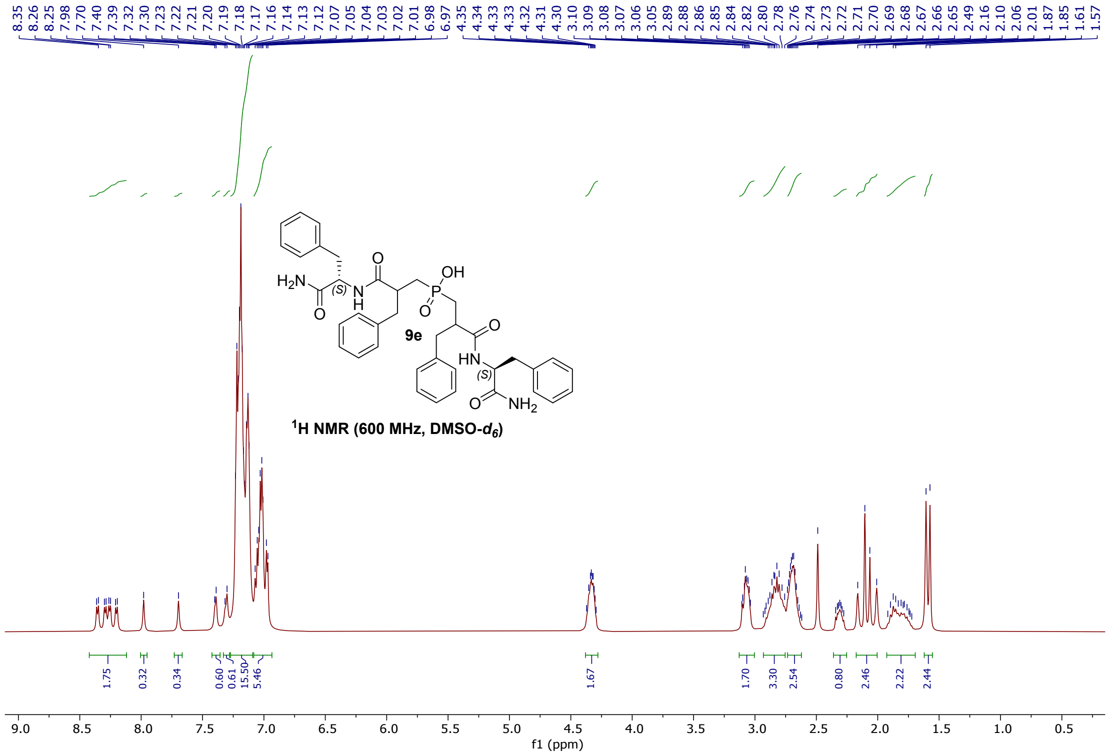

Figure S55. ^{1}*HNMR of Compound* ***9e***

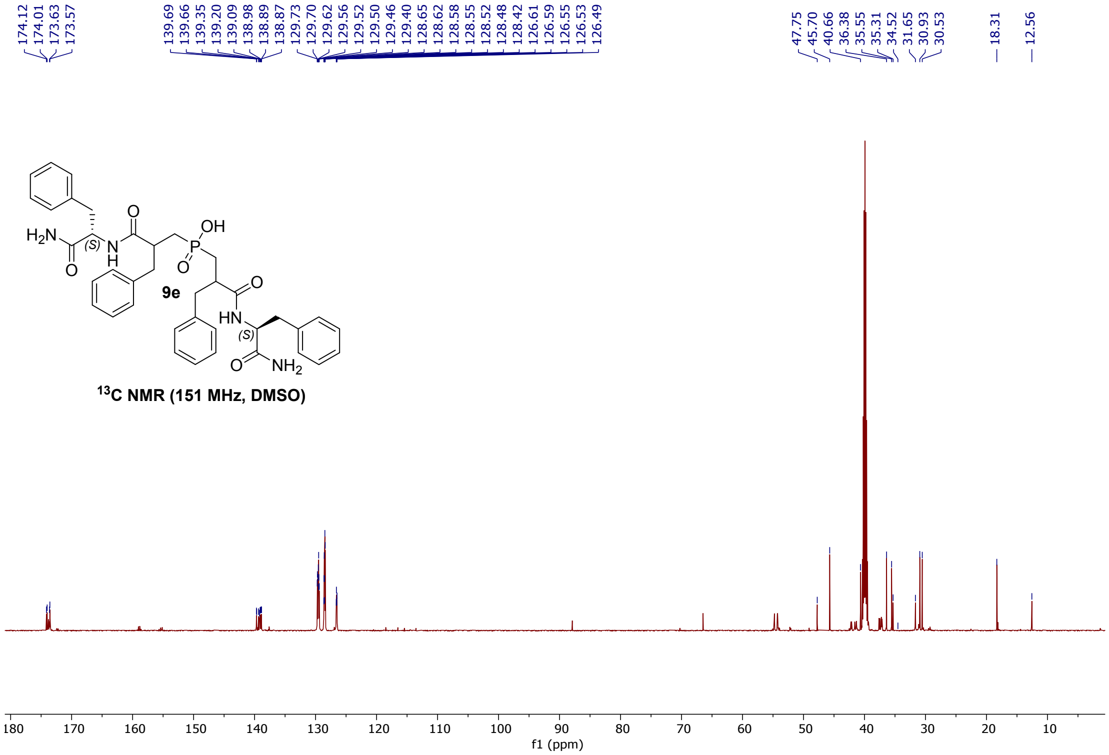

Figure S56. ^{13}*CNMR of Compound* ***9e***

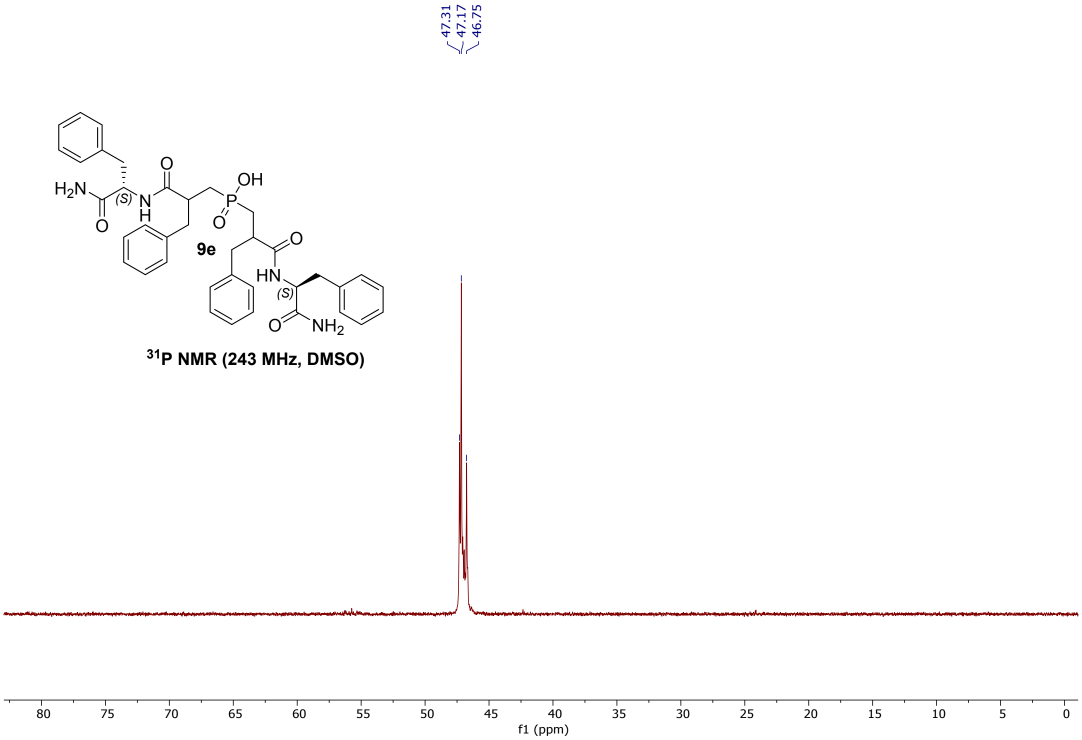

*Figure S57. ^{31}PNMR of Compound **9e***

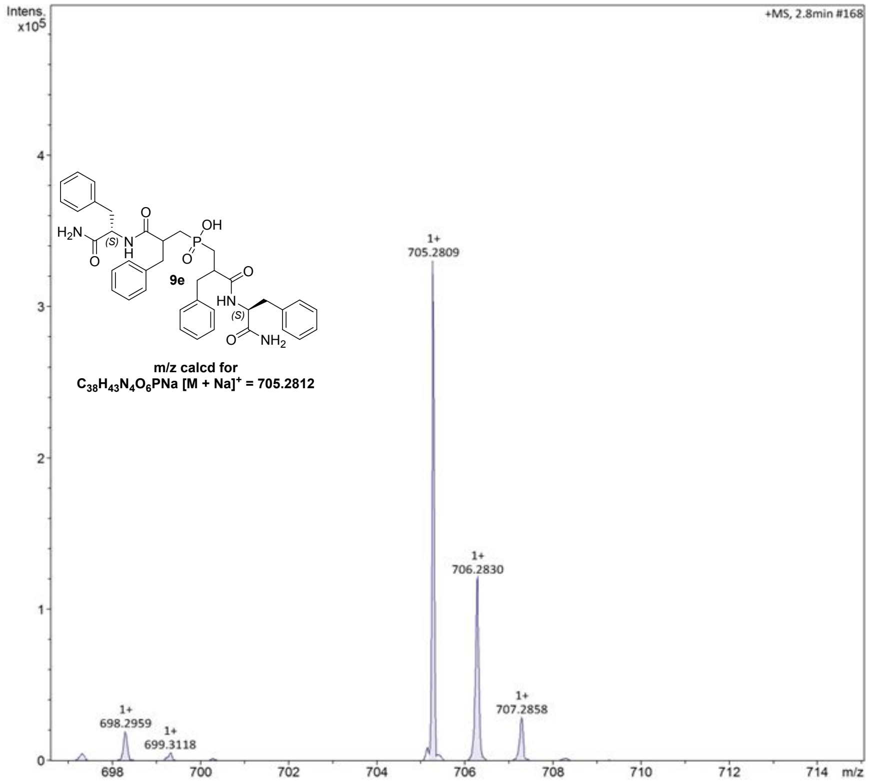

*Figure S58. HRMS Spectrum of Compound **9e***

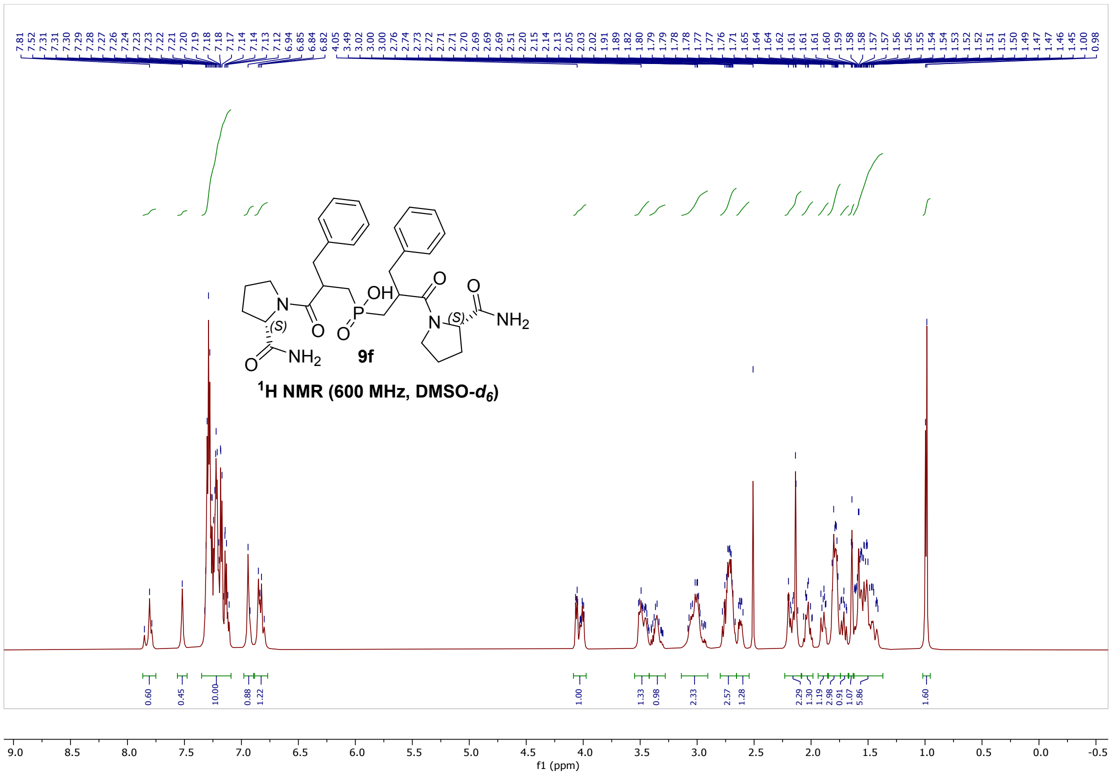

*Figure S59. ^{1}HNMR of Compound **9f***

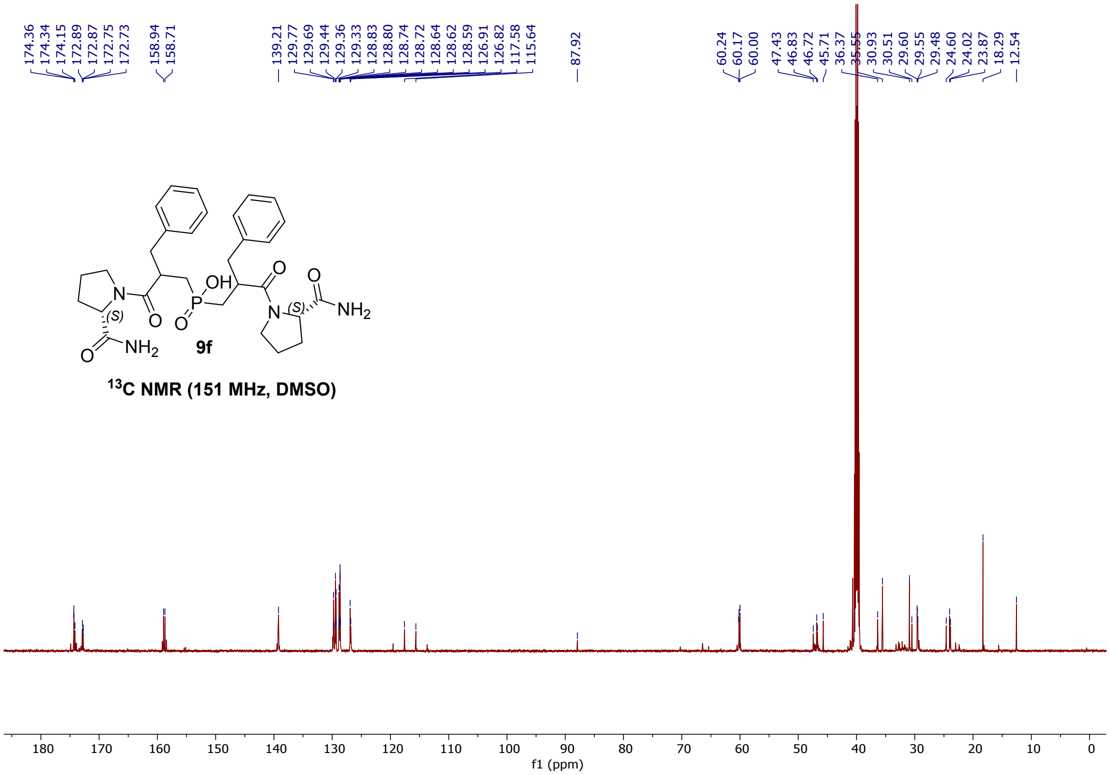

*Figure S60. ^{13}CNMR of Compound **9f***

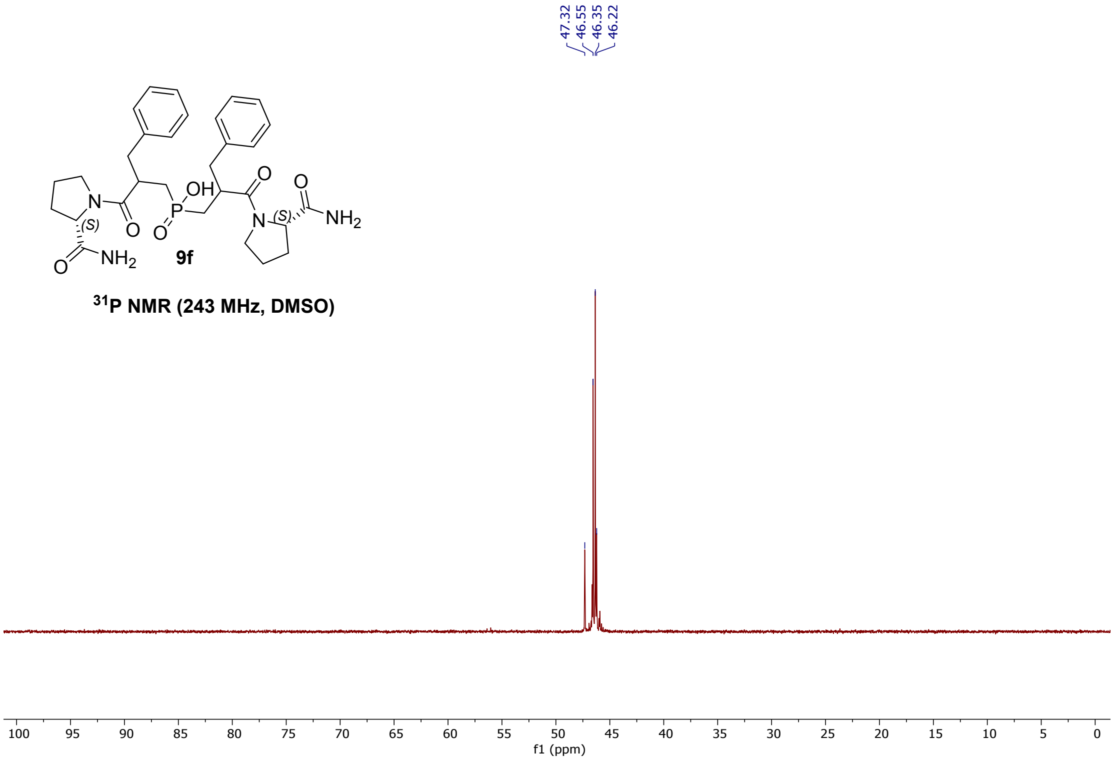

*Figure S61. ^{31}PNMR of Compound **9f***

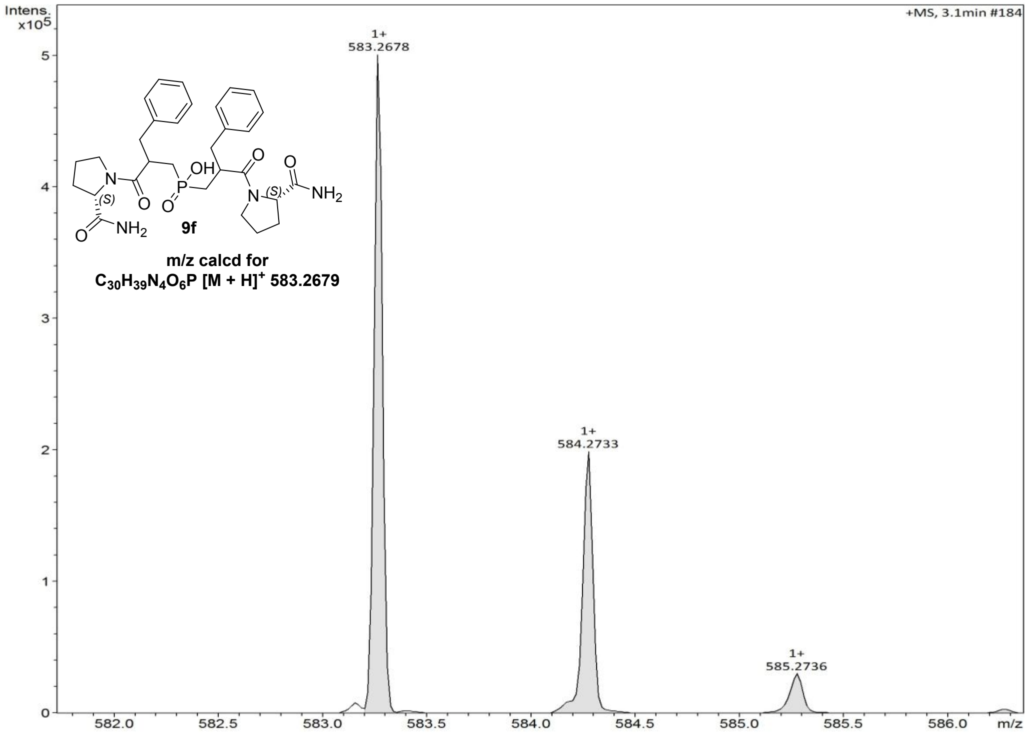

*Figure S62. HRMS Spectrum of Compound **9f***

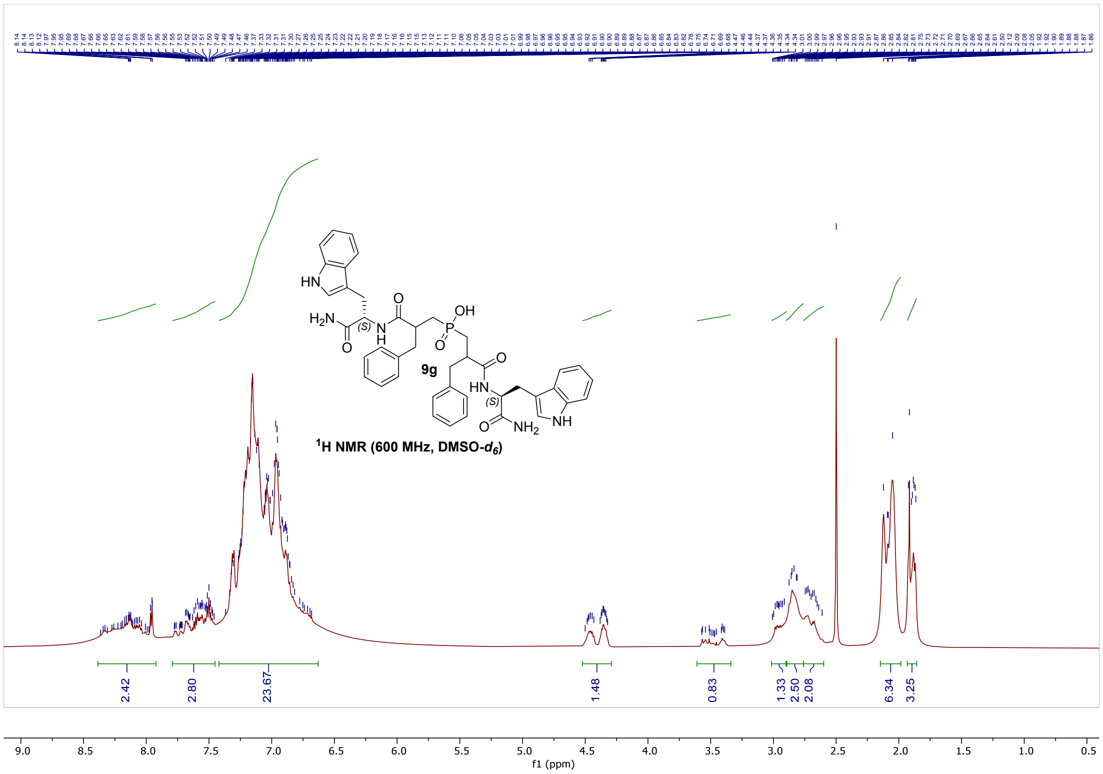

*Figure S63. ^{1}HNMR of Compound **9g***

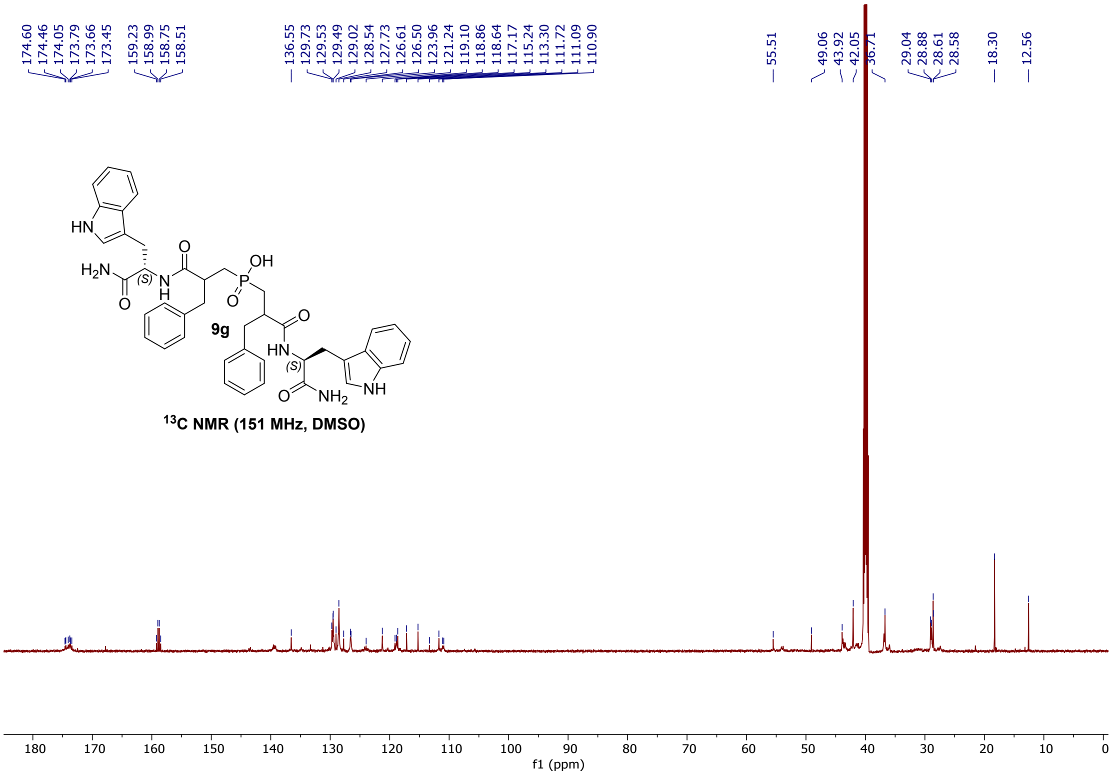

*Figure S64. ^{13}CNMR of Compound **9g***

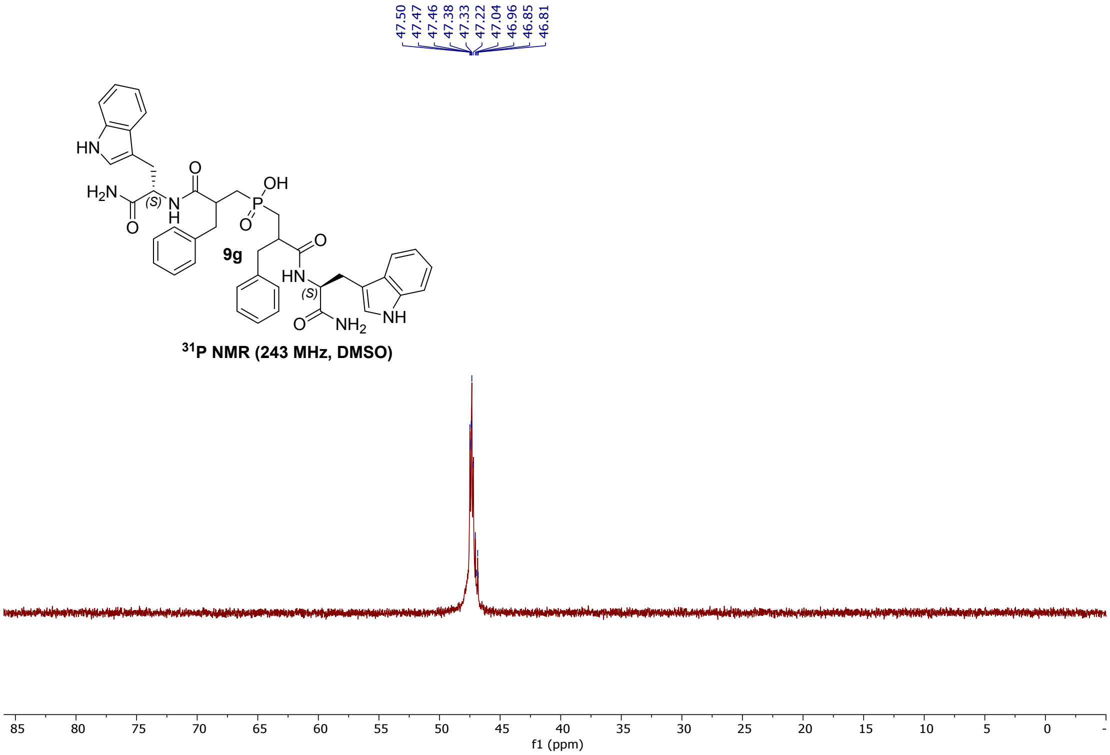

*Figure S65. ^{31}PNMR of Compound **9g***

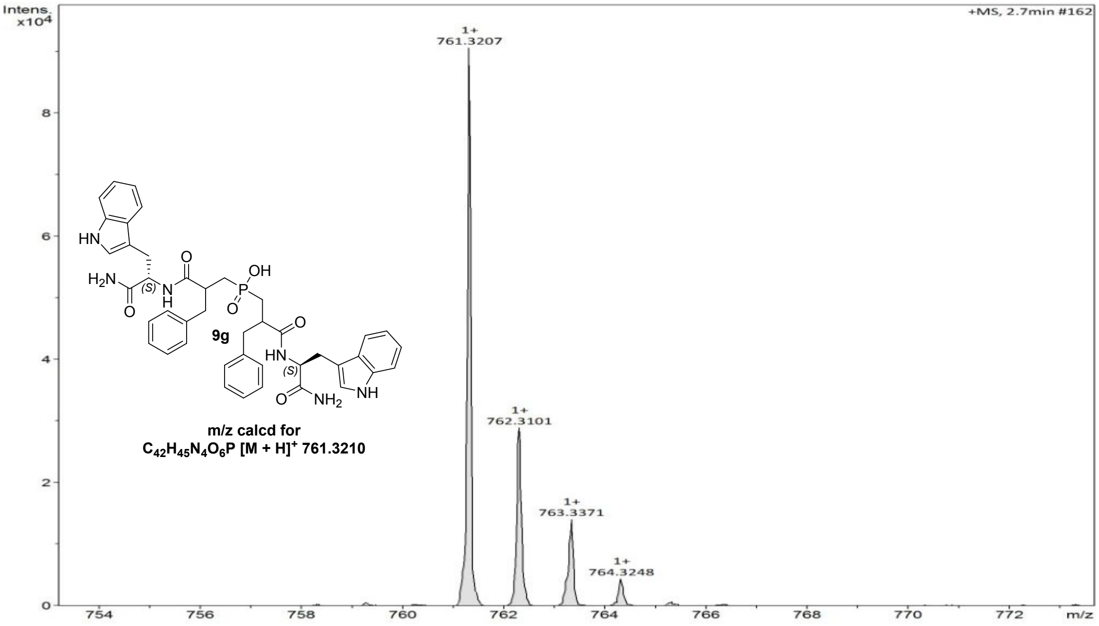

*Figure S66. HRMS Spectrum of Compound **9g***

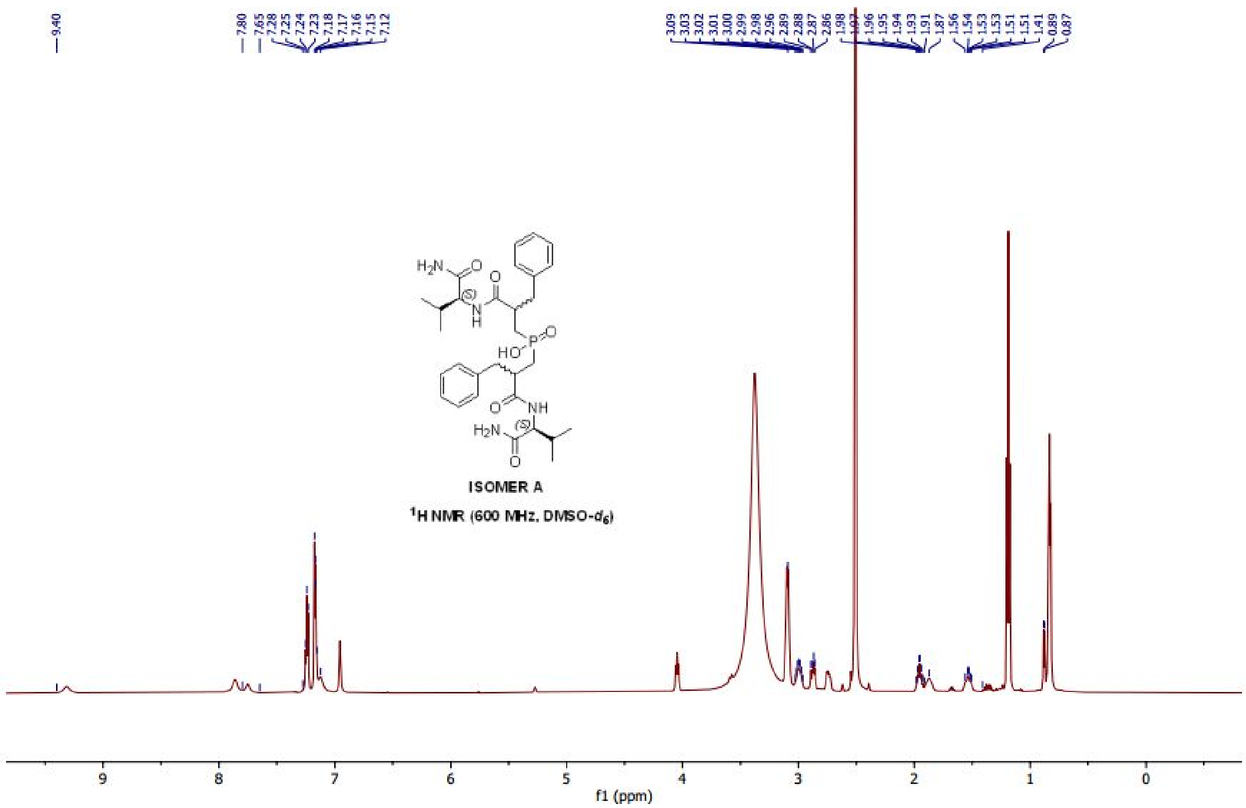

*Figure S67. ^{1}HNMR of Isomer **A** (**9c**)*

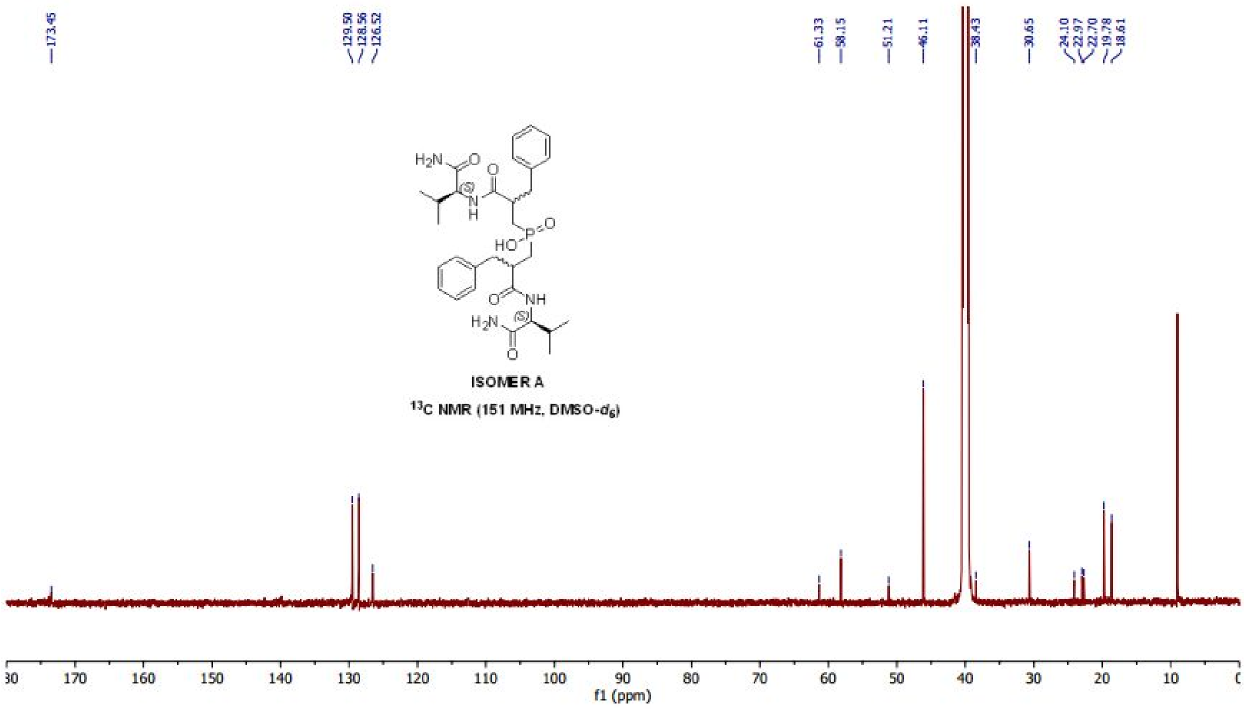

Figure S68. ^{13}*CNMR of Isomer* ***A*** *(**9c**)*

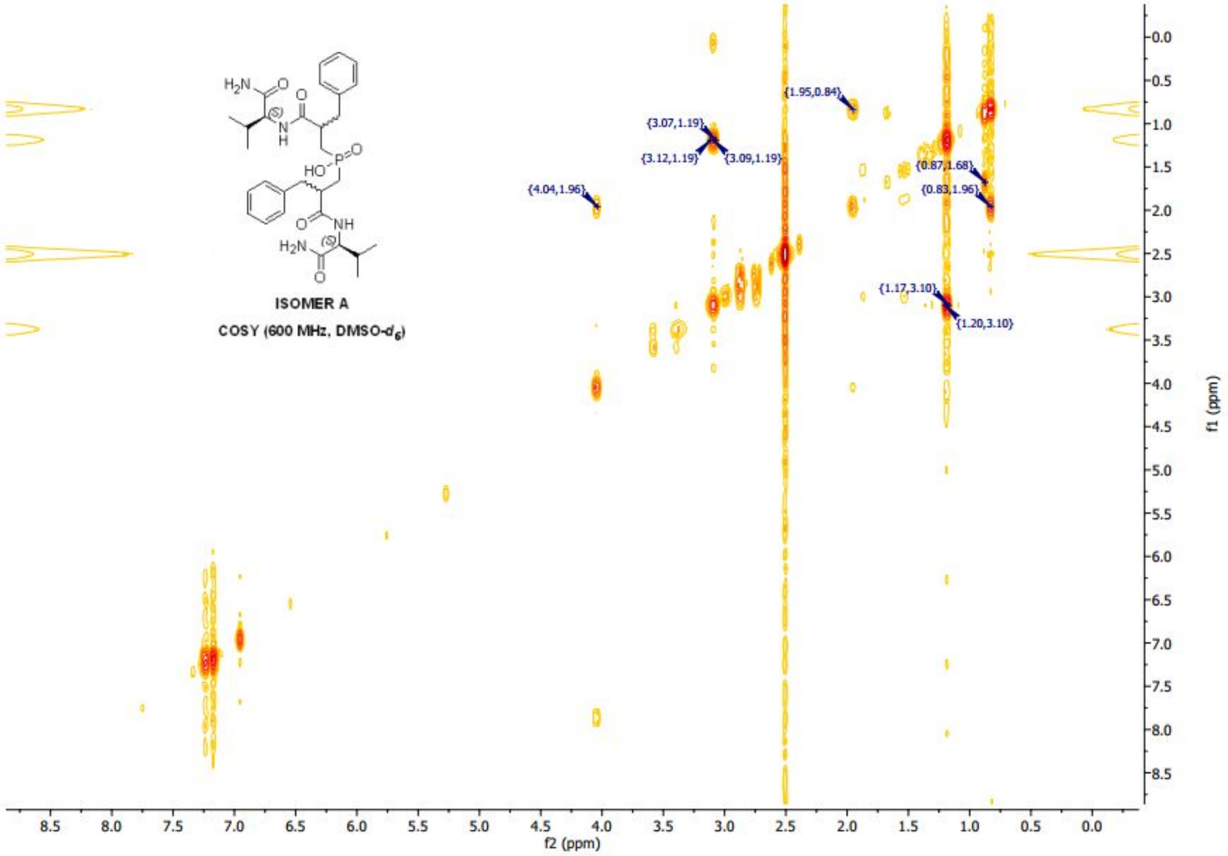

*Figure S69. COSY NMR of Isomer **A** (**9c**)*

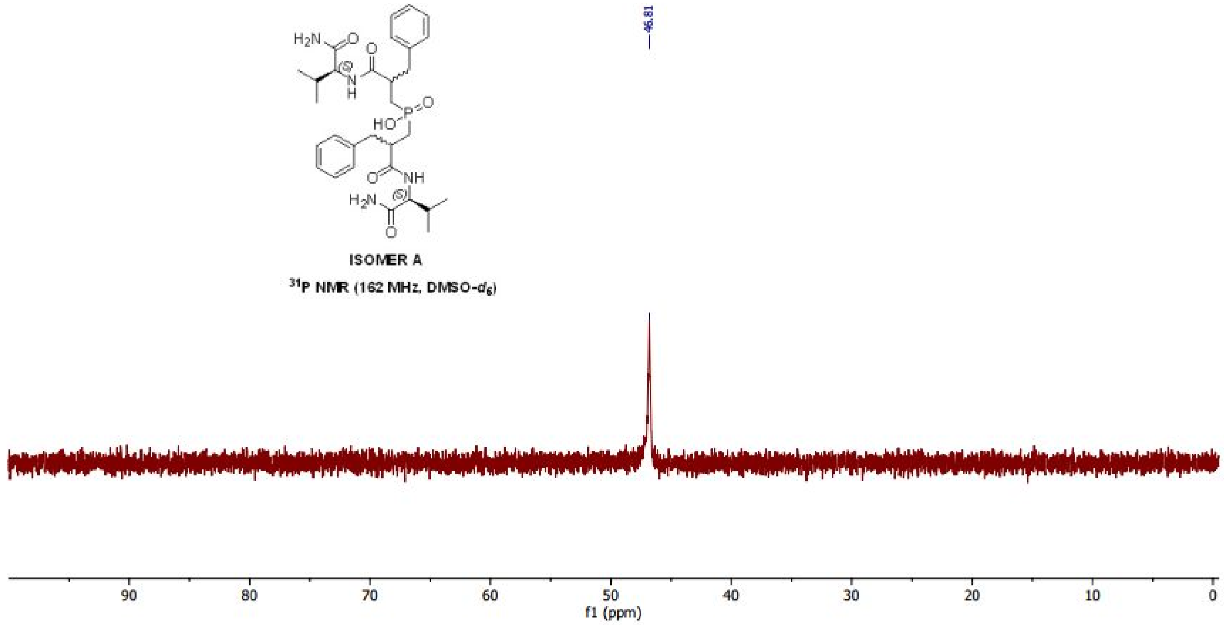

*Figure S70. ^{31}PNMR of Isomer **A** (**9c**)*

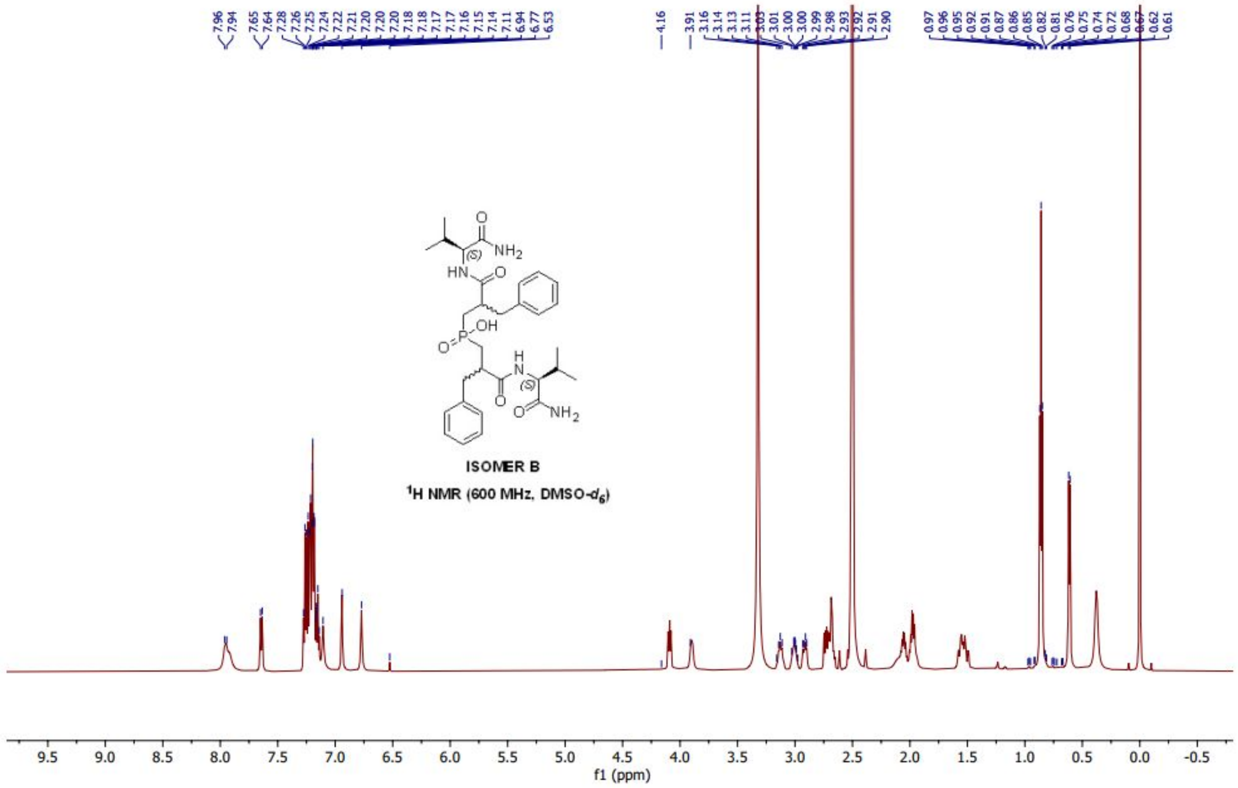

*Figure S71. ^{1}HNMR of Isomer **B** (**9c**)*

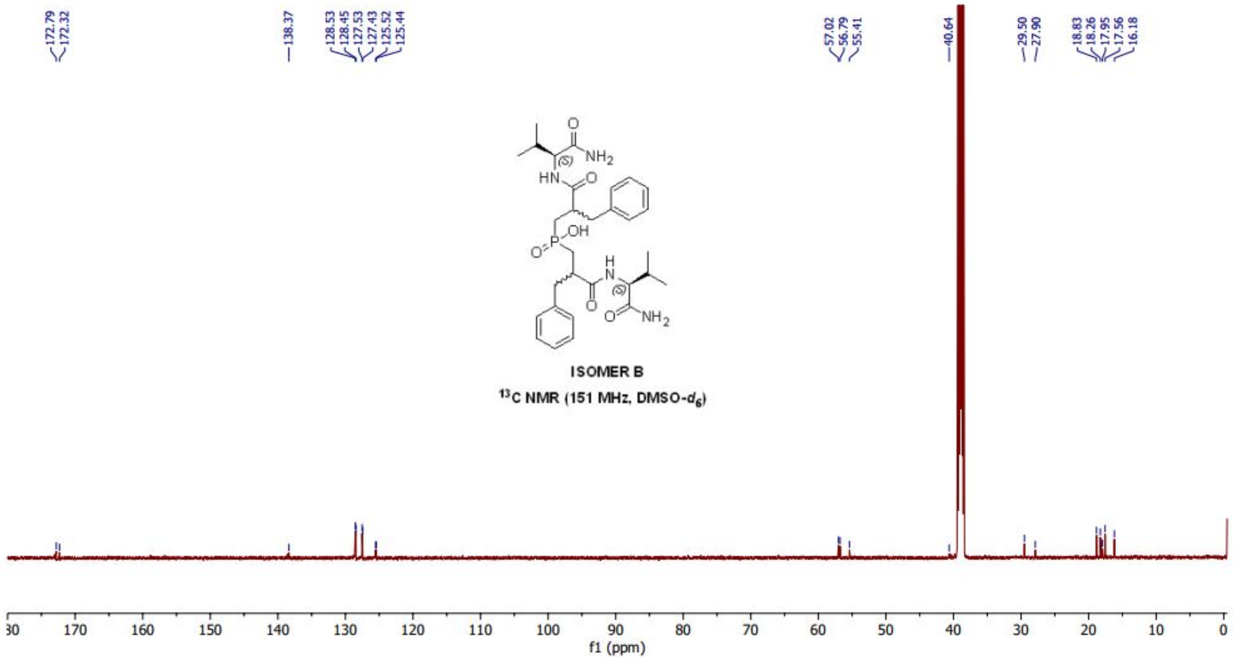

*Figure S72. ^{13}CNMR of Isomer **B** (**9c**)*

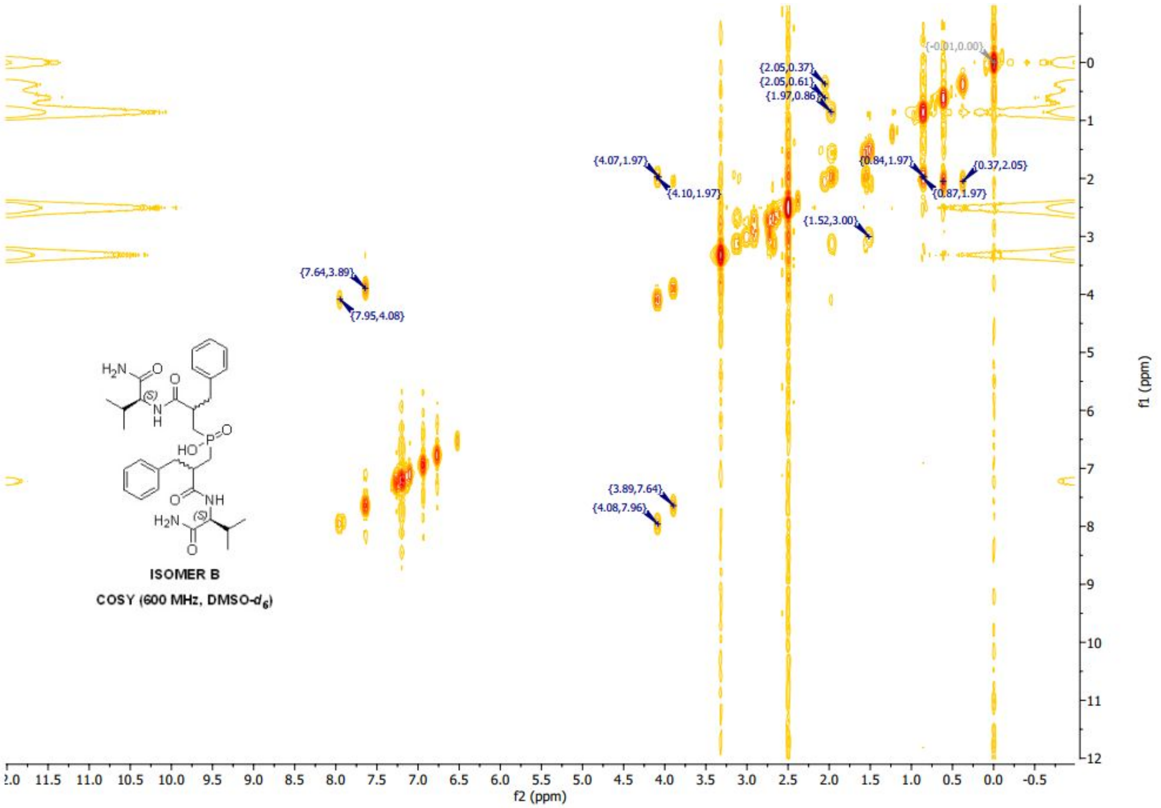

*Figure S73. COSY NMR of Isomer **B** (**9c**)*

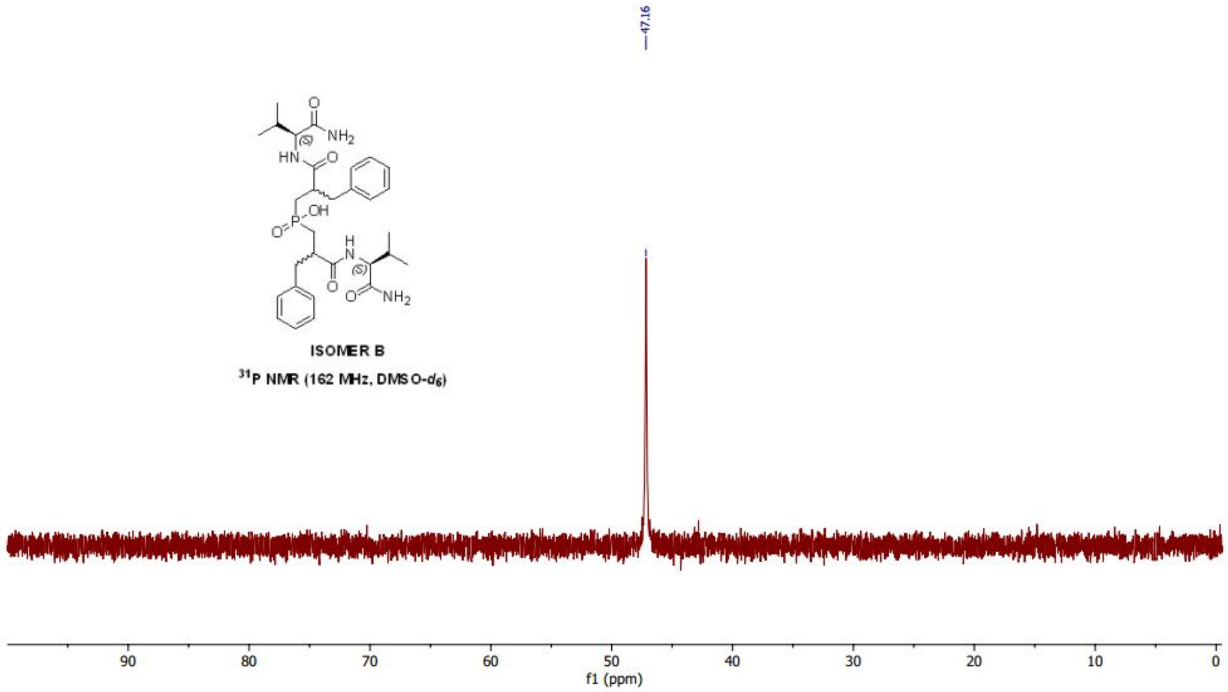

*Figure S74. ^{31}PNMR of Isomer **B** (**9c**)*

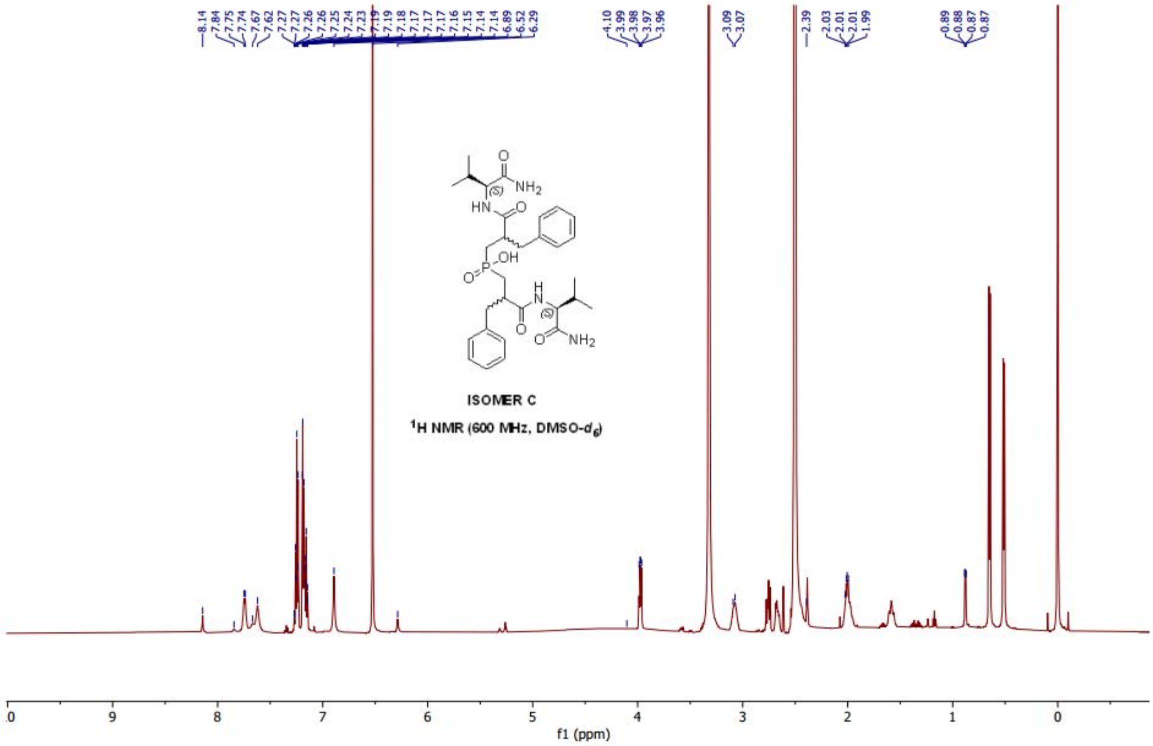

*Figure S75. ^{1}HNMR of Isomer **C** (**9c**)*

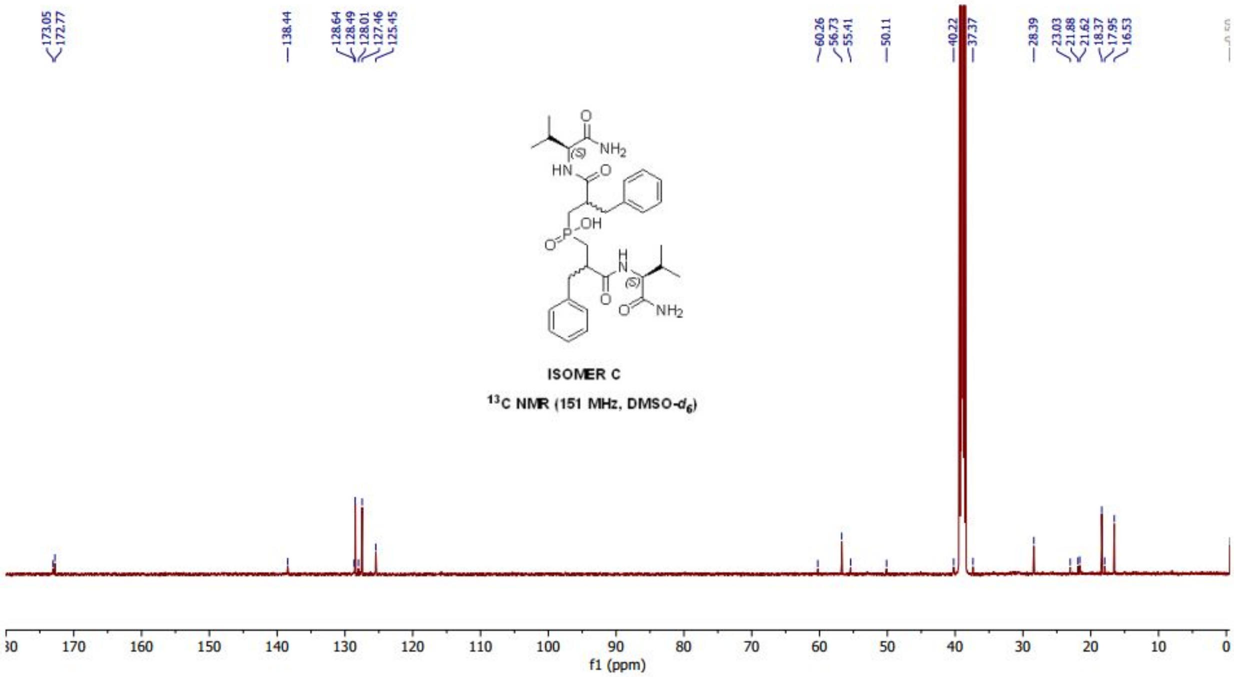

*Figure S76. ^{13}CNMR of Isomer **C** (**9c**)*

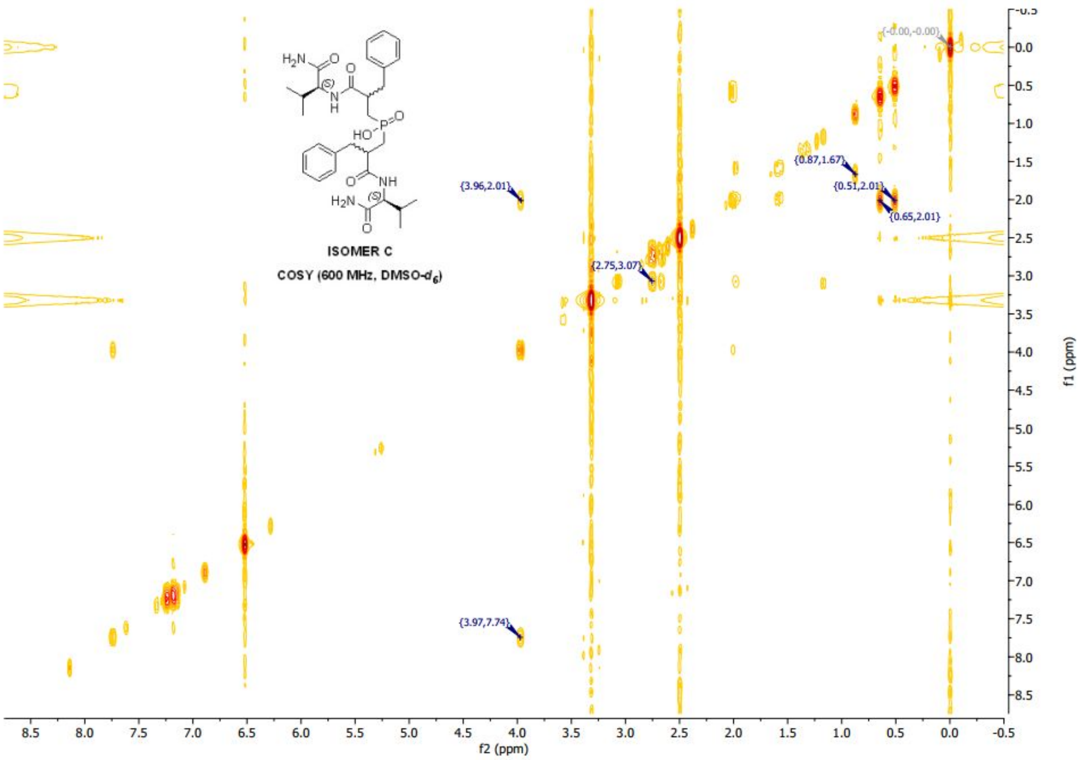

*Figure S77. COSY NMR of Isomer **C** (**9c**)*

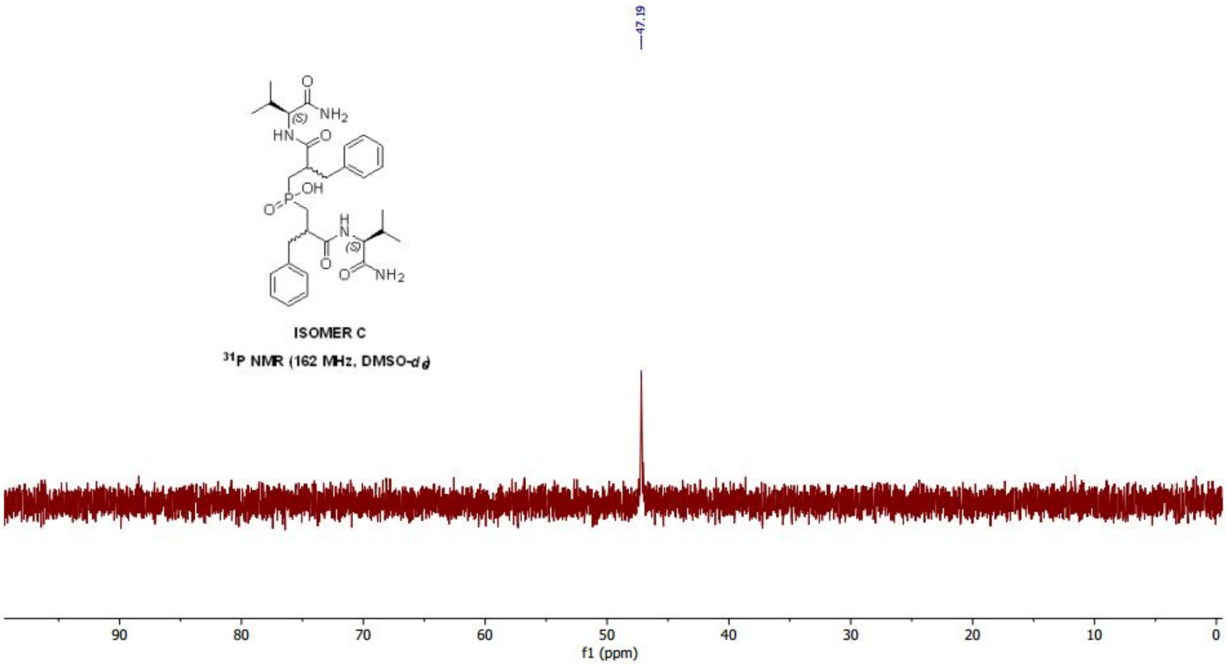

*Figure S78. ^{31}PNMR of Isomer **C** (**9c**)*
